# Synthesis of Ultra‐Incompressible and Recoverable Carbon Nitrides Featuring CN_4_ Tetrahedra

**DOI:** 10.1002/adma.202308030

**Published:** 2023-12-10

**Authors:** Dominique Laniel, Florian Trybel, Andrey Aslandukov, Saiana Khandarkhaeva, Timofey Fedotenko, Yuqing Yin, Nobuyoshi Miyajima, Ferenc Tasnádi, Alena V. Ponomareva, Nityasagar Jena, Fariia Iasmin Akbar, Bjoern Winkler, Adrien Néri, Stella Chariton, Vitali Prakapenka, Victor Milman, Wolfgang Schnick, Alexander N. Rudenko, Mikhail I. Katsnelson, Igor A. Abrikosov, Leonid Dubrovinsky, Natalia Dubrovinskaia

**Affiliations:** ^1^ Centre for Science at Extreme Conditions and School of Physics and Astronomy University of Edinburgh Edinburgh EH9 3FD UK; ^2^ Material Physics and Technology at Extreme Conditions Laboratory of Crystallography University of Bayreuth 95440 Bayreuth Germany; ^3^ Department of Physics Chemistry and Biology (IFM) Linköping University Linköping SE‐581 83 Sweden; ^4^ Bayerisches Geoinstitut University of Bayreuth 95440 Bayreuth Germany; ^5^ Photon Science Deutsches Elektronen‐Synchrotron Notkestrasse 85 22607 Hamburg Germany; ^6^ State Key Laboratory of Crystal Materials Shandong University Jinan 250100 China; ^7^ Materials Modeling and Development Laboratory NUST “MISIS” Moscow 119049 Russia; ^8^ Institut für Geowissenschaften Abteilung Kristallographie Johann Wolfgang Goethe‐Universität Frankfurt Altenhöferallee 1 D‐60438 Frankfurt am Main Germany; ^9^ Center for Advanced Radiation Sources University of Chicago Chicago IL 60637 USA; ^10^ Dassault Systèmes BIOVIA Cambridge CB4 0FJ UK; ^11^ Department of Chemistry University of Munich (LMU) Butenandtstrasse 5–13 81377 Munich Germany; ^12^ Radboud University Institute for Molecules and Materials Heijendaalseweg 135 Nijmegen 6525 AJ The Netherlands

**Keywords:** 3D frameworks of CN_4_ tetrahedra, ambient conditions recoverability, carbon nitrides, diamond anvil cell, high pressure syntheses, single‐crystal X‐ray diffraction, superhardness, ultra‐incompressibility

## Abstract

Carbon nitrides featuring three‐dimensional frameworks of CN_4_ tetrahedra are one of the great aspirations of materials science, expected to have a hardness greater than or comparable to diamond. After more than three decades of efforts to synthesize them, no unambiguous evidence of their existence has been delivered. Here, the high‐pressure high‐temperature synthesis of three carbon–nitrogen compounds, *tI*14‐C_3_N_4_, *hP*126‐C_3_N_4_, and *tI*24‐CN_2_, in laser‐heated diamond anvil cells, is reported. Their structures are solved and refined using synchrotron single‐crystal X‐ray diffraction. Physical properties investigations show that these strongly covalently bonded materials, ultra‐incompressible and superhard, also possess high energy density, piezoelectric, and photoluminescence properties. The novel carbon nitrides are unique among high‐pressure materials, as being produced above 100 GPa they are recoverable in air at ambient conditions.

## Graphical abstract

Fully saturated polymeric carbon nitrides are one of the holy grails of material sciences due to their expected exceptional superhardness. Here, the high‐pressure synthesis of three novel carbon nitrides comprised of corner‐sharing CN_4_ tetrahedra is reported. They are recoverable to ambient conditions and stable in air, feature ultra‐incompressibility, superhardness, and other attractive properties such as piezoelectricity and fluorescence.

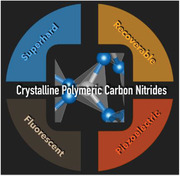

## 1 Introduction

In 1989, it was predicted that “hypothetical covalent solids formed between carbon and nitrogen are good candidates for extreme hardness”.^[^ ^1^ ^]^ A C_3_N_4_ solid isostructural to β‐Si_3_N_4_, chosen by Liu and Cohen^[^ ^1^ ^]^ as a prototype system, was calculated to have a bulk modulus of 427 GPa,^[^ ^1^ ^]^ rivaling that of diamond (446 GPa).^[^ ^2^ ^]^ These predictions sparked an enormous interest and resulted in numerous experimental and theoretical investigations.^[^ ^3^, ^4^, ^5^, ^6^, ^7^, ^8^, ^9^, ^10^, ^11^, ^12^, ^13^, ^14^, ^15^, ^16^, ^17^, ^18^, ^19^, ^20^, ^21^, ^22^, ^23^, ^24^, ^25^, ^26^ ^]^ It was suggested that the initially considered β‐Si_3_N_4_‐type C_3_N_4_^[^ ^1^ ^]^ is thermodynamically unfavorable compared to other C—N compounds.^[^ ^4^, ^23^ ^]^ Three possible stoichiometries, CN, C_3_N_4,_ and CN_2_, were proposed for stable carbon nitrides, all featuring 3D polymeric structures in which C and N atoms are fourfold‐ and threefold‐coordinated, respectively. In addition to their outstanding mechanical properties, these solids are also expected to have other useful characteristics, such as high thermal conductivity, wide bandgaps, and exotic electronic properties,^[^ ^4^ ^]^ emphasizing the exceptional multifunctional potential of this class of materials.

Intense efforts were devoted to synthesizing these promising carbon nitrides. Various experimental techniques were employed, in particular chemical (CVD) and physical (PVD) vapor depositions,^[^ ^27^ ^]^ solvothermal methods,^[^ ^9^ ^]^ and static and dynamic high‐pressure methods.^[^ ^3^, ^7^, ^8^, ^10^, ^13^, ^15^, ^17^, ^28^, ^29^ ^]^ In spite of these extensive studies, there is but a single report on the formation of a fully saturated *sp* ^3^‐hybridized carbon nitride, namely the CN compound, obtained in a diamond anvil cell laser‐heated to 7000 K above 55 GPa.^[^ ^3^ ^]^ The structure was proposed based on the match of the observed powder diffraction peaks with those of the theoretically predicted β‐InS type CN compound.^[^ ^3^ ^]^ Rietveld refinement was not possible because of “preferred orientation effects and strongly anisotropic peak broadening”.^[^ ^3^ ^]^ Moreover, this CN compound was reported non‐recoverable to ambient conditions and found unstable below 15 GPa.^[^ ^3^ ^]^

Here, we demonstrate the synthesis of four covalent carbon nitrides—one with the CN chemical composition (*oP*8‐CN), two with the C_3_N_4_ composition (*tI*14‐C_3_N_4_, *hP*126‐C_3_N_4_) and one with the CN_2_ composition (*tI*24‐CN_2_). These are designated in their Pearson notation. While *oP*8‐CN had been experimentally produced before,^[^ ^3^ ^]^ *hP*126‐C_3_N_4_, *tI*14‐C_3_N_4_, and *tI*24‐CN_2_ are hitherto unobserved although the two latter have been predicted by density functional theory (DFT) calculations.^[^ ^4^, ^24^ ^]^ Their synthesis was realized at pressures between 72 and 134 GPa and at temperatures near 2500 K in laser‐heated diamond anvil cells. The crystal structures of *tI*14‐C_3_N_4_, *hP*126‐C_3_N_4_, *tI*24‐CN_2_, and *oP*8‐CN were solved and refined using synchrotron single‐crystal X‐ray diffraction (SC‐XRD). The three novel carbon nitrides, *tI*14‐C_3_N_4_, *hP*126‐C_3_N_4_, and *tI*24‐CN_2_, feature *sp* ^3^‐hybridized carbon and corner‐sharing CN_4_ tetrahedra forming 3D frameworks. Their ultra‐incompressibility was established experimentally. All four carbon nitrides are found to persist in air at ambient conditions and to possess multifunctional properties revealed both experimentally and on the basis of DFT calculations.

## 2 Synthesis and Structural Characterization

**Figure 1:**
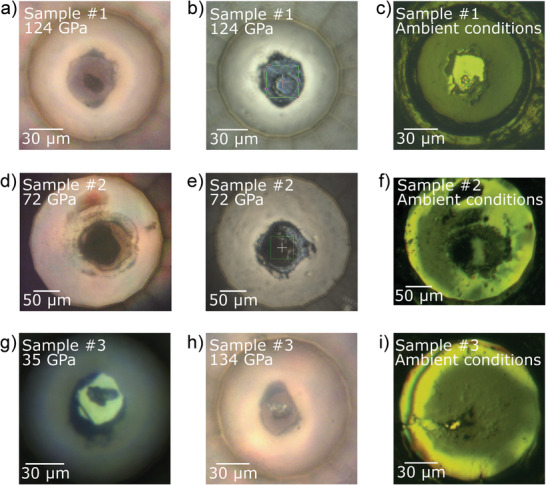
Microphotographs of a–c) sample #1, d–f) sample #2, and g–i) sample #3. The images were taken with different microscopes, illumination, and diamond anvil cell (DAC) orientation. a) Sample #1 before laser heating at 124 GPa. b) Sample #1 after laser heating at 124 GPa. The edges of the green square are 20 µm in length. c) Sample #1 at ambient pressure and temperature, after fully opening the DAC and hand‐closing it back. d) Sample #2 before laser heating at 72 GPa. e) Sample #2 after laser heating at 72 GPa. The edges of the green square are 20 µm in length. f) Sample #2 at ambient pressure and temperature, after fully opening the DAC and hand‐closing it back. g) Non‐heated sample #3 at 35 GPa. h) Laser‐heated sample #3 at 134 GPa. i) Sample #3 at ambient pressure and temperature, after fully opening the DAC and hand‐closing it back. The sample chamber collapsed during the decompression, between 86 and 18 GPa. Nonetheless, *oP*8‐CN single crystals were still found in the remaining sample chamber.

**Figure 2:**
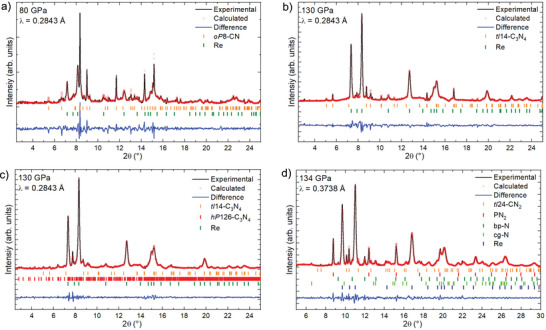
Le Bail analysis of the XRD patterns of samples containing a) *oP*8‐CN (sample #2, 80 GPa), b) *tI*14‐C_3_N_4_ (sample #1, 130 GPa), c) *hP*126‐C_3_N_4_ as well as *tI*14‐C_3_N_4_ (sample #1, 130 GPa), and d) *tI*24‐CN_2_ (sample #3, 134 GPa). The unit cells of PN_2_,^[^ ^32^ ^]^ bp‐N,^[^ ^33^ ^]^ and cg‐N^[^ ^34^ ^]^ match well the literature data. The unit cell parameter and the structures of the four carbon nitrides for the whole diffraction pattern profile fitting were obtained from the single‐crystal XRD data presented in Tables S1–S8, Supporting Information.

For the synthesis of carbon–nitrogen compounds in a pressure range of about 70 to 140 GPa, six screw‐type BX90 diamond anvil cells (DACs)^[^ ^30^ ^]^ were prepared (see Experimental Section). As summarized in **Table** **1**, the six samples (#1–6) were prepared using three different sets of precursors: i) Tetracyanoethylene (TCNE, C_6_N_4_) in a mixture with nitrogen (N_2_) in samples #1, #2, and #4; ii) Cyanuric triazide (CTA, C_3_N_12_) in sample #5; iii) Pure N_2_ with a laser light absorber (either black phosphorus or boron‐doped diamond), with the diamond anvil used as the carbon source, in samples #3 and #6, this configuration testing if carbon nitrides can be formed from the pure elements. All samples were laser‐heated at their target pressure. It is worth noticing that CTA and TCNE have previously been investigated in the diamond anvil cell and laser‐heated.^[^ ^25^, ^31^ ^]^ However, in both cases, laser heating was done at insufficiently high pressures (42.0 GPa for CTA and 30 GPa for TCNE) to produce carbon nitrides featuring 3D frameworks of CN_4_ tetrahedra. **Figure** **1** shows microphotographs of samples #1–3 before and after laser‐heating. Chemical reactions occurred in all samples, resulting in the formation of four carbon nitrides: *tI*14‐C_3_N_4_, *hP*126‐C_3_N_4_, *oP*8‐CN, and *tI*24‐CN_2_. *tI*14‐C_3_N_4_ and *hP*126‐C_3_N_4_ were produced from different precursors (Table 1). Synchrotron SC‐XRD from high‐quality crystallites at high pressures was used for the structure solution and refinement (Tables S1–S6 and Figures S1–S4, Supporting Information). The crystallographic data for each compound was deposited in the ICSD database. A summary of the average interatomic distances is presented in Table S7, Supporting Information. Le Bail analyses of the integrated diffraction patterns demonstrate that all XRD reflections from the studied samples are explained by the reported carbon nitrides and other known phases (**Figure** **2**).

**Table 1:** Summary of experiments performed for the synthesis of C—N compounds. The pressure values provided in the table are those at which the C—N compounds were observed after laser‐heating at a given temperature. The uncertainty in pressure and temperature measurements are of ±5 GPa and ±200 K, respectively. Various measurements performed on the synthesis products are designated as follows: single‐crystal X‐ray diffraction (SC‐XRD), Raman spectroscopy (Raman), photoluminescence (PL), transmission electron microscopy (TEM), selected area electron diffraction (SAED), electron energy loss spectroscopy (EELS), and diamond indentation test (diamond indentation)

Sample	Culet size [µm]	Precursors	Synthesis conditions		C—N reaction products	Measurements
			Pressure [GPa]	Temperature [K]		
#1	80	TCNE + N_2_	124	2700	*tI*14‐C_3_N_4_ and *hP*126‐C_3_N_4_	SC‐XRD, Raman, PL, TEM, SAED, EELS
#2	120	TCNE + N_2_	72	2600	*oP*8‐CN	SC‐XRD, Raman, PL
#3	80	^a)^N_2_ + carbon from diamond anvils	134	^b)^∼2500	*tI*24‐CN_2_	SC‐XRD, Raman
#4	120	TCNE + N_2_	120	2500	^c)^ *tI*14‐C_3_N_4_ and *hP*126‐C_3_N_4_	Diamond indentation
#5	120	CTA	74	2700	*hP*126‐C_3_N_4_	SC‐XRD, PL
#6	120	N_2_ + boron‐doped diamond	110	4350	*tI*14‐C_3_N_4_	SC‐XRD

^a)^ In this experiment black phosphorus was used as a laser light absorber, while diamond anvils themselves served as a source of carbon in a reaction with nitrogen. PN_2_ appeared in this cell in addition to tI24‐CN_2_, but no products of a possible reaction between C, N, and P were found. The procedure to obtain the black phosphorus precursor is described in ref.[32];

^b)^ In this experiment temperature could not be measured and its value was estimated from the observed thermoemission;

^c)^ In this experiment (#4) the P‐T conditions were similar to those in experiment #1, and the reaction products are supposed to be the same in both cases.

**Figure 3:**
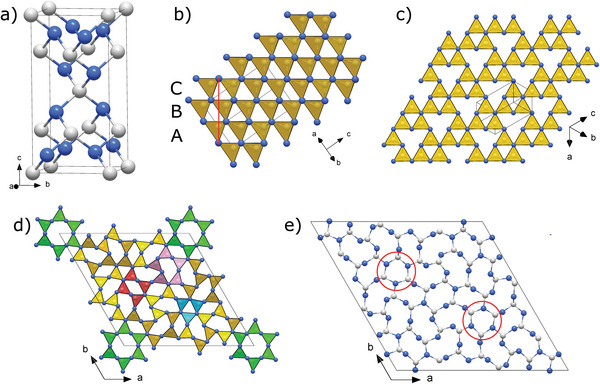
Crystal structures of *tI*14‐C_3_N_4_ and *hP*126‐C_3_N_4_ at 124 GPa. a) Unit cell of *tI*14‐C_3_N_4_. b) View of the polyhedral model of the *tI*14‐C_3_N_4_ structure along the [110] direction; the structure built of corner‐sharing CN_4_ tetrahedra can be interpreted as an ABC stacking of layers (oriented perpendicular to the page) in the [1$\bar{1}$2] direction (marked by a red line); c) A single layer viewed along the [1$\bar{1}$2] direction. d) A polyhedral model of *hP*126‐C_3_N_4_ viewed along the *c* direction. The green, pink, red, and teal sets of tetrahedra highlight the six‐, five‐, four‐, and three‐membered groups of CN_4_ tetrahedra. e) A projection of the unit cell of *hP*126‐C_3_N_4_ on the *ab* plane; Flat C_3_N_3_ rings, highlighted by red circles, are reminiscent of triazine rings in heptazine and its derivatives, albeit solely composed of single bonds. Grey and blue spheres represent carbon and nitrogen atoms, respectively.

All four carbon nitrides consist of fully saturated carbon and nitrogen atoms—that is, all carbon atoms form four single bonds and all nitrogen atoms three single bonds (Table S7, Supporting Information). As seen in **Figure** **3**, both *tI*14‐C_3_N_4_ and *hP*126‐C_3_N_4_ are built of corner‐sharing CN_4_ tetrahedra composed exclusively of C—N bonds. The structure of *tI*14‐C_3_N_4_ can be described as having incomplete layers of tetrahedra stacked in an ABC sequence in the [1$\bar{1}$2] (or equivalent) direction (Figure 3b), where each fourth tetrahedron in a row is missing (Figure 3c). The structure of *hP*126‐C_3_N_4_ is much more complex than that of *tI*14‐C_3_N_4_ (Figure 3d,e). Its corner‐sharing CN_4_ tetrahedra form a very complicated arrangement of interlinked rings of tetrahedra, including six‐membered rings (i.e.*, sechser*‐rings, according to F. Liebau's^[^ ^35^ ^]^ nomenclature) forming large empty channels in the *c*‐direction—as well as *fünfer*‐, *vierer*‐, and *dreier*‐membered rings. Also, three carbon and three nitrogen atoms laying in the same *ab* plane form flat C_3_N_3_ rings, reminiscent of triazine rings in heptazine and its derivatives (highlighted by red circles in Figure 3e), albeit solely composed of single bonds. *hP*126‐C_3_N_4_ is structurally closely related to β‐Si_3_N_4_^[^ ^36^ ^]^ although not isotypic.

**Figure 4:**
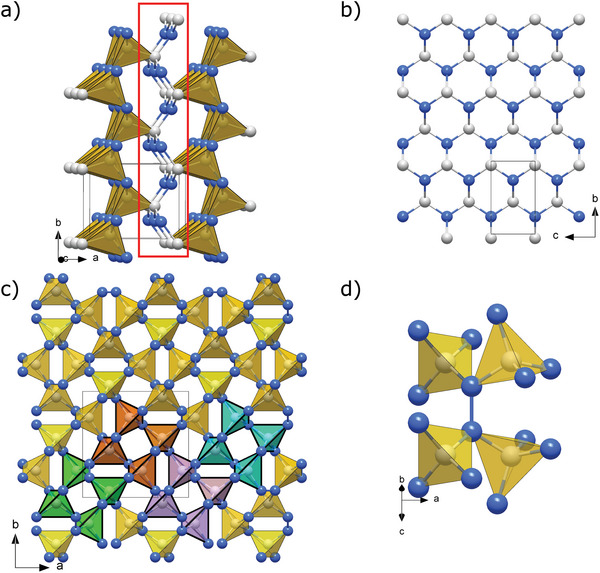
Crystal structures of *oP*8‐CN and *tI*24‐CN_2_ at 72 and 134 GPa, respectively. a) A polyhedral model of the *oP*8‐CN structure built of C‐CN_3_ tetrahedra. The tetrahedra sharing nitrogen vertices form corrugated layers laying in the bc plane, which are connected through carbon apexes of the tetrahedra by triply coordinated nitrogen atoms, as highlighted by the red rectangle. b) A corrugated honeycomb‐like net of the 1:1 C:N composition formed by the atoms connecting the layers. c) A polyhedral model of the *tI*24‐CN_2_ structure (viewed along the c direction) built of corner‐sharing CN_4_ tetrahedra linked with each other through N_2_ dimers. To emphasize that the crystal structure can be understood as repeating units composed of four CN_4_ tetrahedra, four of these units are drawn in different colors (green, orange, purple, and teal). d) An example of an N_2_ dimer (oriented vertically in the figure) connecting two pairs of corner‐sharing CN_4_ tetrahedra. Grey and blue spheres represent carbon and nitrogen atoms, respectively.

The *oP*8‐CN compound produced at 72 GPa has its atoms arranged in corrugated layers of C‐CN_3_ tetrahedra sharing N vertices and laying in the *bc* plane (**Figure** **4**a). Carbon apexes of the tetrahedra point toward neighboring layers connected through triply coordinated nitrogen atoms, thus forming in the *bc* plane a corrugated honeycomb‐like net of the 1:1 C:N composition (Figure 4b). This compound was previously reported to be produced by Stavrou et al.,^[^ ^3^ ^]^ formed by the laser‐heating of graphite in molecular nitrogen at pressures above 55 GPa and 7000 K,^[^ ^3^ ^]^ although they could not refine its crystal structure.

One more C—N phase, *tI*24‐CN_2_, has a structure (Figure 4c,d) that can be presented as a framework of corner‐sharing CN_4_ tetrahedra interlinked through N_2_ dimers—nitrogen atoms make two C—N bonds and one N–N bond.

In all four compounds at pressures of their synthesis, the average N–C—N (or C–C—N) bond angle is, within uncertainty, 109.5°, perfectly matching the value expected of an ideal tetrahedron and highlighting the *sp* ^3^‐hybridization of the carbon atoms. In *hP*126‐C_3_N_4_, *oP*8‐CN, and *tI*24‐CN_2_, the average C—N–C (or C—N–N) bond angles of 118.3(1)°, 118.62(3)°, and 117.9(4)°, respectively, suggest the presence of *sp* ^2^‐ and *sp* ^3^‐hybridized nitrogen, with a strong preference for the former. However, this is the opposite in *tI*14‐C_3_N_4_ where the average C—N—C bond angle is 111.9(1)°—thereby the only one out of the four carbon nitrides predominantly having *sp* ^3^‐hybridized nitrogen atoms.

Three of the carbon nitrides synthesized in this work, *tI*14‐C_3_N_4_,^[^ ^4^, ^17^ ^]^ *oP*8‐CN,^[^ ^4^, ^37^ ^]^ and *tI*24‐CN_2_,^[^ ^4^, ^24^ ^]^ have been theoretically predicted. In previous literature, these were named according to their space groups, namely *I*‐42*m*‐C_3_N_4_, *Pnnm*‐CN, and *I*‐42*d*‐CN_2_, respectively. However, *hP*126‐C_3_N_4_ was not predicted by structure search calculations, likely due to its complex structure and its large unit cell. In order to provide a self‐consistent theoretical treatment of all phases (including the unpredicted one), our own DFT calculations were performed. The relaxed theoretical structural models perfectly agree with the experimental models (Tables S2–S7, Supporting Information). The *hP*126‐C_3_N_4_ solid appears dynamically stable (Figure S5 and S6, Supporting Information), and likewise for *tI*14‐C_3_N_4_, *oP*8‐CN, and *tI*24‐CN_2_ (Figure S6, Supporting Information), even at ambient pressure.

It comes as a surprise that two same‐stoichiometry phases, *tI*14‐C_3_N_4_ and *hP*126‐C_3_N_4_, were simultaneously produced from sample #1. One possible explanation is that one of these two phases forms at higher temperatures while the other forms at lower temperatures. Due to temperature gradients during laser heating, the two phases could then be formed concomitantly. Given that at 124 GPa, the volume per atom of *hP*126‐C_3_N_4_ (4.870(4) Å^3^) is 1.4% larger than that of *tI*14‐C_3_N_4_ (4.804(2) Å^3^), and that *tI*14‐C_3_N_4_ has a slightly lower enthalpy (7.2 meV, see Figure S7, Supporting Information) than *hP*126‐C_3_N_4_, it is reasonable to assume *hP*126‐C_3_N_4_ to be the high‐temperature stable solid. The *sp* ^3^‐hybridization of all N atoms in *tI*14‐C_3_N_4_, compared to a large portion of *sp* ^2^‐hybridized N atoms in *hP*126‐C_3_N_4_, could be responsible for the higher density of the former. DFT calculations of the Gibbs free energy of both C_3_N_4_ compounds at 124 GPa (Figure S7, Supporting Information) validate this interpretation, showing that indeed *hP*126‐C_3_N_4_ is more stable at temperatures above 1486 K. It cannot be excluded that it is due to the precursors that the *tI*14‐C_3_N_4_ polymorph is found as the only product in the experiments of sample #6.

## 3 Recovery to Ambient Conditions and Equation of State

**Figure 5:**
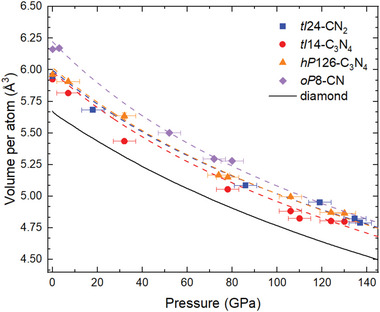
Experimental and calculated pressure dependence of the unit cell volume per atom for the *tI*14‐C_3_N_4_, *hP*126‐C_3_N_4_, *oP*8‐CN, and *tI*24‐CN_2_ solids found in this work. The square symbols represent experimental data points obtained from SC‐XRD data; the dashed lines of the corresponding colors are fits of the DFT data (see Supporting Information for details) with a third‐order Birch–Murnaghan equation of state (EOS). See Table S13, Supporting Information for the EOS parameters. The black curve is the data for the diamond,^[^ ^2^ ^]^ provided for comparison. The accuracy of the reported experimental EOS parameters should be taken with care, as the number of experimental pressure‐volume points is limited.

**Figure 6:**
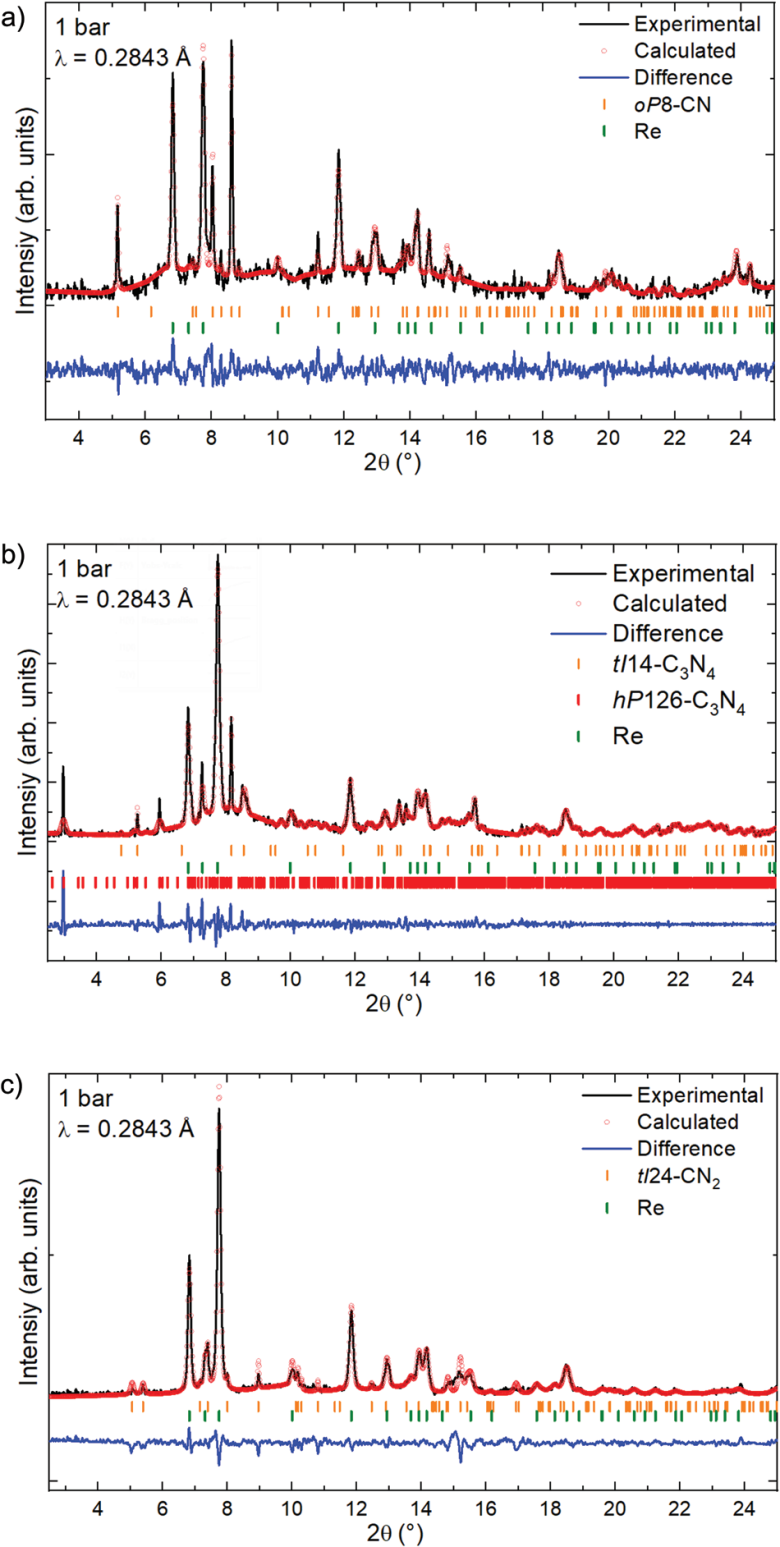
Le Bail analysis for the XRD patterns of samples containing a) *oP*8‐CN (sample #2), b) *tI*14‐C_3_N_4_ and *hP*126‐C_3_N_4_ (sample #1), and c) *tI*24‐CN_2_ (sample #3) at ambient conditions. The unit cell parameter and the structure of the four carbon nitrides for the whole diffraction pattern profile fitting were obtained from the single‐crystal XRD data presented in Tables S9–S12, Supporting Information.

Upon gradual decompression, the behavior of the carbon nitrides was monitored using SC‐XRD (**Figure** **5** and Figures S8 and S9, Supporting Information). Ambient conditions were reached by opening the DACs and exposing the samples to air. High‐quality SC‐XRD data could still be collected. These revealed that all four carbon nitrides not only sustained at ambient conditions and air but preserved their crystallinity and high‐pressure crystal structures (see the crystallographic data at 1 bar in **Table** **2** and Tables S7–S12, Supporting Information). **Figure** **6** shows a Le Bail analysis for the powder XRD pattern collected at ambient conditions on all four phases.

**Table 2:** Experimental lattice parameters for *oP*8‐CN, *tI*14‐C_3_N_4_, *hP*126‐C_3_N_4_, and *tI*24‐CN_2_ at ambient conditions, determined from single‐crystal X‐ray diffraction measurements

Phase	Space group	*a* [Å]	*b* [Å]	*c* [Å]	Volume [Å^3^]
*oP*8‐CN	*Pnnm*	5.282(4)	3.9353(12)	2.3719(9)	49.30(4)
*tI*14‐C_3_N_4_	*I*‐42*m*	3.4680(6)	3.4680(6)	6.8970(16)	82.95(3)
*hP*126‐C_3_N_4_	*P*6_3_ */m*	18.978(8)	18.978(8)	2.4083(11)	751.20(5)
*tI*24‐CN_2_	*I*‐42*d*	6.4559(13)	6.4559(13)	3.426(2)	142.79(9)

A small piece of the recovered sample #1 was extracted and prepared for transmission electron microscopy (TEM) measurements using a focused ion beam (FIB) milling apparatus (Figure S10, Supporting Information). A crystalline phase was identified from the TEM images (Figure S11, Supporting Information), on which selected area electron diffraction (SAED) measurements were performed (Figure S12, Supporting Information). The observed reflections were found to be consistent with the tetragonal unit cell of *tI*14‐C_3_N_4_ determined from SC‐XRD at 1 bar (Table S10, Supporting Information). Moreover, electron energy loss spectroscopy (EELS, Figure S13, Supporting Information) on this crystal revealed the presence of *sp* ^3^‐carbon and *sp* ^3^‐nitrogen in a ratio of 3:4—all in agreement with the properties already identified for *tI*14‐C_3_N_4_.

The *P–V* data obtained on decompression in the whole pressure range studied (Table S8, Supporting Information) was fitted using the second‐order Birch–Murnaghan equation of state^[^ ^38^ ^]^ with *V*_0_ fixed on the experimental values at 1 bar (Figure 5 and Figures S8 and S9, Supporting Information). This revealed the very high bulk modulus of the four carbon nitrides, especially for *hP*126‐C_3_N_4_ (*K*_0_ = 417(6) GPa) and *tI*24‐CN_2_ (*K*_0_ = 419(8) GPa), all in agreement with the values obtained from DFT calculations (Table S13, Supporting Information). All four carbon nitrides classify as ultra‐incompressible materials, with the bulk moduli of *tI*24‐CN_2_ and *hP*126‐C_3_N_4_ being larger than that of cubic boron nitride c‐BN (395(2) GPa)^[^ ^39^ ^]^ and comparable to that of diamond (446 GPa).^[^ ^2^ ^]^

## 4 Physical Properties

**Figure 7:**
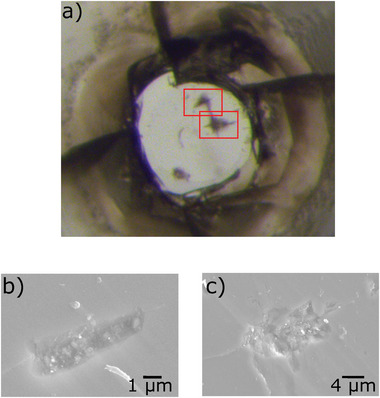
Experimental evidence of the superhardness of the C_3_N_4_ polymorphs (sample #4), which indented the diamond anvils’ surface. a) An image of one of the diamond anvils under an optical microscope (red rectangles mark the areas visualized by SEM) in (b,c).

Experimental insight into the hardness of these carbon nitrides was obtained from sample #4. This sample was prepared following the same procedure as sample #1 in order to produce *tI*14‐C_3_N_4_ and *hP*126‐C_3_N_4_. The pressure was then fully released and the gasket was removed. After that, the diamond anvils were pressed against the sample remaining on the anvils’ culet, using it to indent the anvils. As seen from the optical and scanning electron microscope (SEM) images (**Figure** **7**), this procedure resulted in indenting the diamond anvil. This qualitatively demonstrates the *tI*14‐C_3_N_4_ and *hP*126‐C_3_N_4_ solids to have a hardness comparable to that of diamond itself.^[^ ^40^ ^]^ The hardness of the four carbon nitrides was also investigated using DFT calculations. As seen in Table S14, Supporting Information, all four compounds are found to belong to the class of superhard materials (i.e., hardness greater than 40 GPa), with hardness values comparable to either c‐BN or diamond, depending on the employed hardness model.

The recovery of complex materials synthesized above 100 GPa is a unique case—to the best of our knowledge a similar one has been never reported—and opens up new perspectives for high‐pressure materials science in general. The carbon nitrides synthesized in this work are expected to exhibit multiple exceptional functionalities besides their mechanical properties, with the potential to be engineering materials in the same category as diamonds.^[^ ^41^ ^]^ An insight into the possible prospects of these solids comes from our experiments, and from further theoretical calculations. In particular, sample #1, containing *tI*14‐C_3_N_4_ and *hP*126‐C_3_N_4_, appears visually transparent (Figure 1), pointing to insulating properties and wide band gaps of the compounds. This assessment is further supported by theoretical calculations at ambient conditions (Table S14 and Figures S14–S17, Supporting Information), showing that *tI*14‐C_3_N_4_ and *hP*126‐C_3_N_4_, but also *oP*8‐CN and *tI*24‐CN_2_, have wide band gaps (between 4.3 and 5.4 eV) comparable to diamond (5.48 eV).^[^ ^42^ ^]^ However, contrary to diamond, *tI*24‐CN_2_ is found to have a direct band gap. In addition, in the electronic structure of *tI*14‐C_3_N_4_ and *hP*126‐C_3_N_4_, one can clearly identify flat bands at the top of the valence band (Figures S15 and S16, Supporting Information). A theoretical analysis shows that the degree of the electron–electron correlation effects in *tI*14‐C_3_N_4_ for the separated valence bands in the region from −3 to 0 eV is comparable to those in *3d‐*transition metals, like Ni^[^ ^43^ ^]^ (see Supporting Information, electron–electron correlation effects, Figure S18, Supporting Information). Moreover, at a relatively low hole doping, a van Hove singularity in the hole density of states (Figure S15, Supporting Information) may lead to numerous competing channels of instabilities, such as charge density waves, itinerant magnetism, and others, with a very non‐trivial interplay between them,^[^ ^44^, ^45^ ^]^ making such systems highly attractive for further studies. Evidence supporting superconductivity below ≈55 K at ambient pressure in boron‐doped *tI*14‐C_3_N_4_ has been presented.^[^ ^46^ ^]^

The wide band gap nature of these materials, combined with the variability of their chemical compositions and crystal structures, leads to distinctive local chemical environments of the C and N atoms. From these environments, one can expect different properties of native and external defects. Photoluminescence measurements were performed on sample #1 (*tI*14‐C_3_N_4_ and *hP*126‐C_3_N_4_), sample #2 (*oP*8‐CN), and sample #5 (*hP*126‐C_3_N_4_) at ambient conditions using a green laser (532 nm, 2.33 eV) or a red laser (632.8 nm, 1.96 eV) as the excitation source. As seen in Figure S19, Supporting Information, from all of these samples very strong photoluminescence is observed. Given that the excitation energy is significantly smaller than the expected band gap of these materials, this directly suggests the presence of color centers and the possible tunability of the photoluminescence through defects.

Since the structures of *tI*14‐C_3_N_4_ and *tI*24‐CN_2_ are non‐centrosymmetric, the compounds can exhibit piezoelectric properties. To gain further insight, DFT calculations were performed, which resulted in piezoelectric coefficients of −0.77 and −0.35 C m^−2^ for *tI*14‐C_3_N_4_ and *tI*24‐CN_2_, respectively (Table S15, Supporting Information). These values are two to four times greater than those of *α*‐quartz (0.171 C m^−2^),^[^ ^47^ ^]^ which is a standard piezoelectric material. The combination of piezoelectricity and superhardness distinguishes these two carbon nitrides from diamond and cubic boron nitride, and they could potentially be of use for smart and resilient cutting tools.^[^ ^48^ ^]^ The non‐centrosymmetric nature of the structure of these compounds also gives rise to non‐linear optical properties such as second‐order harmonic generation, as demonstrated from visual observations in Figure S20, Supporting Information.

Energy density calculations were performed for the four C—N compounds with respect to decomposition into graphite and molecular nitrogen, at ambient conditions. They revealed that they have a high gravimetric energy density, comparable to or higher than that of TNT for *oP*8‐CN, *tI*14‐C_3_N_4_, and *hP*126‐C_3_N_4_, and for *tI*24‐CN_2_, a value even higher than for RDX (Table S16, Supporting Information).

## 5 Conclusion

The high‐pressure high‐temperature synthesis and ambient conditions recovery of the ultra‐incompressible *tI*14‐C_3_N_4_, *hP*126‐C_3_N_4_, *oP*8‐CN, and *tI*24‐CN_2_ solids is the long‐awaited response to a three‐decade‐old quest for alternatives to diamond and c‐BN. This study provides the impetus to further explore the rich chemistry of the carbon–nitrogen system and firmly establishes the exceptional mechanical and electronic properties of these materials. The discovery of C_3_N_4_ compounds with fourfold‐coordinated carbon atoms could also serve as a key starting point for the search for a solid featuring the thus far elusive six‐fold coordinated carbon atom,^[^ ^49^ ^]^ analogous to silicon in the high‐pressure compound γ‐Si_3_N_4_.^[^ ^50^ ^]^ Our results prove for the first time that materials synthesized at pressures over 100 GPa ca be recovered at ambient conditions. Though immediate large‐scale industrial production of carbon nitrides at a megabar pressure range is not expected, given the current state of technology, the discovery that high‐pressure C—N compounds are metastable at ambient conditions opens up non‐trivial perspectives. Finding alternative synthesis pathways for these compounds—as done for high‐pressure LiN_5_^[^ ^51^, ^52^ ^]^ or cg‐N,^[^ ^34^, ^53^ ^]^ for example—or the use of small amounts of crystals pre‐synthesized in DACs as seeds for the growth of these phases in mild conditions in large‐volume presses or by chemical vapor deposition (CVD), are possibilities that should be closely examined.

## 6 Experimental Section

### Sample Preparation

Six BX90‐type diamond anvil cells^[^ ^30^ ^]^ equipped with diamond anvil culets from 120 to 80 µm were prepared. Samples #1, #2, and #4 were loaded with tetracyanoethylene (TCNE, C_6_N_4_, Alfa Aesar, 98% purity) and molecular nitrogen, sample #5 with cyanuric triazide (CTA, C_3_N_12_), while two others were loaded with molecular nitrogen as well as piece of black phosphorus (sample #3) and boron‐doped diamond (sample #6), serving as laser light absorbers. CTA was prepared according to the procedure described in the literature^[^ ^54^ ^]^ (see Supporting Information for details). The pressure inside of the sample chamber was measured using the first‐order Raman mode of diamond^[^ ^55^ ^]^ and verified using the calibrated diffraction lines of the rhenium gasket.^[^ ^56^ ^]^ Laser heating was performed with double‐sided Nd:YAG lasers (*λ* = 1064 nm) in the home laboratory at the Bayerisches Geoinstitut (BGI)^[^ ^57^ ^]^ as well as at the GSECARS beamline of the Advanced Photon Source (APS) using TCNE or phosphorus as laser absorbers. Temperatures were measured with an accuracy of ±200 K, using the thermoemission produced by the laser‐heated samples.^[^ ^57^ ^]^

CTA was prepared according to the procedure described in the literature.^[^ ^54^ ^]^ The product was prepared via metathesis by refluxing a 50 ml acetone solution of cyanuric chloride (C_3_Cl_3_N_3_, 0.5 mmol, 0.0915 g) and a suspension of sodium azide (NaN_3_, 1.5 mmol, 0.0975 g) for 48 h. After quickly cooling the mixture to ambient temperature, the solution was filtered to remove white solids which consisted of sodium chloride and a minimal amount of unreacted sodium azide. Crystallization was carried out by slow evaporation of the mother liquor at room temperature over a period of 24 h, yielding colorless crystals of CTA.

CTA was an energetic compound with possible sensitivities toward heat, friction, impact, and discharge. Although no incidents occurred during the experiments, personal safety equipment such as protecting helmets, face shields, ear plugs, leather coats, Kevlar gloves, and grounded shoes as well as grounded equipment were strongly recommended.

### X‐ray Diffraction Measurements and Analysis

The X‐ray diffraction studies were done at the ID27 beamline (*λ* = 0.3738 Å) and ID11 beamline (*λ* = 0.2843 Å) of the Extreme Brilliant Source European Synchrotron Radiation Facility (EBS‐ESRF), at the GSECARS beamline of the APS (*λ* = 0.2952 Å) and at the P02.2 beamline of the Deutsches Elektronen‐Synchrotron (DESY; *λ* = 0.2905 Å). In order to determine the position of the polycrystalline sample on which the single‐crystal X‐ray diffraction (SC‐XRD) acquisition was obtained, a full X‐ray diffraction mapping of the pressure chamber was achieved. The sample position displaying the most and the strongest single‐crystal reflections belonging to the phase of interest was chosen for the collection of single‐crystal data, collected in step‐scans of 0.5° from −38° to +38°. The CrysAlis^Pro^ software^[^ ^58^ ^]^ was utilized for the single crystal data analysis. The analysis procedure included the peak search, the removal of the diamond anvils’ and other “parasitic” signal contributions, finding reflections belonging to a unique single crystal, the unit cell determination, and the data integration. The Domain Auto Finder program (DAFi) was used for the quick search of subsets of reflections from individual microcrystals in the whole set of SC‐XRD data obtained from the microcrystalline multiphase samples.^[^ ^59^ ^]^ The crystal structures were then solved and refined using the OLEX2^[^ ^60^ ^]^ and JANA2006 software.^[^ ^61^ ^]^ OLEX2 was employed to obtain a preliminary structural model using SHELXT^[^ ^62^ ^]^ and JANA2006 to cull parasitic reflections (e.g., diamonds, other single‐crystals) and obtain a final structural model.

All four crystal structures were solved without any a priori knowledge, that is, without starting from the structures proposed in the literature. Distinguishing between carbon and nitrogen could be difficult only in the presence of (significantly) heavier atoms. In the case of binary C—N compounds, the difference in the scattering power of about 15% between these elements allowed differentiating them rather comfortably, as documented by hundreds of examples from organic chemistry.

The high‐pressure SC‐XRD data acquisition and analysis procedure were previously developed and described in detail in ref. [63]. Recently, this method was also successfully employed by other independent research groups.^[^ ^64^, ^65^, ^66^ ^]^ The powder X‐ray diffraction data was analyzed using Dioptas,^[^ ^67^ ^]^ and Le Bail analysis was performed with the FullProf software.^[^ ^68^ ^]^

### Raman Spectroscopy Measurements

Confocal Raman spectroscopy measurements were performed on three distinct setups. At the Commissariat à l’Énergie Atomique (CEA), an Alpha300M+ instrument (WITec) was employed with a continuous Ar–Kr laser using either the 488.0 or 647.1 nm lines with a focused laser spot of less than 1 µm. The Stokes Raman signal was collected in a back‐scattering geometry by a CCD coupled to an 1800 l mm^−1^ grating, allowing a spectral resolution of ≈1.5 cm^−1^. Automated motorized sample positioning with piezo‐driven scan stages of submicron accuracy allowed for precise Raman spectral imaging of the sample. At the BGI, a LabRam spectrometer equipped with a ×50 Olympus long working distance objective was employed. For the sample excitation, a continuous He–Ne laser (632.8 nm) with a focused laser spot of about 2 µm in diameter was used. The Stokes Raman signal was collected in a backscattering geometry by a CCD coupled to an 1800 l/mm grating, allowing a spectral resolution of ≈2 cm^−1^. With this setup, the Raman signal of TCNE was measured up to 116 GPa (Figure S21, Supporting Information) At the APS, the GSECARS Raman system was utilized, and an excitation wavelength of 532 nm was selected. The details of this setup were described elsewhere.^[^ ^69^ ^]^ Raman spectroscopy measurements were performed on all samples after their synthesis (see Figures S22–S24, Supporting Information).

### Scanning Electron Microscopy Measurements

The visualization of the indented diamond anvil of sample #4 was made with a ZEISS SEM, Leo Gemini 1530 with a Schottky field emission gun using secondary and backscattered electrons at 0.8 nA and 20 kV generated by the scanning electron microscope column.

### Transmission Electron Microscopy Measurements and Focused Ion Beam Preparation

A thin foil for transmission electron microscopy (TEM) investigations was made from the recovered sample #4 using a micro‐sampling technique in a dual beam‐focused ion beam (FIB) milling apparatus (FEI, Scios). The TEM‐thin sample with a size of 5 × 5 × 0.3 µm on the diamond anvil was milled with the bottom diamond anvil by Ga‐ions accelerated at 30 kV and a current of 5–30 nA in the FIB. The foil was eventually polished to be electron transparent with a low current of 100–300 pA at a low irradiation angle of up to 3 degrees. Then, it was cleaned with the probe current at 5 kV and 10 pA.

Conventional TEM observation with bright‐field (BF) and dark‐field (DF) TEM assisted with selected area electron diffraction (SAED) was performed with a scanning transmission electron microscope (FEI, Titan G2 80–200 S/TEM) equipped with an energy‐dispersive X‐ray spectrometer (EDS) and electron‐energy loss‐spectrometer (EELS), operated at 200 kV.

Carbon and nitrogen's K edge energy‐loss near edge structures (ELNES) were investigated with an EELS (Gatan GIF Quantum SE, post‐column energy filter system). The ELNES spectra were acquired with an incident beam with a convergent angle of 10 mrad and a correction semi‐angle of 9.2 mrad for 10 s with 0.1 s readout time multiplied by 100 times.

### Ab Initio Calculations

Kohn–Sham DFT‐based electronic structure calculations were performed with the QUANTUM ESPRESSO package^[^ ^70^, ^71^, ^72^ ^]^ using the projector augmented wave method.^[^ ^73^ ^]^ The generalized gradient approximation by Perdew–Burke–Ernzerhof (PBE) was used for exchange and correlation,^[^ ^74^ ^]^ with the corresponding potential files: For C and N the 1s electrons were treated as scalar‐relativistic core states. The authors included van der Waals corrections following the approach by Grimme et al. as implemented in Quantum Espresso.^[^ ^75^ ^]^ Convergence tests with a threshold of 1 meV per atom in energy and 1 meV Å per atom for forces led to a Monkhorst–Pack^[^ ^76^ ^]^ *k*‐point grid of 12 × 8 × 16 for *oP*8‐CN, 16 × 16 × 16 for *tI*24‐CN_2_, 16 × 16 × 8 for *tI*14‐C_3_N_4_, and 2 × 2 × 16 for *hP*126‐C_3_N_4_ with a cutoff for the wave‐function expansion of 80 Ry for all phases.

Variable cell relaxations (lattice parameters and atomic positions) were performed on all experimental structures to optimize the atomic coordinates and the cell vectors until the total forces were smaller than 10^−4^ eV Å^−1^ per atom and the deviation from the experimental pressure was below 0.1 GPa.

Equation of state (EOS) calculations were performed via variable‐cell structural relaxations in 10 GPa steps up to 100 GPa for *oP*8‐CN and 150 GPa for the other phases. The authors fitted a third‐order Birch–Murnaghan EOS to the energy‐volume points, calculated the P(V) , and benchmarked versus the target pressure of the relaxations to ensure convergence.

Phonons for *hP*126‐C_3_N_4_ were calculated using the temperature‐dependent effective potential method (TDEP^[^ ^77^, ^78^ ^]^) at the level of harmonic approximation for TDEP Hamiltonian including quantum and thermal effects at 300 K in a 1 × 1 × 3 supercell with respectively adjusted *k*‐points at ambient and synthesis pressure (125 GPa) using the cell relaxed to ambient conditions. Phonon dispersion relations were calculated with Phonopy^[^ ^79^ ^]^ in a 3 × 2 × 4, 3 × 3 × 3, and 3 × 3 × 2 supercells for *oP*8‐CN, *tI*24‐CN_2_, and *tI*14‐C_3_N, respectively. *k*‐points have been adjusted according to the supercell size.

To estimate the hardness of the four carbon nitrides, two phenomenological models were used: A microscopic one developed by Lyakhov and Oganov et al.^[^ ^80^ ^]^ and a so‐called macroscopic model introduced by Chen et al.^[^ ^81^ ^]^ For the microscopic model, the chemical valences of C and N atoms and the covalent radii values were taken from those defined in the USPEX code.^[^ ^82^ ^]^ For the macroscopic model, the polycrystalline bulk (*K*) and shear (*G*) moduli were calculated (Table S17, Supporting Information). To obtain these, the anisotropic single‐crystal elastic stiffness constant C*_ij_* (Table S18, Supporting Information) was calculated and the Hill approximation was applied. The energy‐strain relationships and a finite difference method with (+/−) 1 and 2% strain were used, where the total energies were calculated by Quantum Espresso with the numerical parameters given above. Furthermore, the directional dependence of Young's modulus (Figure S25, Supporting Information) was calculated. Fracture toughness (Table S14, Supporting Information) was estimated using the approach from Mazhnik et al.^[^ ^83^ ^]^

Electronic structure calculations were performed with the generalized gradient approximation by PBE for exchange and correlation. Additional calculations for the band gaps and band structures were performed with the Heyd–Scuseria–Ernzerhof (HSE) hybrid functional.^[^ ^84^ ^]^ The standard screening parameters were employed along with a *k*‐point (q‐grid) of 12 × 8 × 16 (4 × 4 × 4) for *oP*8‐CN, 8 × 8 × 8 (2 × 2 × 2) for *tI*24‐CN_2_, 12 × 12 × 12 (4 × 4 × 4) for *tI*14‐C_3_N_4_, and 2 × 2 × 8 (2 × 2 × 2) for *hP*126‐C_3_N_4_, with the *k*‐points defining the band gaps, and with PBE being employed in the HSE sampling. The character of the gap does not change going from PBE to HSE, the size increases in general by ≈1.5–2 eV (see Table S19 and Figures S14–S17, Supporting Information).

The energy released from the reaction C*_x_* N*_y_* → *x*·C + *y*/2·N_2_ at 300 K and *P* = 0 GPa for all four compounds was estimated from the calculated harmonic phonon dispersions (see Table S15). The enthalpy and entropy of N_2_ were derived from thermochemical tables,^[^ ^85^ ^]^ as suggested by Zhang et al.^[^ ^86^ ^]^ Carbon was assumed in the graphite phase at ambient conditions.

The piezoelectric coefficients for the two non‐centrosymmetric C—N phases (i.e.*, tI*14‐C_3_N_4_ and *tI*24‐CN_2_, see Table S16) were computed using the ab initio density‐functional perturbation theory (DFPT)^[^ ^87^ ^]^ as implemented in the Vienna Ab Initio Simulation Package (VASP).^[^ ^88^, ^89^, ^90^ ^]^ The computational parameters in VASP matched those used in QE.

### Electron–Electron Correlation Effects

The degree of the electron‐electron correlation effects in *tI*14‐C_3_N_4_ had been elucidated at the level of the Hubbard model by comparing the strength of the effective Coulomb interaction *U* with the relevant bandwidth *W*. The authors had considered the separated valence bands in the region from −3 to 0 eV obtained using PBE approximation at ambient conditions (see Figure S18, Supporting Information), and constructed the set of Wannier orbitals from the corresponding Bloch functions utilizing the procedure of maximal localization.^[^ ^91^, ^92^ ^]^ The resulting Wannier functions (three per formula unit) were centered between the neighboring N atoms and were all equivalent. The screening effects were calculated within the constrained random phase approximation (cRPA) scheme.^[^ ^93^ ^]^

Further details of the crystal structure investigation(s) may be obtained from the Fachinformationszentrum Karlsruhe, 76344 Eggenstein‐Leopoldshafen (Germany), quoting the depository number CSD‐2202353‐2202360, 2260017, and 2260018.

## Conflict of Interest

The authors declare no conflict of interest.

## Supporting information

Supporting Information

Supporting Information

## Data Availability

Structural data was deposited at the Cambridge Crystallographic Data Centre (CCDC), CSD 2202353‐2202360, 2260017, and 2260018. All other datasets generated during and/or analyzed during the current study are available from the corresponding author upon reasonable request.
