# Supplementary material for: Synthesis of Ultra‐Incompressible and Recoverable Carbon Nitrides Featuring CN4 Tetrahedra
Source: Adv Mater. 2023 Dec 10;36(3):2308030. doi: 10.1002/adma.202308030 (PMC11475261; doi:10.1002/adma.202308030)
Supplement: Supplementary file 1 — Supporting Information [file ADMA-36-2308030-s001.pdf]

ADVANCED MATERIALS

Supporting Information

for *Adv. Mater.*, DOI 10.1002/adma.202308030

Synthesis of Ultra-Incompressible and Recoverable Carbon Nitrides Featuring CN_4 Tetrahedra

Dominique Laniel, Florian Trybel, Andrey Aslandukov, Saiana Khandarkhaeva, Timofey Fedotenko, Yuqing Yin, Nobuyoshi Miyajima, Ferenc Tasnádi, Alena V. Ponomareva, Nityasagar Jena, Fariia Iasmin Akbar, Bjoern Winkler, Adrien Néri, Stella Chariton, Vitali Prakapenka, Victor Milman, Wolfgang Schnick, Alexander N. Rudenko, Mikhail I. Katsnelson, Igor A. Abrikosov, Leonid Dubrovinsky and Natalia Dubrovinskaia*

Supporting Information

Synthesis of Ultra-Incompressible and Recoverable Carbon Nitrides Featuring CN_4 Tetrahedra

Dominique Laniel, Florian Trybel, Andrey Aslandukov, Saiana Khandarkhaeva, Timofey Fedotenko, Yuqing Yin, Nobuyoshi Miyajima, Ferenc Tasnádi, Alena V. Ponomareva, Nityasagar Jena, Fariia Iasmin Akbar, Bjoern Winkler, Adrien Néri, Stella Chariton, Vitali Prakapenka, Victor Milman, Wolfgang Schnick, Alexander N. Rudenko, Mikhail I. Katsnelson, Igor A. Abrikosov, Leonid Dubrovinsky, Natalia Dubrovinskaia*

Supporting Text

Crystallographic Description of the four carbon nitrides

The structure of $tI14$-C_3N_4 has a tetragonal unit cell (space group I-42m, #121, see Table S1) with parameters a = 3.2285(6) Å and c = 6.4526(16) Å (V = 67.26(3) Å^3). It is composed of three crystallographically distinct atoms, C1, C2, and N1, on the 2a, 4d, and 8i Wyckoff sites, respectively. Both carbon atoms are fourfold coordinated by nitrogen atoms, forming the long-sought-after CN_4 tetrahedron, and the N1 atom is threefold coordinated by carbon atoms (Figure 3a). The C1-N1 and C2-N1 bonds are very similar in length, being respectively of 1.381(2) Å and 1.399(2) Å. The average N-C-N bond angle is 109.5(1)°, perfectly matching the value expected of an ideal tetrahedron, highlighting the sp^3-hybridization of the carbon atoms. The average C-N-C bond angle being of 110.0(1)° also suggests the sp^3-hybridization of the nitrogen atoms. Corner-sharing CN_4 tetrahedra form a three-dimensional network, which is easy to visualise (see Figure 3b,c), if the structure of $tI14$-C_3N_4 is considered as originating from the sphalerite (ZnS) structure type—which is that of cubic boron nitride as well. It consists of layers of tetrahedra stacked in an ABC sequence (Figure 3b) in the $[1\bar{1}2]$ direction, but instead of complete layers as in ZnS, the layers in $tI14$-C_3N_4 are incomplete (Figure 3c): each fourth tetrahedron in a row is missing. $tI14$-C_3N_4 is isostructural to β-Cu_2HgI_4.[1]

Some single-crystal datasets of $tI14$-C_3N_4 revealed supercell reflections. The largest superstructure that could be identified has an orthorhombic unit cell (space group Pma2, #28) with lattice parameters a = 13.6827(14) Å, b = 13.6728(11) Å and c = 19.346(4) Å (V = 3619.2(9) Å^3) at 130 GPa, as seen in Figure S26 and Figure S27. An experimental crystal model for this supercell could not be obtained due to an insufficient number of reflections, but a theoretical model could be produced. As expected, this model exhibits a similar atomic arrangement as described above, but with very minor displacements (< 0.001 Å) of the atoms from their higher symmetry positions.

The structure of $hP126$-C_3N_4 has a large hexagonal unit cell (space group $P6_3/m$, #176, see Table S3) with lattice parameters of a = 17.690(8) Å and c = 2.2645(11) Å (V = 613.7(5) Å^3) at 124 GPa (Figure 3c). It is composed of 21 crystallographically distinct atoms, nine C and twelve N, all on the 6h Wyckoff site. Like in $tI14$-C_3N_4, carbon and nitrogen atoms in $hP126$-C_3N_4 are fully saturated and tetrahedrally and triply coordinated by N and C atoms, respectively. A polyhedral model of $hP126$-C_3N_4 viewed along the c direction is shown in Figure 3d. The corner-sharing CN_4 tetrahedra form a very complex arrangement. Some features of this structure are reminiscent of β-Si_3N_4,[2] namely the six-membered rings of tetrahedra (*sechser*-rings, according to F. Liebau's[3] nomenclature), which form large empty channels in the c-direction centred at the origin of the unit cell. However, unlike β-Si_3N_4, which is exclusively composed of interlinked *sechser*-rings, $hP126$-C_3N_4 is made up of interlinked three- (*dreier*-rings), four- (*vierer*-rings), and five-membered rings (*fünfer*-rings).

The average C-N bond length was determined to be 1.376(5) Å, which is, within uncertainty, identical to the C1-N bond in $tI14$-C_3N_4. The average N-C-N bond angle is 109.43(7)°—analogous to that in $tI14$-C_3N_4, pointing to the sp^3-hybridization of carbon. The C-N-C bond angle varies between 108.31(2) and 124.5(4)° (with one outlier of 132.6(3)°), the average bond angle being 117.5(1)°, which suggests predominantly sp^2-hybridized nitrogen atoms with a minor fraction being sp^3-hybridized, in contrast to fully sp^3-hybridized N in $tI14$-

C_3N_4. Interestingly, three carbon and three nitrogen atoms laying in the same *ab* plane form flat C_3N_3 rings, reminiscent of triazine rings in heptazine and its derivatives (highlighted by red circles in Figure 3e), albeit solely composed of single bonds. The structure of *oP*8-CN has an orthorhombic unit cell (space group *Pnnm*, #58) with a = 4.892(4) Å, b = 3.7841(12) Å and c = 2.2880(9) Å (V = 42.36(4) Å^3). The quality of single-crystal XRD data was sufficient for the structure solution and refinement (Table S5). The compound was found to have the CN stoichiometry and the β-InS structure type.[4] Its structure is composed of two crystallographically distinct atoms, C1, and N1, both on the 4*g* Wyckoff site (thus its Pearson index is *oP*8-CN). The C1 atoms are fourfold coordinated by three nitrogen atoms and one carbon atom, producing C-CN_3 tetrahedra, while the N1 atoms are threefold coordinated by C1 atoms. The structure can be easily visualised in a polyhedral model (Figure 4a). It consists of corrugated layers of C-CN_3 tetrahedra sharing N vertices and laying in the *bc* plane. Carbon apexes of the tetrahedra point towards neighbouring layers connected through triply coordinated nitrogen atoms, thus forming in the *bc* plane a corrugated honeycomb-like net of the 1:1 C:N composition (Figure 4b). The C1-N1 bond length is 1.418(5) Å—slightly shorter than typical C-N single bonds at ambient conditions (~1.47 Å)[5–7]—and the C1-C1 contact is 1.485(8) Å, the latter similar to the C-C bond in diamond at the same pressure.[8] The average N/C-C-N and C-N-C bond angles are 109.43(9)° and 117.63(4)°, suggesting sp^3- and sp^2-hybridization for C and N atoms, respectively. Such a CN compound with the β-InS structure type was previously reported to be formed by laser-heating of graphite in molecular nitrogen at pressures above 55 GPa and 7000 K,[9] but its structure could not be refined based on that time only available powder diffraction data.

One more hitherto unknown C-N phase, *tI*24-CN_2, was synthesised in the DAC containing phosphorus and nitrogen (sample #3) after its laser-heating to ~2500 K at 134 GPa due to a reaction of nitrogen with carbon from the diamond anvils. A number of other phases were also observed in this DAC, namely the well-known cg-N and bp-N polymeric nitrogen allotropes,[10,11] the incommensurate phase IV of phosphorus,[12] and phosphorus nitride PN_2.[13] While the powder X-ray diffraction pattern is very complex, as shown in a Le Bail refinement (Figure 2), all phases could be identified by single-crystal X-ray diffraction and the structure of *tI*24-CN_2 solved and refined (Table S6).

The structure of *tI*24-CN_2 has a tetragonal unit cell ($I\bar{4}2d$ space group, #122) with the lattice parameters a = 5.9864(13) Å and c = 3.230(2) Å (V = 115.75(8) Å^3) at 134 GPa. The carbon and nitrogen atoms in *tI*24-CN_2 are, respectively, fourfold and threefold coordinated, but not all covalent bonds are heteroatomic. Indeed, while carbon is solely making C-N bonds—forming a CN_4 tetrahedron—nitrogen also has a N-N bond (Figure 4c,d). Thus, the structure of *tI*24-CN_2 can be presented as a framework of CN_4 tetrahedra connected through N_2 dimers. When looking along the *c*-axis (Figure 4c), the crystal structure can be described as four-rings interconnected through their corners and N_2 dimers, and repeating in the [001] direction, forming square-shaped channels along this direction. The four C-N bonds of the CN_4 tetrahedra are identical within their uncertainty, with two of 1.379(7) Å and two others of 1.389(7) Å; bond lengths very similar to those of *oP*8-CN, *tI*14-C_3N_4 and *hP*126-C_3N_4. The N-C-N bond angles of the CN_4 tetrahedra vary between 104.0(3)° and 116.7(6)°, with the average value being 109.5(3)°—in line with sp^3-hybridization in ideal CN_4 tetrahedra. The N-N bond length is 1.272(11) Å; slightly shorter than what is expected from a single bond in 3D nitrogen polymeric

solids, found to have a length of 1.3410(15) Å in cg-N (122 GPa)[14] and of 1.338(6) Å and 1.435(7) Å in bp-N (140 GPa)[11]. Still at 134 GPa, *tI*24-CN_2 has an average angle formed by the C/N-N-C bonds of 117.3(4)°, suggesting predominantly sp^2-hybridization of nitrogen.

Raman spectroscopy measurements

Raman spectroscopy measurements were performed on all three samples along with reference Raman spectra of TCNE up to 116 GPa (Figure S21). From sample #2 (Figure S22) at 72 GPa, four new modes are observed, at frequencies of 415, 734, 768, and 1295 cm^{-1}, matching the modes previously experimentally observed and calculated for *oP*8-CN.[9] A representative spectrum collected at 124 GPa from sample #1—containing N_2, *tI*14-C_3N_4, and *hP*126-C_3N_4—is shown in Figure S23. Obtained using the utilities provided on the Bilbao Crystallographic Server (https://www.cryst.ehu.es/), the *tI*14-C_3N_4 phase is expected to have twelve Raman-active modes ($\Gamma = 2A_1 + 2B_1 + 3B_2 + 5E$) while *hP*126-$C_3N_4$ has 168 ($\Gamma = 42A_g + 42^2E_{2g} + 21^1E_{1g} + 42^1E_{2g} + 21^2E_{1g}$) Raman active modes. The extremely large number of possible Raman modes from the C_3N_4 polymorphs combined with the fact that these two phases represent a very small volume fraction of the sample chamber—with molecular nitrogen being by far the most prominent solid—prevents a reliable analysis of the Raman spectra. For this reason, only a single Raman mode could be clearly identified as belonging to either *tI*14-C_3N_4 or *hP*126-C_3N_4. A Raman spectrum collected on sample #3 at 134 GPa is shown in Figure S24, along with the calculated Raman spectra of *tI*24-CN_2, cg-N,[10] and bp-N.[11,15] An experimentally measured mode at 792 cm^{-1} is found to match the most intense mode calculated for *tI*24-CN_2, along with a broad one at 530 cm^{-1} that is also attributed to *tI*24-CN_2.

Description of the bond lengths, bond order and interatomic angles of the recovered carbon nitrides

Having direct consequences on the properties of the carbon nitrides, the analysis of the interatomic distances, bond order and bond angles in these recovered compounds is of the utmost importance and is summarized in Table S7. As expected, the bond lengths measured at 1 bar are all longer than at the synthesis pressure of these solids. In *oP*8-CN, *tI*14-C_3N_4, *hP*126-C_3N_4, and *tI*24-CN_2, the average C-N contact at 1 bar is 1.452(2), 1.470(2), 1.461(5), and 1.477(9) Å, respectively. This is in perfect agreement with known C-N single bonds at ambient conditions (~1.47 Å)[5–7] and represents a 2.4, 5.8, 6.2, and 6.7% increase compared to the bond lengths at the respective synthesis pressure of *oP*8-CN (72 GPa), *tI*14-C_3N_4 (124 GPa), *hP*126-C_3N_4 (124 GPa) and *tI*24-CN_2 (134 GPa). For *oP*8-CN, the C-C bond length at 1 bar is 1.569(8) Å (5.7% increase from 72 GPa), mildly longer than in diamond (1.54461(3) Å).[16] The N-N bond in the *tI*24-CN_2 compound is 1.359(12) Å, which is a bit shorter than expected for a single bond, but still closer to the latter than to a double bond.[5] This higher electronic density between the nitrogen atoms leads to an N-N greater bond strength—helping the *tI*24-CN_2 solid to be stable at ambient conditions—and a lower electronic density for the C-N bonds, providing a possible reason why they are slightly longer in *tI*24-CN_2 compared to the other three carbon nitrides.

The bond order in all four carbon nitrides at ambient conditions was further explored by calculating the integrated crystal orbital bond index (ICOBI)—essentially a measure of the bond order[17]—using the LOBSTER software.[17,18] The results, shown in Table S7, are in very good agreement with the bond order inferred from the bond lengths described above. Indeed, in the *oP*8-CN, *tI*14-C_3N_4, *hP*126-C_3N_4 and *tI*24-CN_2 compounds, the C-N ICOBI values are 0.96,

0.95, 0.95 and 0.94, which indicates the fully covalent nature of the bonds and a bond order of one. Likewise, in *tI*24-CN_2, the N-N bond has an ICOBI of 1.04, corresponding to a single bond. In *oP*8-CN, an ICOBI value of 0.83 was obtained for the C-C bond, suggesting slightly less electronic density than usual for a single bond. This can be rationalized in the following way: nitrogen, being significantly more electronegative than carbon, pulls towards itself more electronic density, leaving less for the C-C bond.

Remarkably, the bond angles within the C-CN_3 and CN_4 tetrahedra are essentially unchanged between the synthesis pressure of the *oP*8-CN, *tI*14-C_3N_4, *hP*126-C_3N_4, and *tI*24-CN_2 solids and ambient pressure, as seen in Table S7. Indeed, in all four compounds, the N/C-C-N bond angles are, within uncertainty, still equal to the ideal tetrahedron angle of 109.5°—showing the extreme resilience of these tetrahedra against pressure. Moreover, the interconnection of tetrahedra also appears barely affected by the decompression, as the N/C-N-C bond angle only increases by 0.99(7)° (118.62(3)° at 1 bar), 1.9(2)° (111.9(1)° at 1 bar), 0.8(2)° (118.3(1)° at 1 bar) and 0.6(8)° (117.9(4)° at 1 bar) for *oP*8-CN, *tI*14-C_3N_4, *hP*126-C_3N_4 and *tI*24-CN_2, respectively, between the synthesis pressure and ambient pressure. As such, the orbital hybridization at 1 bar is the same as at higher pressures, with carbon preserving its sp^3-hybridization in all compounds while nitrogen says mainly sp^2-hybridized in all but *tI*14-C_3N_4, in which it is sp^3-hybridized.

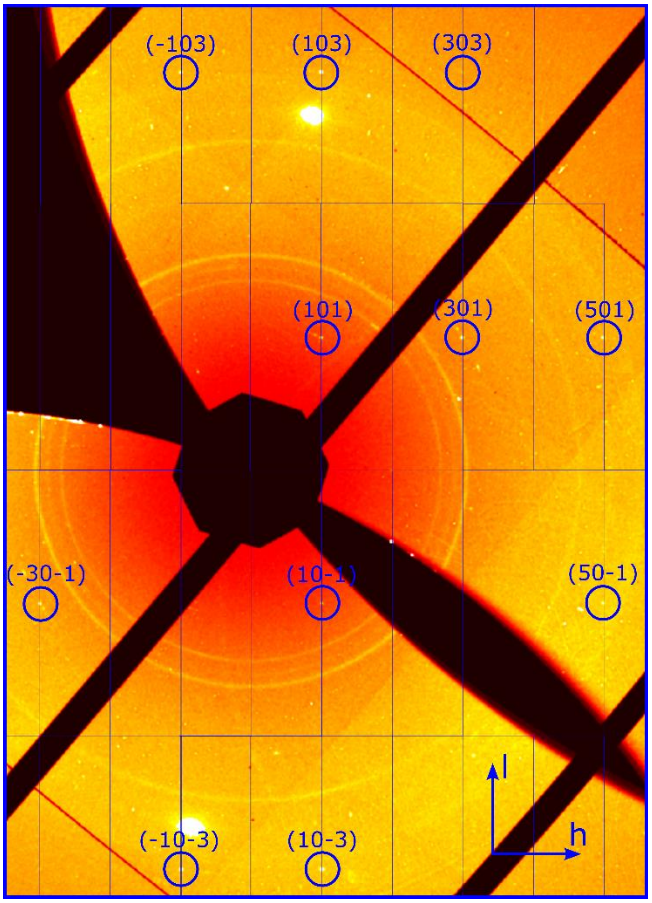

Figure S1: Slice of the (h0l) reciprocal space of a *oP*8-CN single crystal at 72 GPa.

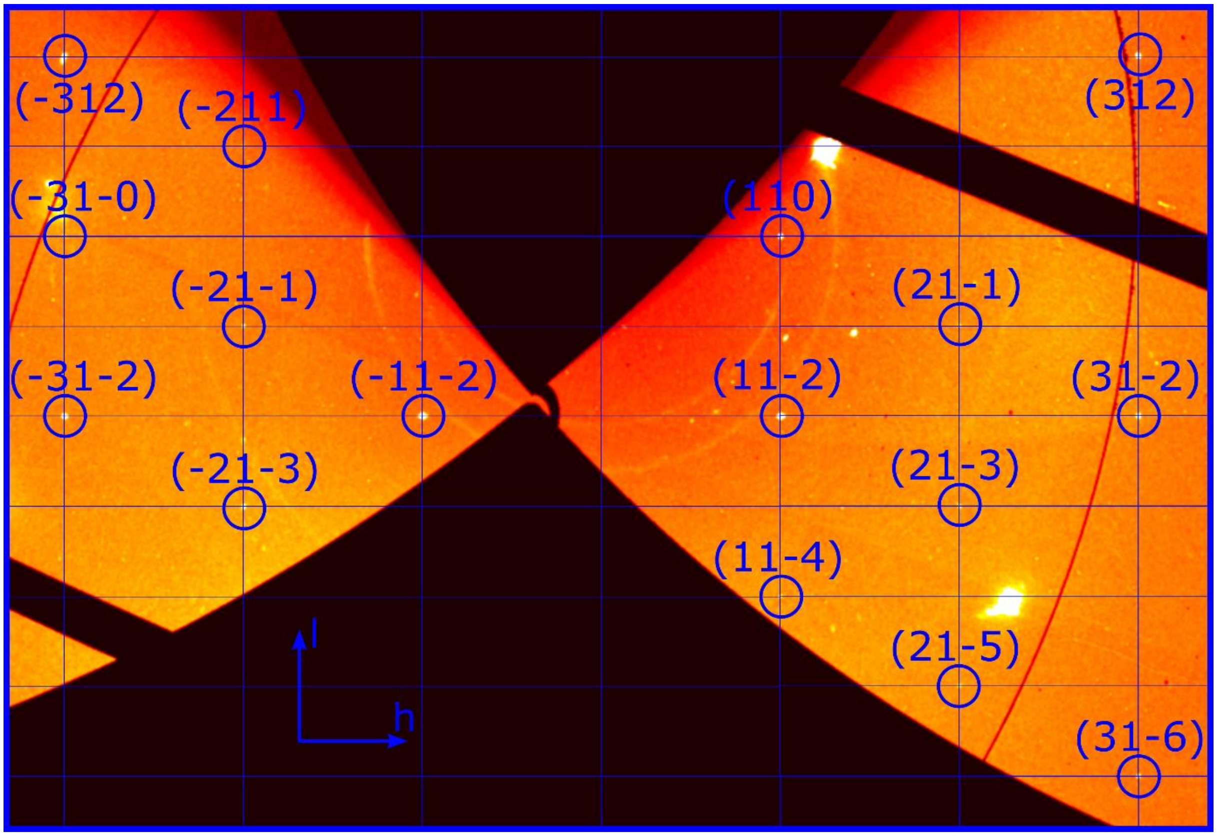

Figure S2: Slice of the (h1l) reciprocal space of a *tI*14-C_3N_4 single crystal at 124 GPa.

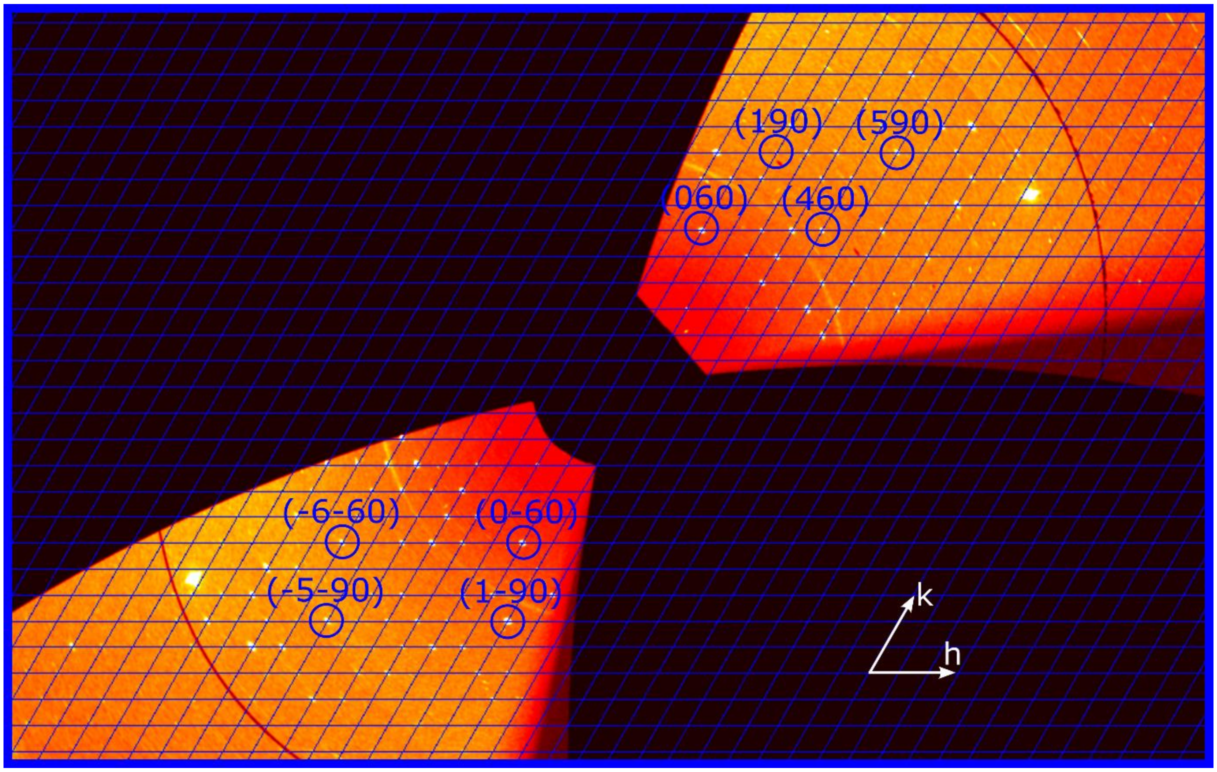

Figure S3: Slice of the (hk0) reciprocal space of a *hP*126-C_3N_4 single crystal at 124 GPa. For clarity only a few reflections are notated with their corresponding (hkl) index.

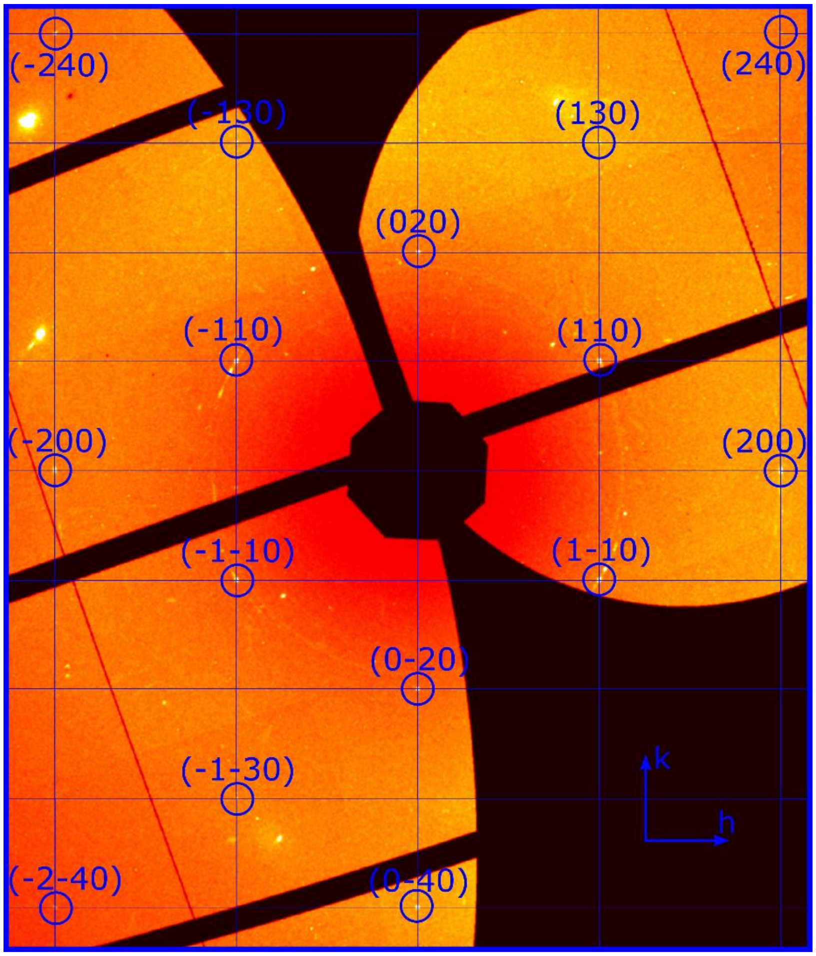

Figure S4: Slice of the (hk0) reciprocal space of a *tI*24-CN_2 single crystal at 137 GPa.

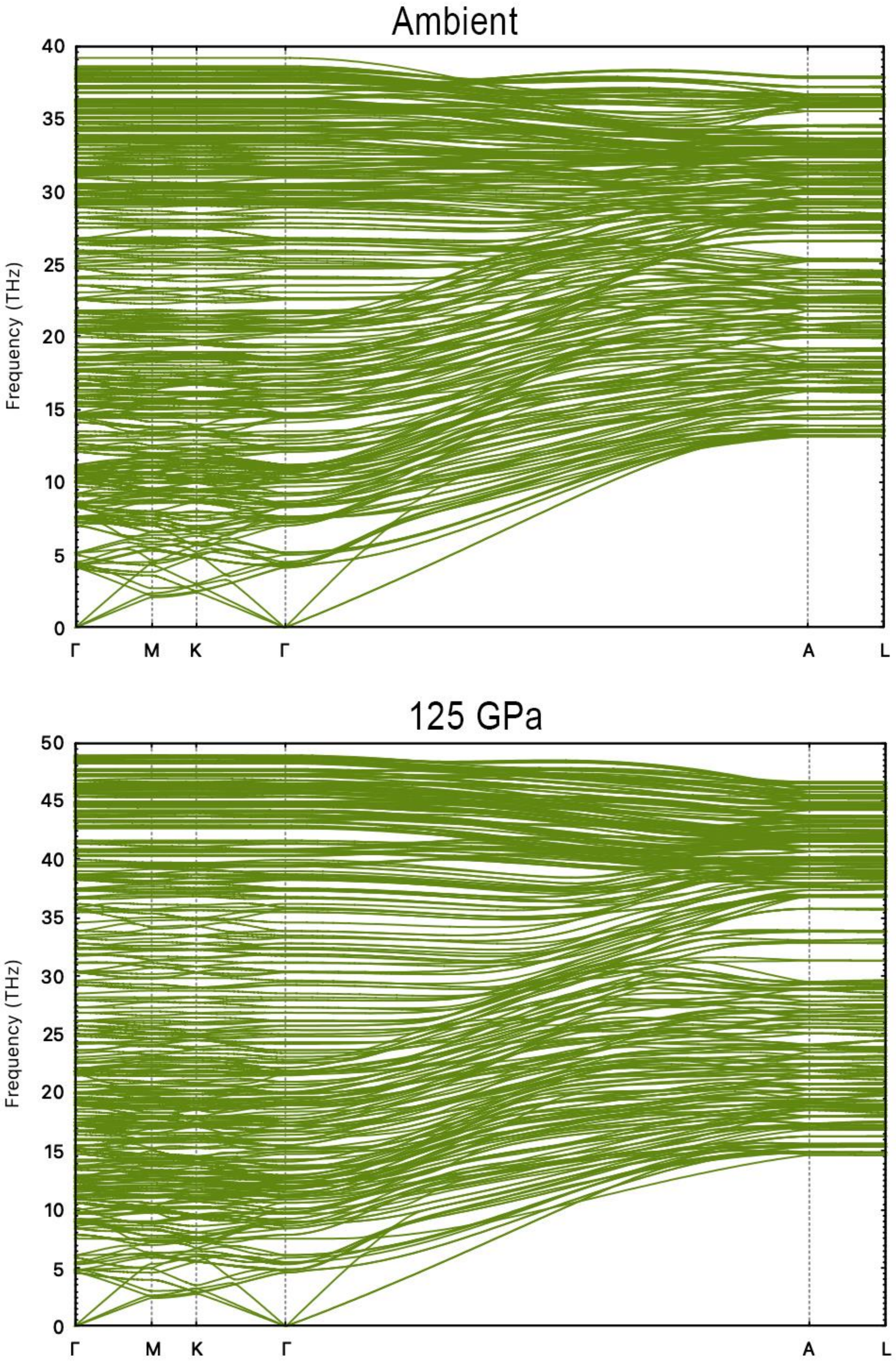

Figure S5: Phonon dispersion relation for $hP126$-C_3N_4 at ambient pressure and 125 GPa calculated at 300 K with TDEP[19] method.

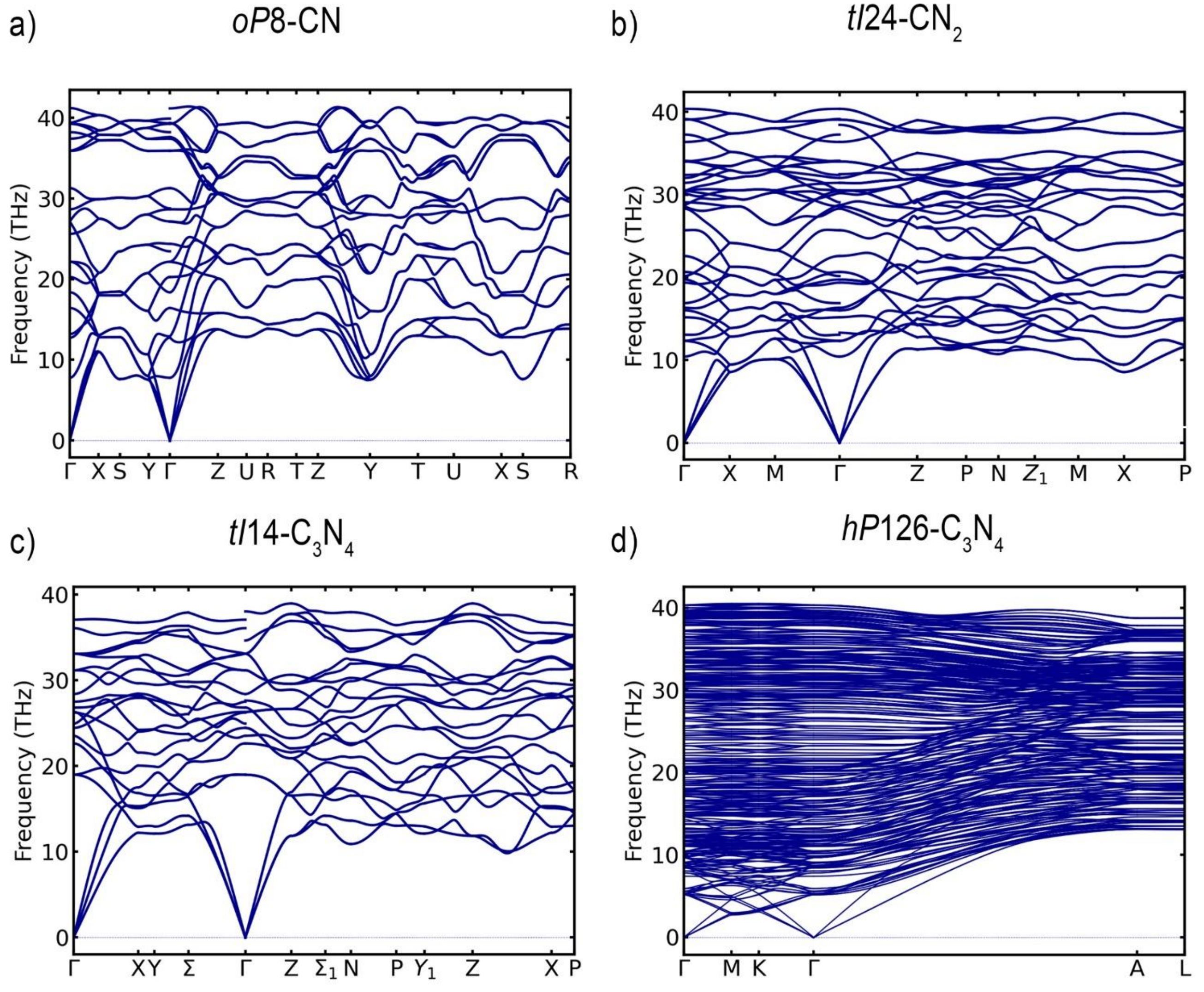

Figure S6: Phonon dispersion relation for a) *oP*8-CN, b) *tI*24-CN_2, c) *tI*14-C_3N_4 and d) *hP*126-C_3N_4 at ambient pressure, calculated with Phonopy.[20]

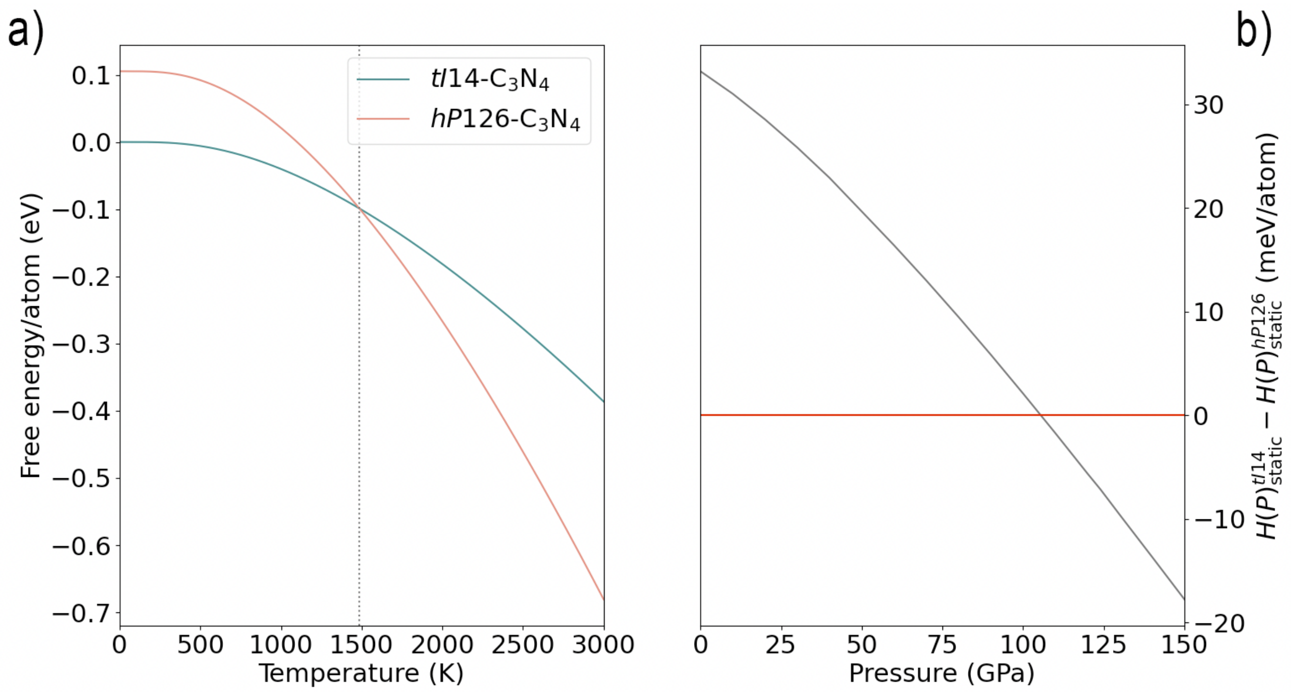

Figure S7: a) Gibbs free energy per atom of *tI*14-C_3N_4 and *hP*126-C_3N_4 at 124 GPa in quasi-harmonic approximation as a function of temperature. *hP*126-C_3N_4 has a lower energy above 1486 K. The reference energy is that of *tI*14-C_3N_4 at 0 K and 124 GPa. b) Static enthalpy difference per atom between *tI*14-C_3N_4 and *hP*126-C_3N_4 between 0 and 150 GPa. Above about 106 GPa, *tI*14-C_3N_4 has a lower enthalpy than *hP*126-C_3N_4, the latter becoming favourable at lower pressures.

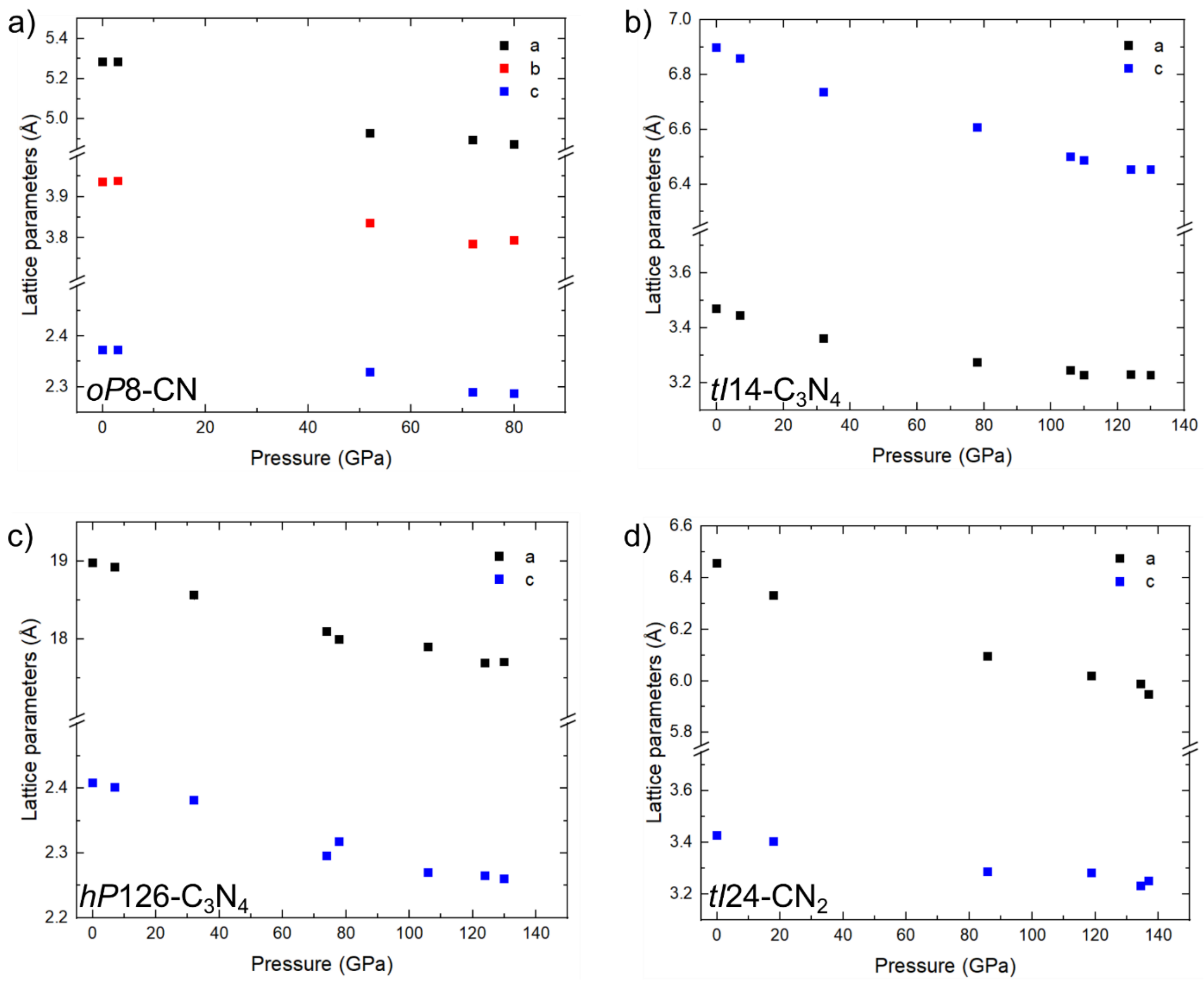

Figure S8: Lattice parameters as a function of pressure for *oP*8-CN (a), *tI*14-C_3N_4 (b), *hP*126-C_3N_4 (c) and *tI*24-CN_2 (d). Opposite to the results presented in ref. [9], no negative compressibility of the *b* lattice parameter of *oP*8-CN was observed.

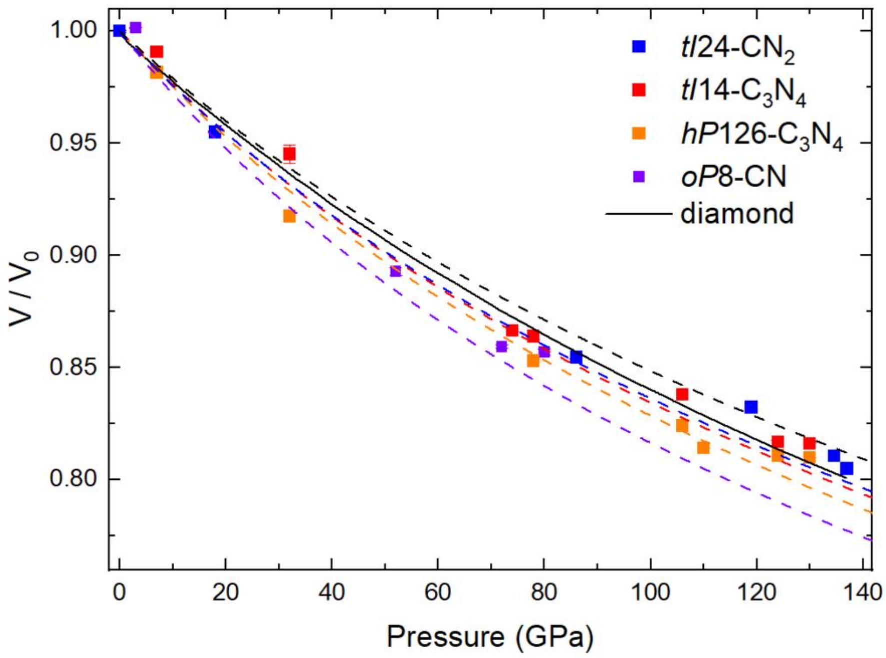

Figure S9: Experimental and calculated pressure dependences of the unit cell volume, normalized to the volume at 1 bar, for *oP*8-CN, *tI*14-C_3N_4, *hP*126-C_3N_4, and *tI*24-CN_2, as well as for diamond. The square symbols represent experimental data points obtained from SC-XRD data while the dashed lines are a fit of the DFT-calculations data with a third-order Birch-Murnaghan equation of state (Table S14). The full black line is the fit of the experimental data on diamond[8] using a third-order Birch-Murnaghan equation of state.

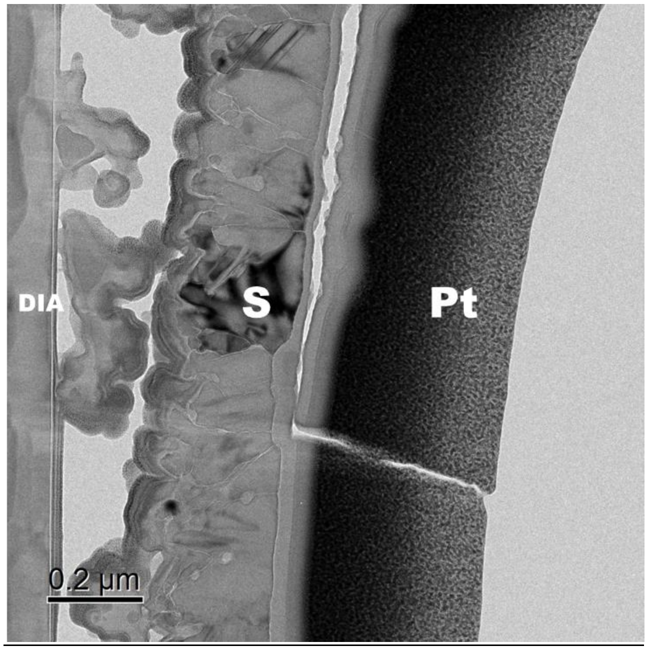

Figure S10: Bright-field transmission electron microscopy image of the cross-section of the recovered sample #1 (S) seen between the layer of diamond (DIA) from the anvil and the Pt deposition layer (Pt) from the focused ion beam.

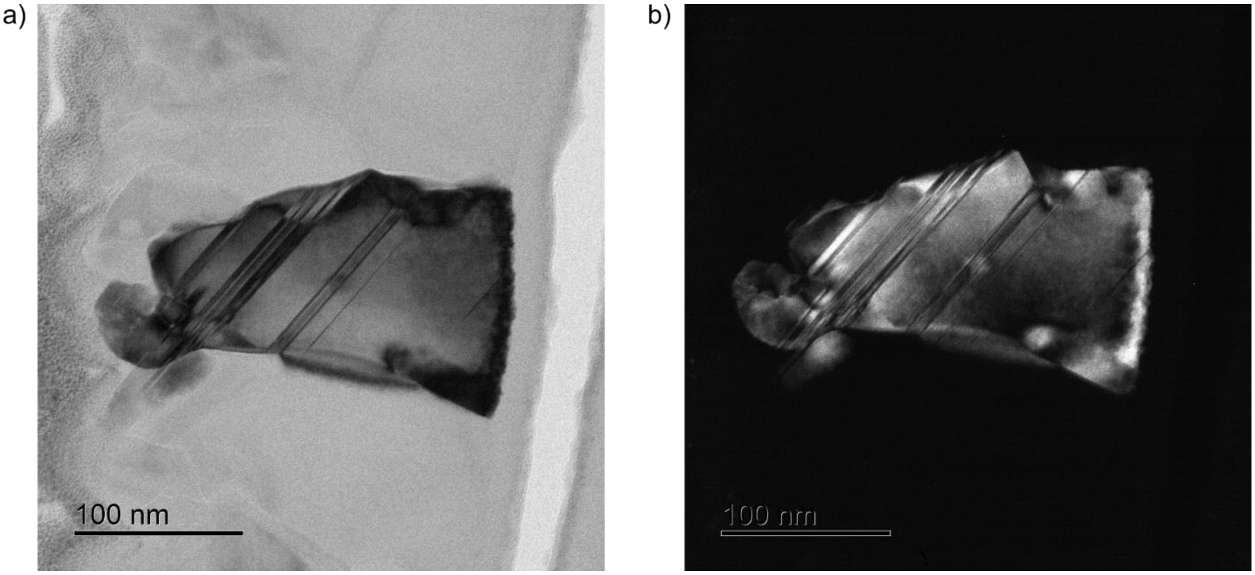

Figure S11: Transmission electron microscopy (TEM) images of a grain of *tI*14-C_3N_4. (a) Bright field TEM image displaying stacking faults and {112} twins. (b) Dark field TEM image. The diffraction vector is **g** = 020 of the primary domain.

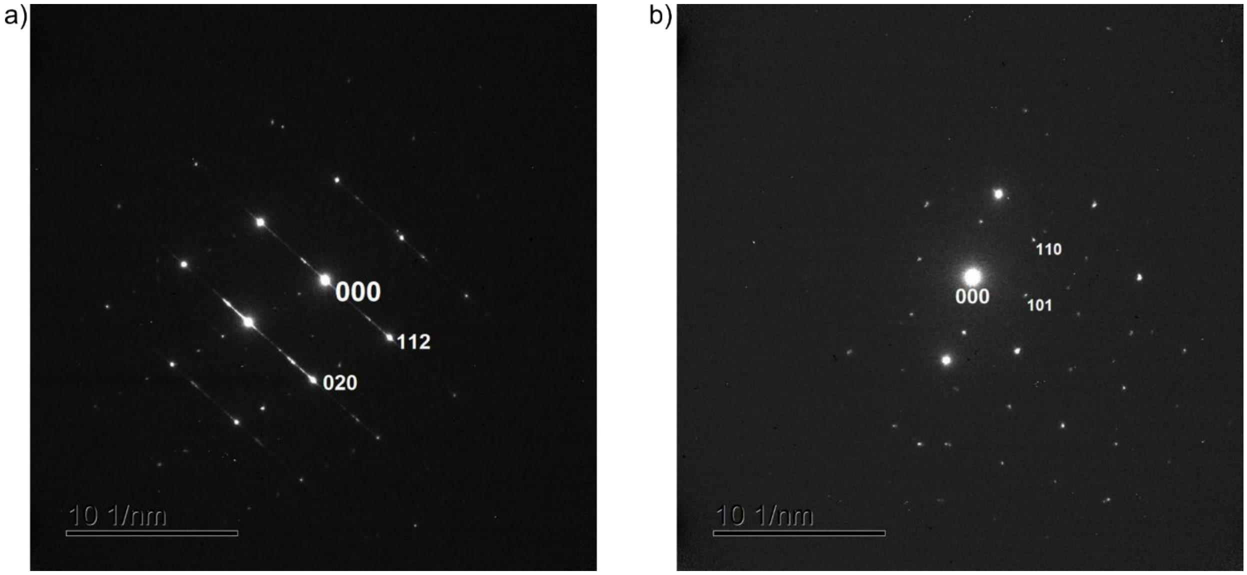

Figure S12: Selected area electron diffraction (SAED) patterns of the twinned grain of *tI*14-C_3N_4 imaged in Figure S11. (a) The twin plane is confirmed as the (112) plane. (b) The <111> zone axis SAED pattern. The indexing was performed for the *tI*14-C_3N_4 crystal with the unit cell parameters a = 3.47 Å and c = 6.90 Å in accordance with the single-crystal X-ray diffraction data (see Table S10). Several diffraction spots from other grains illuminated by the electron beam are also visible.

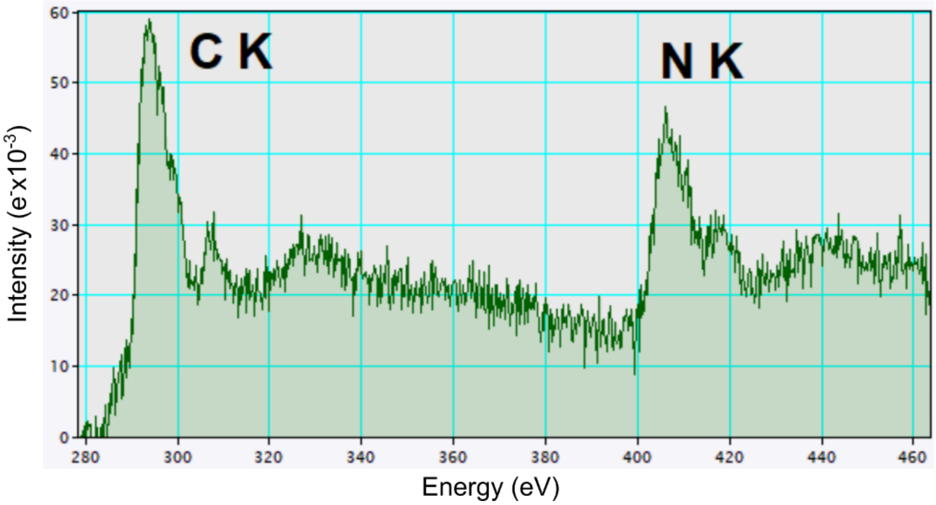

Figure S13: Carbon and nitrogen-K edge energy-loss near-edge structures of *tI*14-C_3N_4 measured from electron energy loss spectroscopy (EELS). Absence of π^* peaks suggest the sp^3-hybridized character of both the C and N atoms, in accordance with the conclusions reached from SC-XRD data. Quantitative analysis of the intensities is consistent with a 3:4 ratio between carbon and nitrogen, corresponding to the expected stoichiometry of the *tI*14-C_3N_4 compound.

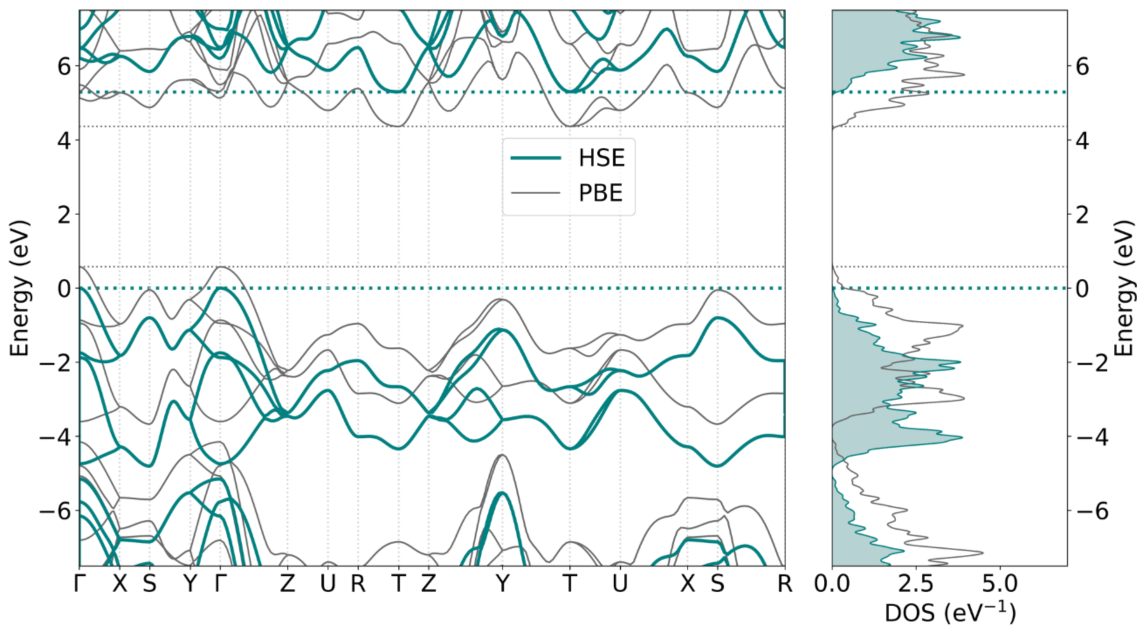

Figure S14: Electronic band structures and density of states using PBE (grey) and HSE (teal) at ambient pressure-temperature conditions for *oP*8-CN shifted with respect to the highest occupied state obtained with HSE. The dotted horizontal lines indicate the highest occupied and lowest unoccupied state.

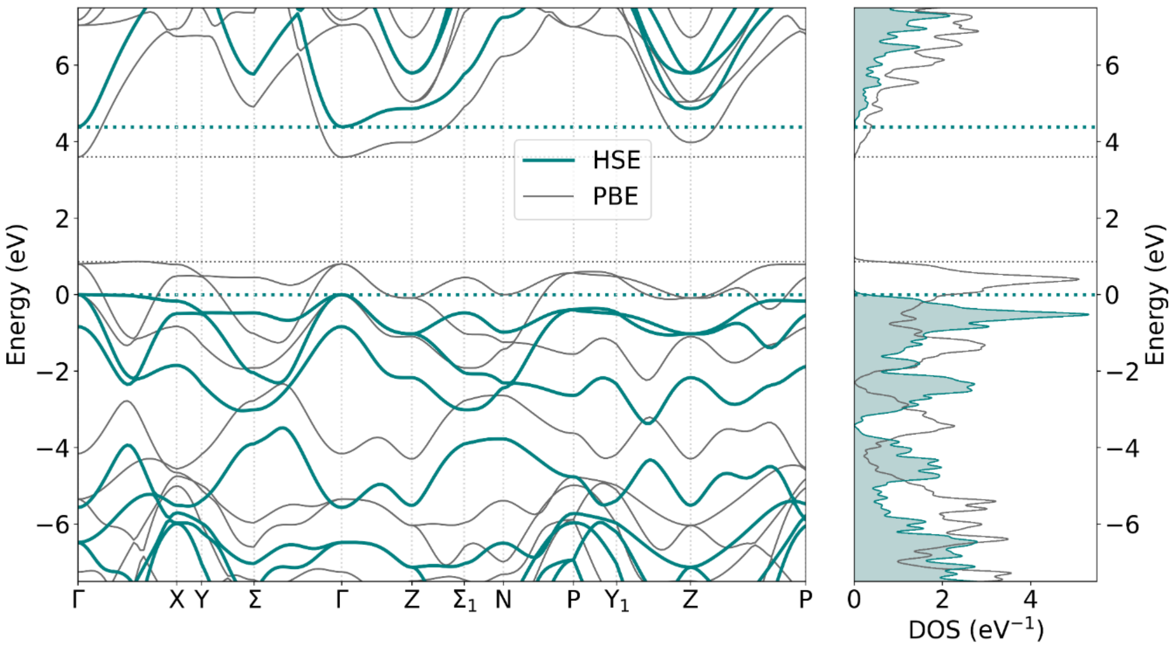

Figure S15: Electronic band structures and density of states using PBE (grey) and HSE (teal) at ambient pressure-temperature conditions for *tI*14-C_3N_4 shifted with respect to the highest occupied state obtained with HSE. The dotted horizontal lines indicate the highest occupied and lowest unoccupied state. From the electronic structure point of view, *tI*14-C_3N_4 appears to be a very interesting phase, because it exhibits flat regions in the valence band, giving rise to an enhanced van Hove singularity in the density of states located in the immediate vicinity of the band edge. The presence of such a feature at the Fermi energy, at the corresponding doping level, indicates a universal instability in *p*-doped *tI*14-C_3N_4 with respect to the formation of exotic states of matter, such as, for instance, charge density waves, itinerant magnetism, etc., as was demonstrated, for example, for the case of single-layered hole-doped InSe.[21,22] Note that within the many-body theory flat band can enhance correlation effects (Figure S18) and result in instabilities also when the flat band is not too close to the Fermi energy, due to virtual transitions and change of effective screening.[23]

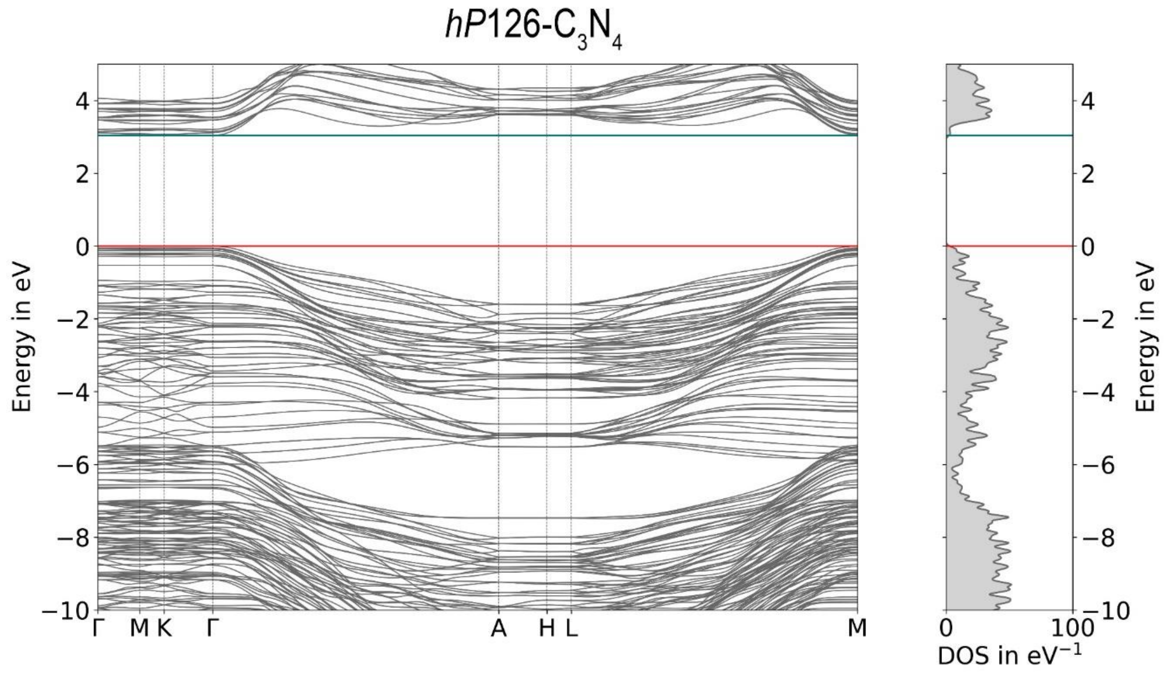

Figure S16: Electronic band structures and density of states at ambient pressure-temperature conditions for *hP*126-C_3N_4 shifted with respect to the highest occupied state. The vertical lines indicate the highest occupied (red) and lowest unoccupied (teal) state.

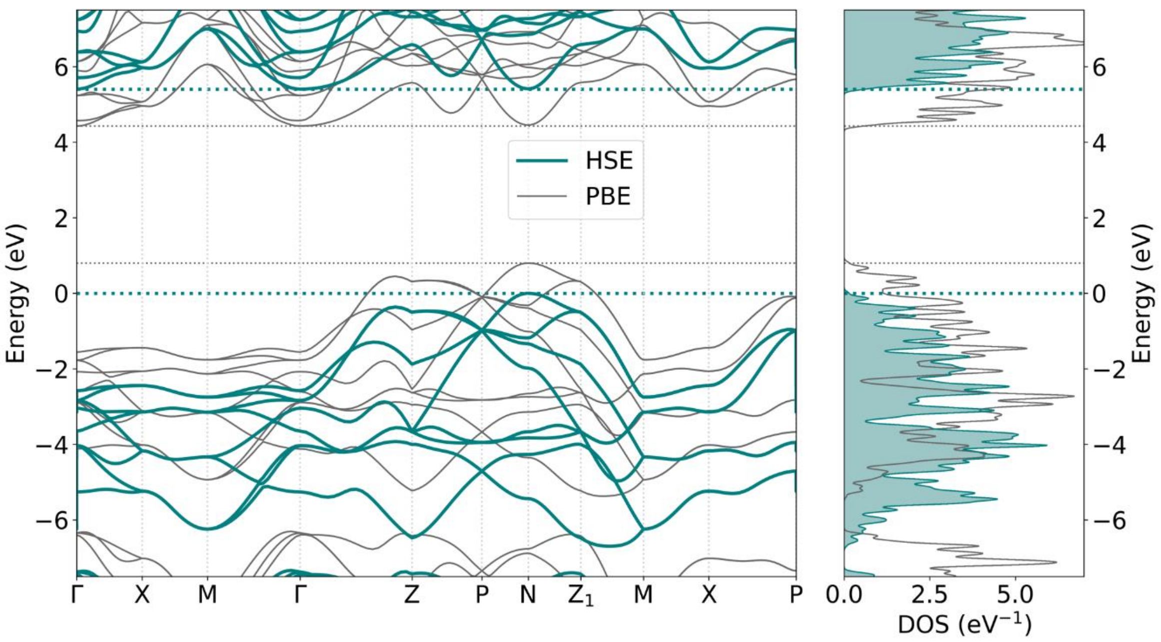

Figure S17: Electronic band structures and density of states using PBE (grey) and HSE (teal) at ambient pressure-temperature conditions for *tI*24-CN_2 shifted with respect to the highest occupied state obtained with HSE. The dotted horizontal lines indicate the highest occupied and lowest unoccupied state. *tI*24-CN_2 could be a direct or indirect band gap material as the difference between the energies of the conduction band at Γ and N points of the Brillouin zone is within the computational accuracy.

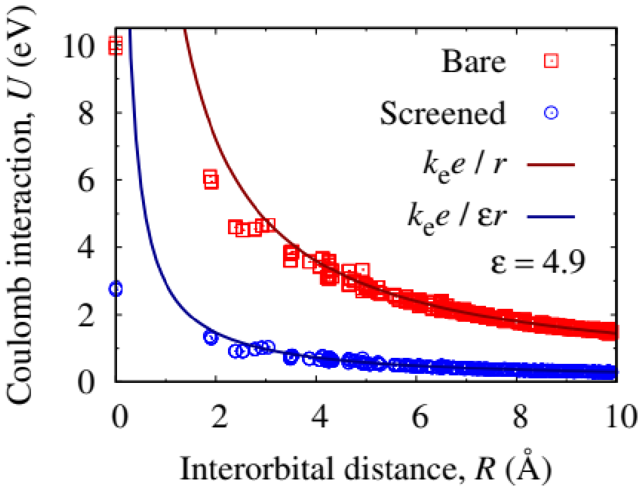

Figure S18: Bare and screened effective Coulomb interaction calculated between the relevant Wannier orbitals shown as a function of the interorbital distance in *tI*14-C_3N_4. The screening effects are considered within the constrained random phase approximation (cRPA). Squares and circles correspond to the bare and screened interactions, respectively. The lines correspond to the classical $U(r)=k_e e/\varepsilon r$ Coulomb interactions, where k_e is the Coulomb constant, and ε is the effective screening constant. Apart from the nearest-neighbour interactions, the distance-dependence of the interactions is well-described by the classical form of the Coulomb interaction $U(r) = k_e e/\varepsilon r$, where ε is the effective screening constant. The on-site and nearest-neighbour screened interactions are estimated to be $U_{00} = 2.9$ eV and $U_{01} = 1.4$ eV, respectively. Ignoring the nonlocal contributions, the interaction U_{00} is comparable to the band width W = 3.1 eV. However, for systems with solely *s* and *p* electrons and with a high degree of electron delocalization, it is more appropriate to rely on the reduced interaction $U^* = U_{00}-U_{01} = 1.5$ eV when considering mapping on the effective (on-site) Hubbard model.[24] In this situation, U* ~ W/2, suggesting that the strength of the electron-electron correlations in *tI*14-C_3N_4 can be comparable to that in a 3d-transition metal, like Ni.[25] Note that the ratios U/W and U*/W for this material are roughly twice larger than for graphene which means essentially stronger correlation effects, even not taking into account specific correlation effects which may be related to the flat band for the corresponding doping range.[24]

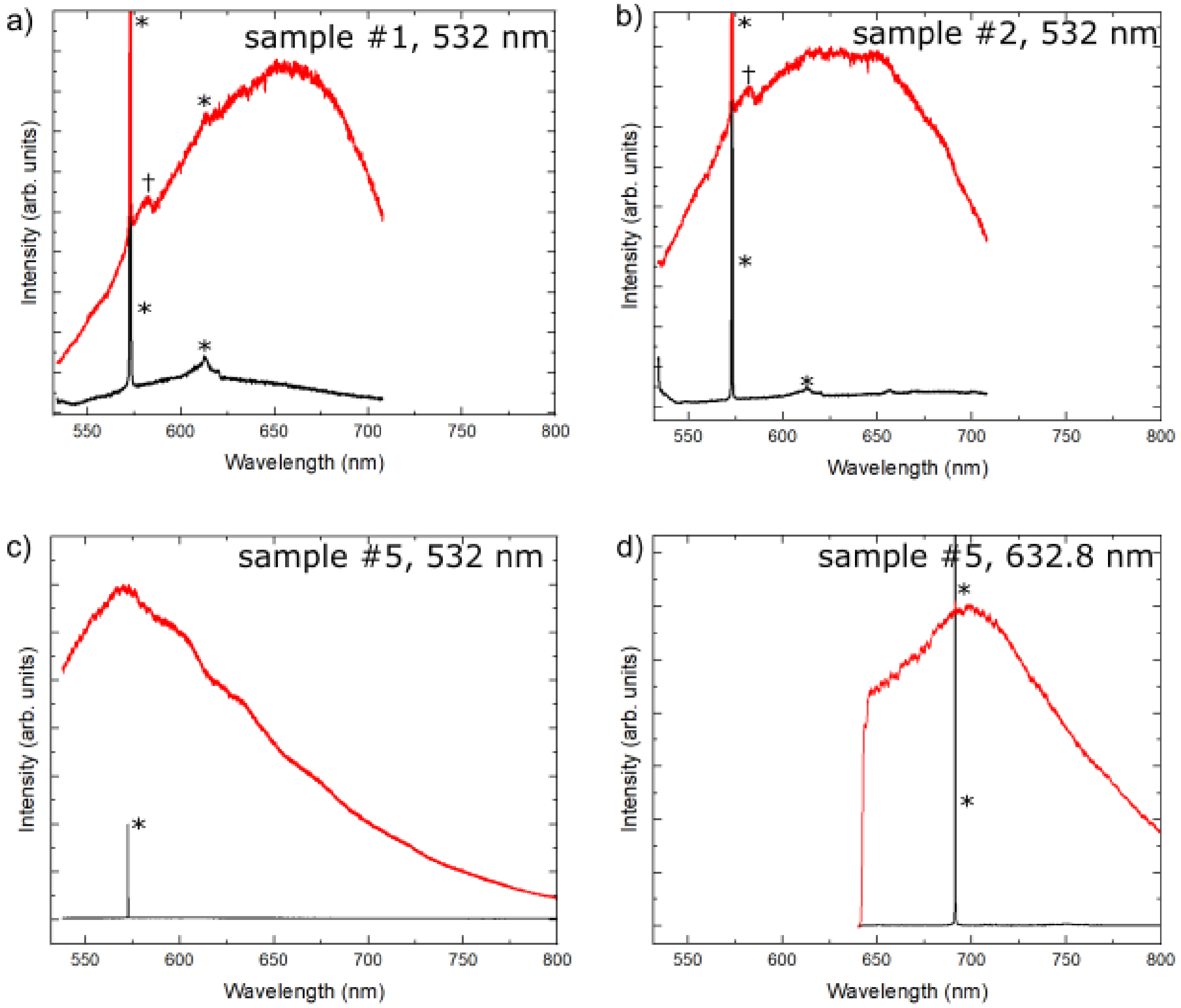

Figure S19: Photoluminescence spectra collected at ambient conditions on a) sample #1 (*tI*14-C_3N_4 and *hP*126-C_3N_4, 532 nm excitation wavelength), b) sample #2 (*oP*8-CN, 532 nm excitation wavelength), c) sample #5 (*hP*126-C_3N_4, 532 nm excitation wavelength) and, d) sample #5 (*hP*126-C_3N_4, 632.8 nm excitation wavelength). The intensity drop in d) near 632 nm is due to filters blocking the incident laser's wavelength (*i.e.* 632.8 nm). The black spectra serve as a reference and were collected either on the rhenium gasket (a, b) or the sample-free diamond anvil (c, d). The red spectra were collected on a transformed part of the sample. The asterisks indicate Raman peaks belonging to the diamond anvils, while the cross represents a peak from leftover, untransformed and amorphous cross-linked recovered TCNE (Figure S21).[26] All spectra within a graph have been collected using the same laser power and acquisition times or, in the case of c), automatically renormalized in LabSpec. For ease of comparison, all graphs have the same *x*-axis range. Given that the excitation laser is of lower energy than the calculated band gap of these carbon nitrides, the photoluminescence signal is expected to come from colour centres.

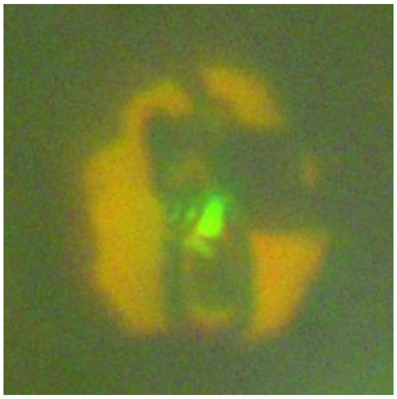

Figure S20: Microphotograph of sample #4 at 120 GPa during laser heating. Green coloured light is produced when irradiating the sample with the laser-heating laser (λ = 1064 nm). This is explained by the non-linear optical properties of the formed compound, *tI*14-C_3N_4, producing green photons through second-order harmonic generation. This is due to the non-centrosymmetric crystal structure of the *tI*14-C_3N_4 solid. *hP*126-C_3N_4 is also present in sample #4 but, given its centrosymmetric structure, is not expected to contribute to the signal produced by the second-order harmonic generation.

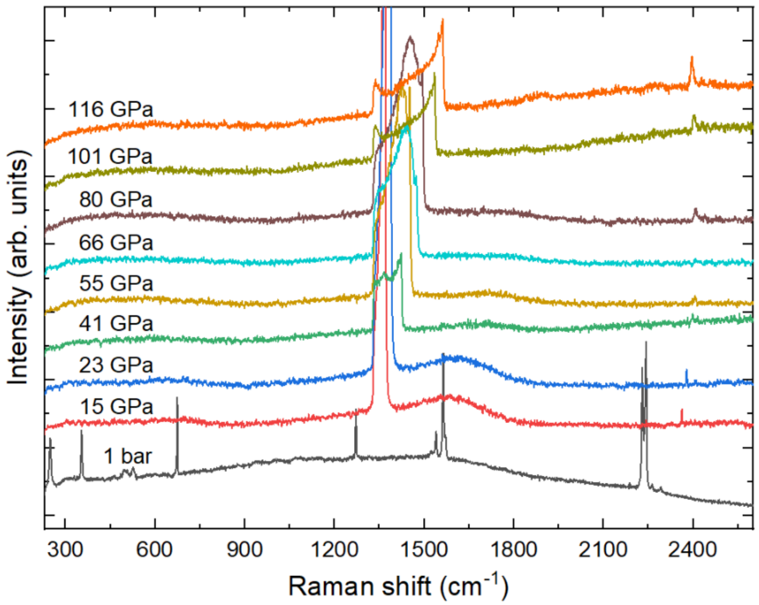

Figure S21: Raman spectra collected from sample #1 upon compression, with the exception of the spectrum at 1 bar. The latter was collected on a TCNE piece before sample loading, and the modes observed are in perfect agreement with those previously reported for TCNE.[27,28] At 15 GPa and above, only the Raman modes of the diamond (centred around ~1400 cm^{-1}) anvils and molecular nitrogen (centred around ~2350 cm^{-1}) are visible; the modes of TCNE are known to disappear above 14 GPa, presumably upon the formation of an amorphous polymer with random cross-linked TCNE units.[26]

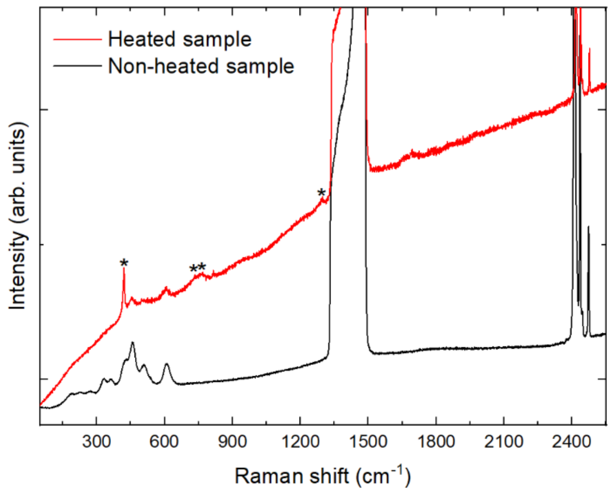

Figure S22: Raman spectra collected on sample #2, comprised of TCNE and N_2, at 72 GPa. The black spectrum was collected on a non laser-heated portion of TCNE and the peaks of molecular nitrogen are clearly seen. As described in the caption of Figure S21, no modes of TCNE are visible above about 14 GPa. The red spectrum was collected on laser-heated TCNE and a number of new Raman modes, identified by asterisks, are visible. All of these modes match those previously observed for *oP*8-CN.[9] Other visible modes belong to molecular nitrogen.

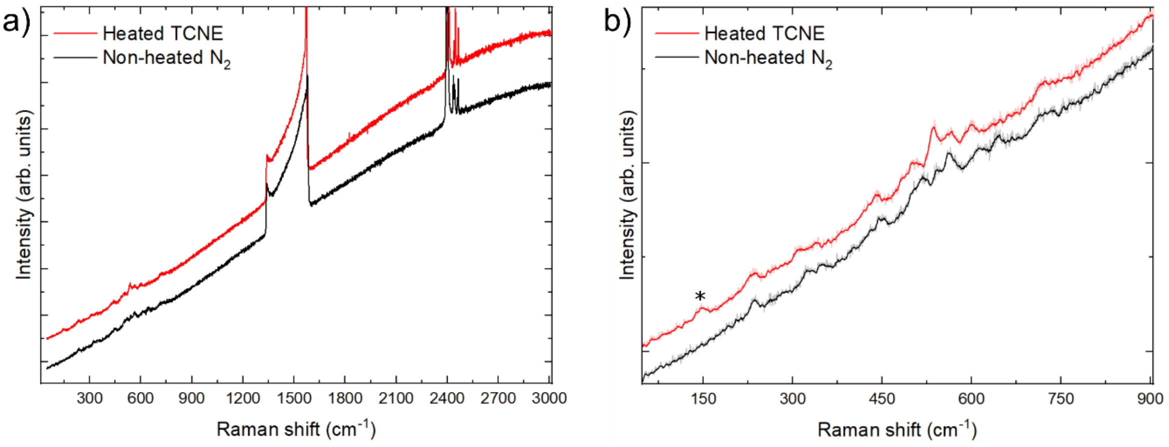

Figure S23: Raman spectra collected on sample #1 at 124 GPa. a) Full Raman spectra, where the black and red lines represent non-heated and heated portions of the sample. In both cases, the spectra are dominated by molecular nitrogen, as clearly seen by the intense modes near 2435 cm^{-1}. b) Enlargement of the spectral region between 45 and 905 cm^{-1}. A single peak, centred on 150 cm^{-1} and identified by an asterisk, can be identified as not belonging to molecular nitrogen. As described in the caption of Figure S21, no modes of TCNE are visible above about 14 GPa. These measurements highlight the difficulties of identifying compounds based on Raman measurements. Nonetheless, they are essential as they provide a reference for future experiments.

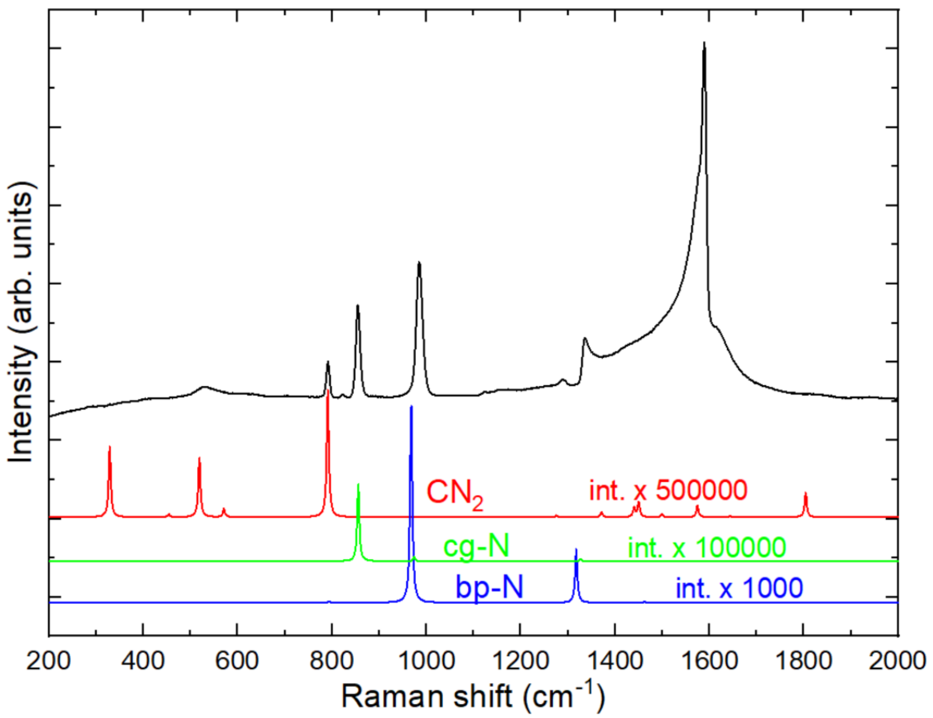

Figure S24: Experimental Raman spectrum (black) collected on sample #3 at 134 GPa along with calculated Raman spectra of *tI*24-CN_2, cg-N and bp-N, respectively in red, green and blue. For clarify, the intensity of the spectra of *tI*24-CN_2, cg-N and bp-N were respectively multiplied by 500000, 100000 and 1000. The known cg-N[10] and bp-N[11,15] nitrogen allotropes explain all sharp peaks but the one at 792 cm^{-1}, which matches with the most intense peak of the calculated *tI*24-CN_2 spectrum. There is also a broad mode at 530 cm^{-1} that could be attributed to the calculated modes of *tI*24-CN_2 at the same frequency. Theoretical calculations suggest that the peak of *tI*24-CN_2 at 792 cm^{-1}—unlike all other modes of *tI*24-CN_2—is orientation dependant. This provides an explanation as to why the other modes of *tI*24-CN_2 are of much lower intensity, when visible. A fourth phase in this sample, PN_2,[13] was identified by X-ray diffraction. As PN_2 is metallic,[13] its Raman modes are not expected to be experimentally measured.

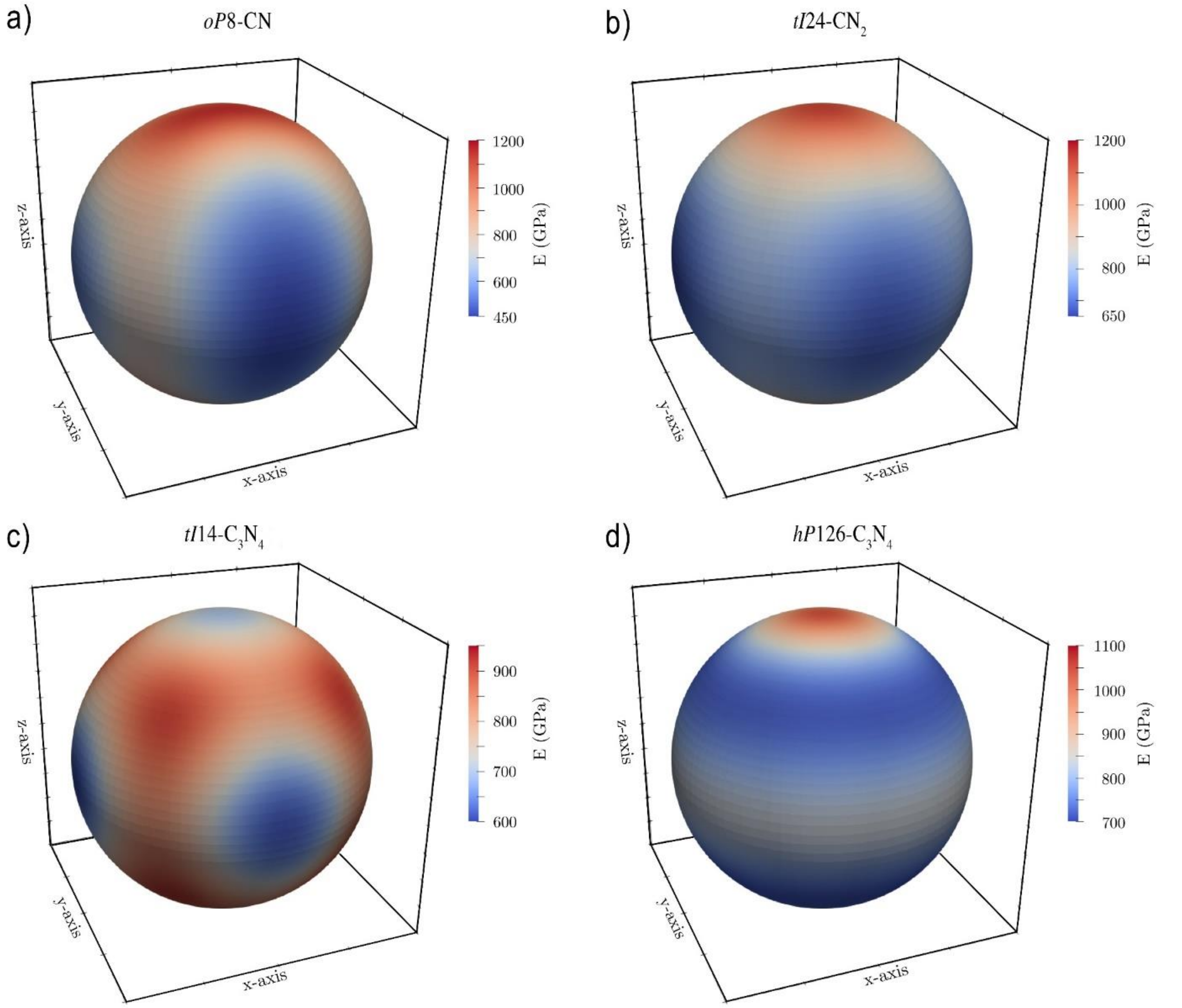

Figure S25: Directional dependence of Young's modulus (GPa) for a) *oP*8-CN, b) *tI*24-CN_2, c) *tI*14-C_3N_4 and d) *hP*126-C_3N_4.

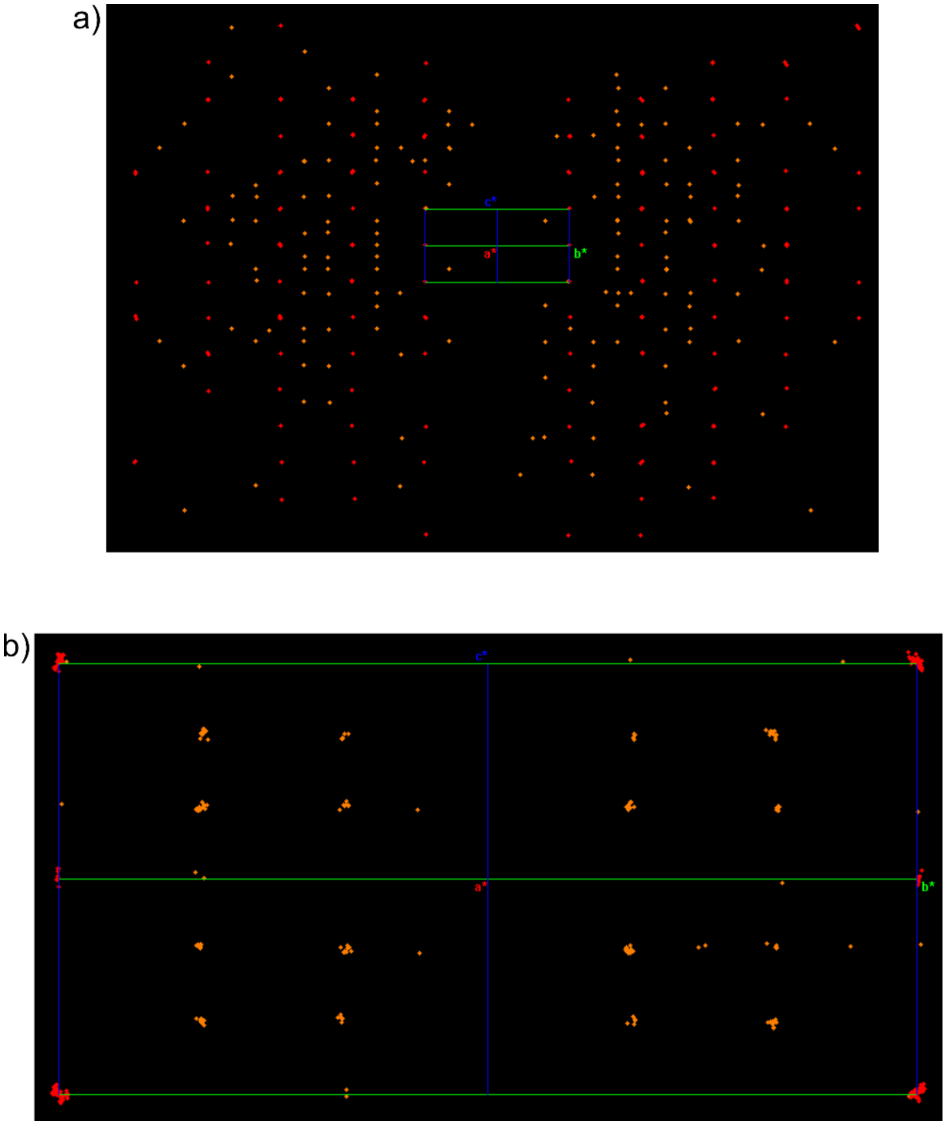

Figure S26: a) Ewald sphere with the reflections of a *tI*14-C_3N_4 crystal viewed along the *a** direction at 130 GPa. b) Ewald sphere with the reflections of a *tI*14-C_3N_4 crystal viewed along the *a** direction and collapsed within the unit cell, at 130 GPa. The reflections from the subcell, for which the reciprocal unit cell is drawn, corresponding to a tetragonal unit cell with parameters of *a* = *b* = 3.2225(3) Å and *c* = 6.4482(18) Å (V = 66.96(2) Å^3) are in red while those of the orthorhombic supercell with *a* = 13.6827(14) Å, *b* = 13.6728(11) Å and *c* = 19.346(4) Å (V = 3619.2(9) Å^3) are in orange.

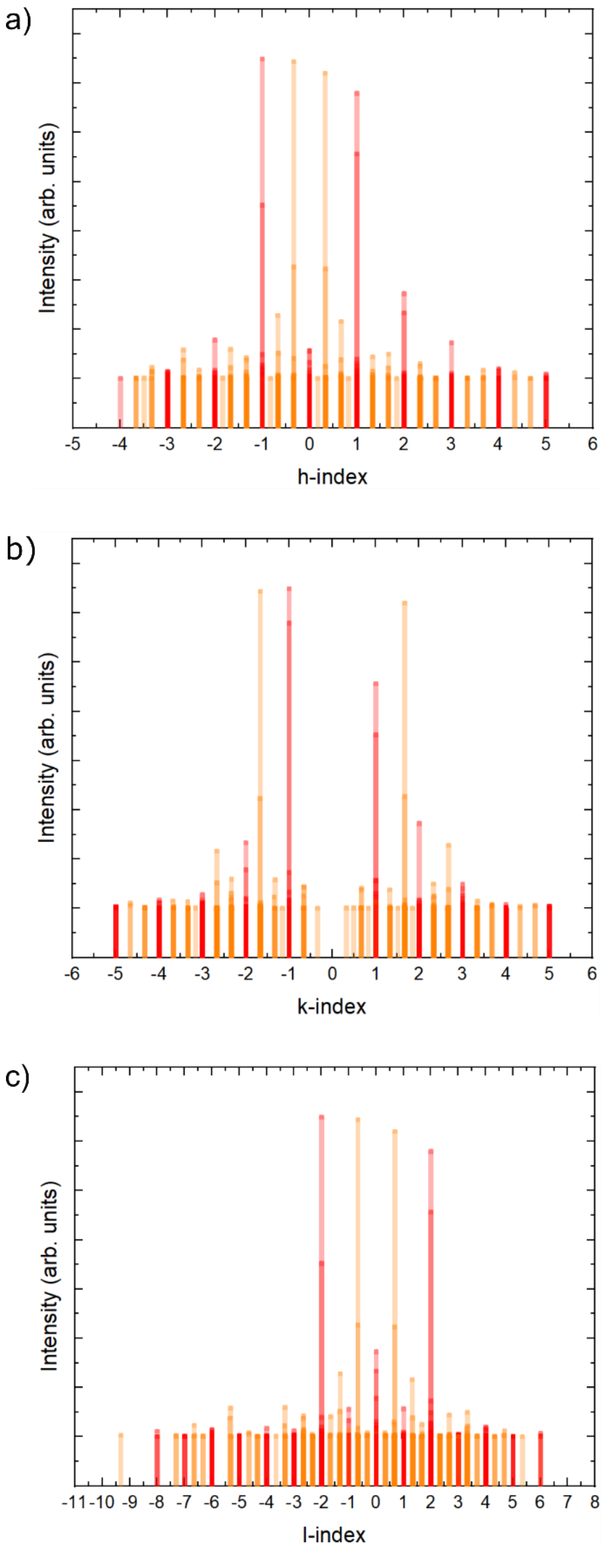

Figure S27: Intensity of the reflections of a *tI*14-C_3N_4 crystal at 130 GPa with respect to the h- (a), k- (b) and l-index (c). The integer index values correspond to the tetragonal subcell with $a = b = 3.2225(3)$ Å and $c = 6.4482(18)$ Å (V = 66.96(2) Å^3), in red, while in orange are those from the orthorhombic supercell with $a = 13.6827(14)$ Å, $b = 13.6728(11)$ Å and $c = 19.346(4)$ Å (V = 3619.2(9) Å^3).

Table S1: Crystallographic data for *tI*14-C_3N_4 at 124 GPa. Some parameters have both the experimental and the calculated value. The refinement was done with the Olex2 software.[29] The crystallographic data has been submitted under the deposition number CSD 2202358.

	***tI*14-C_3N_4**	
	**Exp.**	**Calc.**
Pressure (GPa)	124	124
Space group, #	*I*-42*m*, 121	*I*-42*m*, 121
Z	2	2
a (Å)	3.2285(6)	3.2266
b (Å)	3.2285(6)	3.2266
c (Å)	6.4526(16)	6.4565
V ($Å^3$)	67.26(3)	67.21
Refinement details		
Wavelength (λ, Å)	0.29521	
μ (mm^{-1})	0.097	
# measured/independent reflections (I ≥ 2*σ*)	101 / 64	
$(\sin\theta/\lambda)_{max}$ ($Å^{-1}$)	0.808	
R_{int} (%)	0.68	
$R[F^2 > 2\sigma(F^2)]$ (%)	4.46	
wR(*F*2) (%)	13.15	
S	1.235	
No. of parameters	6	
$\Delta\rho_{min}$, $\Delta\rho_{max}$ ($eÅ^{-3}$)	-0.336, 0.422	

**Atomic positions**				
Atom	Wyckoff position	Fractional atomic coordinates (x; y; z)		U_{iso} ($Å^2$)
		**Exp.**	**Calc.**	
C1	2a	0; 0; 0	0; 0; 0	0.0082(10)
C2	4d	0; 1/2; 1/4	0; 1/2; 1/4	0.0082(9)
N1	8i	0.2457(6); 0.2457(6); 0.1247(3)	0.2563; 0.2563; 0.1256	0.0073(8)

Table S2: Crystallographic data for *tI*14-C_3N_4 at 110 GPa. Some parameters have both the experimental and the calculated value. The refinement was done with the Jana2006 software.[30] The crystallographic data has been submitted under the deposition number CSD 2260018.

	***tI*14-C_3N_4**	
	**Exp.**	**Calc.**
Pressure (GPa)	110	110
Space group, #	*I*-42*m*, 121	*I*-42*m*, 121
Z	2	2
a (Å)	3.2270(6)	3.2466
b (Å)	3.2270(6)	3.2466
c (Å)	6.4854(16)	6.4949
V (Å^3)	67.54(2)	68.46
Refinement details		
Wavelength (λ, Å)	0.2905	
μ (mm^{-1})	0.095	
# measured/independent reflections (I ≥ 3σ)	89 / 47 (21)	
(sin θ/λ)$_{max}$ (Å^{-1})	0.712	
R_{int} (%)	15.56	
R_1 (%)	6.01	
wR_2 (%)	5.46	
R_1 (all data, %)	9.86	
wR_2 (all data, %)	7.08	
Goodness of fit	1.22	
No. of parameters	6	
$\Delta\rho_{min}$, $\Delta\rho_{max}$ (eÅ^{-3})	-0.34, 0.31	

**Atomic positions**				
Atom	Wyckoff position	Fractional atomic coordinates (x; y; z)		U_{iso} (Å^2)
		**Exp.**	**Calc.**	
C1	2a	0; 0; 0	0; 0; 0	0.011(2)
C2	4d	0; 1/2; 1/4	0; 1/2; 1/4	0.0114(17)
N1	8i	0.2438(12); 0.2438(12); 0.1246(11)	0.2428; 0.2428; 0.1295	0.0108(13)

Table S3: Crystallographic data for *hP*126-C_3N_4 at 124 GPa. Some parameters have both the experimental and the calculated value. The refinement was done with the Jana2006 software.[30] The crystallographic data has been submitted under the deposition number CSD 2202354.

	***hP*126-C_3N_4**	
	**Exp.**	**Calc.**
Pressure (GPa)	124	124
Space group, #	$P6_3/m$, 176	$P6_3/m$, 176
Z	18	18
a (Å)	17.690(8)	17.699
b (Å)	17.690(8)	17.699
c (Å)	2.2645(11)	2.2595
γ (°)	120	120
V (Å^3)	613.7(5)	613.0
Refinement details		
Wavelength (λ, Å)	0.29521	
μ (mm^{-1})	0.094	
# measured/independent reflections (I ≥ 3σ)	1989 / 757 (294)	
(sin θ/λ)$_{max}$ (Å^{-1})	0.884	
R_{int} (%)	8.01	
R_1 (%)	4.81	
wR_2 (%)	3.65	
R_1 (all data, %)	13.88	
wR_2 (all data, %)	4.67	
Goodness of fit	1.01	
No. of parameters	94	
$\Delta\rho_{min}$, $\Delta\rho_{max}$ (eÅ^{-3})	-0.41, 0.51	

**Atomic positions**				
Atom	Wyckoff position	Fractional atomic coordinates (x; y; z)		U_{iso} (Å^2)
		**Exp.**	**Calc.**	
C1	6h	0.3901(3); 0.2714(3); 1/4	0.3889; 0.2708; 1/4	0.0115(8)
C2	6h	0.2325(3); 0.4971(3); 1/4	0.2326; 0.4969; 1/4	0.0129(8)
C3	6h	0.2156(3); 0.2828(3); 1/4	0.2142; 0.2811; 1/4	0.0112(8)
C4	6h	0.0707(3); 0.2620(3); 1/4	0.0701; 0.2611; 1/4	0.0121(8)
C5	6h	0.2395(3); 0.6275(2); 1/4	0.2393; 0.6275; 1/4	0.0104(8)
C6	6h	0.4231(3); 0.1260(3); 1/4	0.4221; 0.1258; 1/4	0.0100(8)
C7	6h	0.0859(3); 0.1386(3); 1/4	0.0852; 0.1372; 1/4	0.0126(8)
C8	6h	0.0204(3); 0.4161(3); 1/4	0.0194; 0.4142; 1/4	0.0141(9)
C9	6h	0.5419(3); 0.0975(3); 1/4	0.5414; 0.0974; 1/4	0.0138(8)
N1	6h	0.4525(2); 0.0675(2); 1/4	0.4529; 0.0670; 1/4	0.0104(12)
N2	6h	0.2384(2); 0.2181(2); 1/4	0.2378; 0.2174; 1/4	0.0100(12)
N3	6h	0.0706(2); 0.5047(2); 1/4	0.0696; 0.5045; 1/4	0.0086(12)
N4	6h	0.5792(2); 0.1884(2); 1/4	0.5795; 0.1879; 1/4	0.0096(7)
N5	6h	0.1102(2); 0.0026(2); 1/4	0.1077; 0.0030l 1/4	0.0156(14)

N6	6h	0.2859(2); 0.5840(2); 1/4	0.2855; 0.5846; 1/4	0.0103(13)
N7	6h	0.1243(2); 0.2287(2); 1/4	0.1245; 0.2279; 1/4	0.0094(6)
N8	6h	0.3305(2); 0.0853(2); 1/4	0.3317; 0.0857; 1/4	0.0101(12)
N9	6h	0.3796(2); 0.3437(2); 1/4	0.3789; 0.3433; 1/4	0.0123(13)
N10	6h	0.4753(2); 0.2951(2); 1/4	0.4751; 0.2946; 1/4	0.0128(14)
N11	6h	0.1290(2); 0.3524(2); 1/4	0.1285; 0.3507; 1/4	0.0128(13)
N12	6h	0.2806(2); 0.4543(3); 1/4	0.2787; 0.4542; 1/4	0.0136(13)

Table S4: Crystallographic data for *hP*126-C_3N_4 at 74 GPa. Some parameters have both the experimental and the calculated value. The refinement was done with the Jana2006 software.[30] The crystallographic data has been submitted under the deposition number CSD 2260017.

	***hP*126-C_3N_4**	
	**Exp.**	**Calc.**
Pressure (GPa)	74	74
Space group, #	$P6_3/m$, 176	$P6_3/m$, 176
Z	18	18
a (Å)	18.096(8)	18.113
b (Å)	18.096(8)	18.113
c (Å)	2.2955(11)	2.3100
γ (°)	120	120
V (Å^3)	651.0(5)	656.3
Refinement details		
Wavelength (λ, Å)	0.29521	
μ (mm^{-1})	0.088	
# measured/independent reflections (I ≥ 3σ)	1326 / 511 (273)	
(sin θ/λ)$_{max}$ (Å^{-1})	0.625	
R_{int} (%)	5.14	
R_1 (%)	4.73	
wR_2 (%)	4.27	
R_1 (all data, %)	10.87	
wR_2 (all data, %)	4.66	
Goodness of fit	1.53	
No. of parameters	64	
$\Delta\rho_{min}$, $\Delta\rho_{max}$ (eÅ^{-3})	-0.39, 0.37	
**Atomic positions**		

Atom	Wyckoff position	Fractional atomic coordinates (x; y; z)		U_{iso} (Å^2)
		**Exp.**	**Calc.**	
C1	6h	0.3883(3); 0.2712(3); 1/4	0.3893; 0.2714; 1/4	0.0146(10)
C2	6h	0.2337(3); 0.4959(3); 1/4	0.2339; 0.4962; 1/4	0.0101(10)
C3	6h	0.2133(3); 0.2804(3); 1/4	0.2136; 0.2805; 1/4	0.0130(10)
C4	6h	0.0695(3); 0.2607(3); 1/4	0.0696; 0.2611; 1/4	0.0123(11)
C5	6h	0.2393(3); 0.6266(3); 1/4	0.2396; 0.6270; 1/4	0.0109(11)
C6	6h	0.4218(3); 0.1252(3); 1/4	0.4213; 0.1246; 1/4	0.0147(11)
C7	6h	0.0833(3); 0.1364(3); 1/4	0.0842; 0.1372; 1/4	0.0171(12)
C8	6h	0.0202(3); 0.4161(3); 1/4	0.0200; 0.4160; 1/4	0.0097(10)
C9	6h	0.5415(3); 0.0981(3); 1/4	0.5410; 0.0980; 1/4	0.0142(10)
N1	6h	0.4522(3); 0.0664(3); 1/4	0.4525; 0.0665; 1/4	0.0189(10)
N2	6h	0.2369(2); 0.2174(2); 1/4	0.2376; 0.2175; 1/4	0.0102(9)
N3	6h	0.0702(3); 0.5060(3); 1/4	0.0701; 0.5059; 1/4	0.0121(9)
N4	6h	0.5804(2); 0.1882(2); 1/4	0.5804; 0.1883; 1/4	0.0122(10)
N5	6h	0.1092(2); 0.0048(2); 1/4	0.1082; 0.0040; 1/4	0.0128(9)

N6	6h	0.2855(3); 0.5839(3); 1/4	0.2858; 0.5841; 1/4	0.0147(9)
N7	6h	0.1239(3); 0.2284(3); 1/4	0.1238; 0.2279; 1/4	0.0176(9)
N8	6h	0.3310(3); 0.0857(3); 1/4	0.3310; 0.0858; 1/4	0.0135(9)
N9	6h	0.3816(2); 0.3449(2); 1/4	0.3822; 0.3450; 1/4	0.0129(9)
N10	6h	0.4743(2); 0.2921(3); 1/4	0.4741; 0.2915; 1/4	0.0118(9)
N11	6h	0.1271(3); 0.3508(2); 1/4	0.1261; 0.3504; 1/4	0.0120(10)
N12	6h	0.2792(3); 0.4533(3); 1/4	0.2794; 0.4533; 1/4	0.0122(9)

Table S5: Crystallographic data for *oP*8-CN at 72 GPa. Some parameters have both the experimental and the calculated value. The refinement was done with the Olex2 software.[29] The crystallographic data has been submitted under the deposition number CSD 2202356.

	***oP*8-CN**			
	**Exp.**		**Calc.**	
Pressure (GPa)	72		72	
Space group, #	*Pnnm*, 58		*Pnnm*, 58	
Z	4		4	
a (Å)	4.892(4)		4.9205	
b (Å)	3.7841(12)		3.7724	
c (Å)	2.2880(9)		2.2878	
V ($Å^3$)	42.36(4)		42.47	
Refinement details				
Wavelength (λ, Å)	0.29521			
μ (mm^{-1})	0.087			
# measured/independent reflections ($I \geq 2\sigma$)	55 / 48 (43)			
$(\sin \theta/\lambda)_{max}$ ($Å^{-1}$)	0.818			
R_{int} (%)	0.47			
$R[F^2 > 2\sigma(F^2)]$ (%)	6.63			
$wR(F2)$ (%)	19.21			
S	1.218			
No. of parameters	7			
$\Delta\rho_{min}$, $\Delta\rho_{max}$ ($eÅ^{-3}$)	-0.390, 0.507			
**Atomic positions**				
Atom	Wyckoff position	Fractional atomic coordinates (x; y; z)		U_{iso} ($Å^2$)
		**Exp.**	**Calc.**	
C1	4g	0.1449(8); 0.0585(5); 0	0.1452; 0.05911; 0	0.0116(12)
N1	4g	0.8205(8); 0.7395(5); 1/2	0.8194; 0.7394; 1/2	0.0112(13)

Table S6: Crystallographic data for *tI*24-CN_2 at 134 GPa. Some parameters have both the experimental and the calculated value. The refinement was done with the Jana2006 software.[30] The crystallographic data has been submitted under the deposition number CSD 2202360.

	***tI*24-CN_2**			
	**Exp.**		**Calc.**	
Pressure (GPa)	134		134	
Space group, #	*I*-42*d*, 122		*I*-42*d*, 122	
Z	8		8	
a (Å)	5.9864(13)		5.9472	
b (Å)	5.9864(13)		5.9472	
c (Å)	3.230(2)		3.2535	
V ($Å^3$)	115.75(8)		115.07	
Refinement details				
Wavelength (λ, Å)	0.3738			
μ (mm^{-1})	0.118			
# measured/independent reflections (I ≥ 3σ)	135 / 88 (64)			
$(\sin \theta/\lambda)_{max}$ ($Å^{-1}$)	0.900			
R_{int} (%)	5.42			
R_1 (%)	8.58			
wR_2 (%)	7.69			
R_1 (all data, %)	11.68			
wR_2 (all data, %)	7.81			
Goodness of fit	3.07			
No. of parameters	12			
$\Delta\rho_{min}$, $\Delta\rho_{max}$ ($eÅ^{-3}$)	-0.69, 0.72			
**Atomic positions**				

Atom	Wyckoff position	Fractional atomic coordinates (x; y; z)		U_{iso} ($Å^2$)
		**Exp.**	**Calc.**	
C1	8d	0.0581(13); 1/4; 1/8	0.0571; 1/4; 1/8	0.0167(15)
N1	16e	0.1849(8); 0.5646(7); 0.0307(16)	0.1857; 0.5648; 0.0341	0.0193(14)

Table S7: Average bond lengths and bond angles for *oP*8-CN, *tI*14-C_3N_4, *hP*126-C_3N_4 and *tI*24-CN_2 at one of their pressure of synthesis and at 1 bar.

**Compounds**	***oP*8-CN**	***tI*14-C_3N_4**	***hP*126-C_3N_4**	***tI*24-CN_2**
**Synthesis pressure (GPa)**	72	124	124	134
Average C-N bond (Å)	1.418(5)	1.390(2)	1.376(5)	1.384(7)
C-C bond (Å)	1.485(8)	--	--	--
N-N bond (Å)	--	--	--	1.272(11)
Average N/C-C-N angle (°)	109.43(9)	109.5(1)	109.43(7)	109.5(3)
Average N/C-N-C angle (°)	117.63(4)	110.0(1)	117.5(1)	117.3(4)
**1 bar**				
Average C-N bond (Å)	1.452(2)	1.470(2)	1.461(5)	1.477(9)
C-C bond (Å)	1.569(8)	--	--	--
N-N bond (Å)	--	--	--	1.359(12)
Average N/C-C-N angle (°)	109.47(5)	109.49(9)	109.45(8)	109.5(3)
Average N/C-N-C angle (°)	118.62(3)	111.9(1)	118.3(1)	117.9(4)
C-N bond order (average)*	0.96	0.95	0.95	0.94
C-C bond order (average)*	0.83	--	--	--
N-N bond order (average)*	--	--	--	1.04

*Integrated crystal orbital bond index (ICOBI), calculated employing the LOBSTER software[17,18]

Table S8: Experimental pressure-volume data for *oP*8-CN, *tI*14-C_3N_4, *hP*126-C_3N_4 and *tI*24-CN_2 determined from single-crystal X-ray diffraction measurements.

Phase	Pressure (GPa)	a (Å)	b (Å)	c (Å)	Volume ($Å^3$)	# reflections
*oP*8-CN	80	4.892(4)	3.7841(12)	2.2880(9)	42.24(7)	122
*oP*8-CN	72	4.817(8)	3.7938(9)	2.2858(4)	42.36(4)	55
*oP*8-CN	52	4.927(9)	3.836(3)	2.3286(17)	44.01(9)	82
*oP*8-CN	3	5.2834(3)	3.938(12)	2.37284(13)	49.369(7)	105
*oP*8-CN	0.0001	5.282(4)	3.9353(12)	2.3719(9)	49.30(4)	281
*tI*14-C_3N_4	130	3.2266(6)	3.2266(6)	6.4531(16)	67.18(2)	109
*tI*14-C_3N_4	124	3.2285(6)	3.2285(6)	6.4526(16)	67.26(3)	101
*tI*14-C_3N_4	110	3.2270(6)	3.2270(6)	6.4854(16)	67.54(2)	89
*tI*14-C_3N_4	106	3.243(2)	3.243(2)	6.500(6)	68.36(10)	55
*tI*14-C_3N_4	78	3.2727(15)	3.2727(15)	6.607(5)	70.76(7)	75
*tI*14-C_3N_4	32	3.3611(6)	3.3611(6)	6.736(2)	76.10(3)	90
*tI*14-C_3N_4	7	3.44548(12)	3.44548(12)	6.85687(6)	81.422(8)	120
*tI*14-C_3N_4	0.0001	3.4680(6)	3.4680(6)	6.8970(16)	82.95(3)	262
*hP*126-C_3N_4	130	17.6995(12)	17.6995(12)	2.2598(11)	613.7(5)	848
*hP*126-C_3N_4	124	17.690(8)	17.690(8)	2.2645(11)	613.08(6)	1989
*hP*126-C_3N_4	106	17.893(11)	17.893(11)	2.2698(16)	629.4(7)	78
*hP*126-C_3N_4	78	17.987(16)	17.987(16)	2.317(3)	649(1)	138
*hP*126-C_3N_4	74	18.096(8)	18.096(8)	2.2955(11)	651.0(5)	1326
*hP*126-C_3N_4	32	18.56(5)	18.56(5)	2.381(3)	710(3)	53
*hP*126-C_3N_4	7	18.9184(12)	18.9184(12)	2.4012(2)	744.27(9)	199
*hP*126-C_3N_4	0.0001	18.978(8)	18.978(8)	2.4083(11)	751.20(5)	2942
*tI*24-CN_2	137	5.9471(13)	5.9471(13)	3.250(2)	114.94(8)	154
*tI*24-CN_2	134	5.9864(13)	5.9864(13)	3.230(2)	115.75(8)	135
*tI*24-CN_2	119	6.019(4)	6.019(4)	3.281(3)	118.85(15)	105
*tI*24-CN_2	86	6.094(2)	6.094(2)	3.286(2)	122.03(10)	183
*tI*24-CN_2	18	6.331(11)	6.331(11)	3.403(7)	136.4(4)	19
*tI*24-CN_2	0.0001	6.4559(13)	6.4559(13)	3.426(2)	142.79(9)	82

Table S9: Crystallographic data for *oP*8-CN at 1 bar. Some parameters have both the experimental and the calculated value. The refinement was done with the Jana2006 software.[30] The crystallographic data has been submitted under the deposition number CSD 2202355.

	***oP*8-CN**	
	**Exp.**	**Calc.**
Pressure (bar)	1	1
Space group, #	*Pnnm*, 58	*Pnnm*, 58
Z	4	4
a (Å)	5.282(4)	5.321
b (Å)	3.9353(12)	3.9459
c (Å)	2.3719(9)	2.3734
V (Å^3)	49.30(4)	49.82
Refinement details		
Wavelength (λ, Å)	0.2843	
μ (mm^{-1})	0.073	
# measured/independent reflections (I ≥ 3σ)	281 / 115 (50)	
(sin θ/λ)$_{max}$ (Å^{-1})	1.055	
R_{int} (%)	8.75	
R_1 (%)	4.84	
wR_2 (%)	3.65	
R_1 (all data, %)	13.44	
wR_2 (all data, %)	4.75	
Goodness of fit	0.91	
No. of parameters	10	
$\Delta\rho_{min}$, $\Delta\rho_{max}$ (eÅ^{-3})	-0.31, 0.31	

**Atomic positions**				
Atom	Wyckoff position	Fractional atomic coordinates (x; y; z)		U_{iso} (Å^2)
		**Exp.**	**Calc.**	
C1	4g	0.1439(7); 0.0495(5); 0	0.1460; 0.0487; 0	0.0125(5)
N1	4g	0.8102(6); 0.7546(4); 1/2	0.8079; 0.7578; 1/2	0.0115(7)

Table S10: Crystallographic data for *tI*14-C_3N_4 at 1 bar. Some parameters have both the experimental and the calculated value. The refinement was done with the Jana2006 software.[30] The crystallographic data has been submitted under the deposition number CSD 2202357.

	***tI*14-C_3N_4**	
	**Exp.**	**Calc.**
Pressure (bar)	1	1
Space group, #	*I*-42*m*, 121	*I*-42*m*, 121
Z	2	2
a (Å)	3.4680(6)	
b (Å)	3.4680(6)	
c (Å)	6.8970(16)	
V ($Å^3$)	82.95(3)	
Refinement details		
Wavelength (λ, Å)	0.2843	
μ (mm^{-1})	0.077	
# measured/independent reflections (I ≥ 3*σ*)	262 / 133 (81)	
$(\sin \theta/\lambda)_{max}$ ($Å^{-1}$)	1.064	
R_{int} (%)	5.58	
R_1 (%)	4.51	
wR_2 (%)	3.87	
R_1 (all data, %)	6.79	
wR_2 (all data, %)	4.07	
Goodness of fit	1.37	
No. of parameters	11	
$\Delta\rho_{min}$, $\Delta\rho_{max}$ ($eÅ^{-3}$)	-0.37, 0.42	

**Atomic positions**				
Atom	Wyckoff position	Fractional atomic coordinates (x; y; z)		U_{iso} ($Å^2$)
		**Exp.**	**Calc.**	
C1	2a	0; 0; 0	0; 0; 0	0.0080(11)
C2	4d	0; 1/2; 1/4	0; 1/2; 1/4	0.0059
N1	8i	0.2347(4); 0.2347(4); 0.1273(4)	0.2335; 0.2335; 0.1290	0.0070(4)

Table S11: Crystallographic data for *hP*126-C_3N_4 at 1 bar. Some parameters have both the experimental and the calculated value. The refinement was done with the Jana2006 software.[30] The crystallographic data has been submitted under the deposition number CSD 2202353.

	***hP*126-C_3N_4**	
	**Exp.**	**Calc.**
Pressure (bar)	1	1
Space group, #	*P*6_3/*m*, 176	*P*6_3/*m*, 176
Z	18	18
a (Å)	18.978(8)	19.026
b (Å)	18.978(8)	19.026
c (Å)	2.4083(11)	2.4180
γ (°)	120	120
V (Å^3)	751.2(6)	758.0
Refinement details		
Wavelength (λ, Å)	0.2843	
μ (mm^{-1})	0.077	
# measured/independent reflections (I ≥ 3*σ*)	2942 / 958 (478)	
(sin θ/λ)$_{max}$ (Å^{-1})	0.734	
R_{int} (%)	6.66	
R_1 (%)	5.92	
wR_2 (%)	6.02	
R_1 (all data, %)	11.89	
wR_2 (all data, %)	6.64	
Goodness of fit	1.59	
No. of parameters	94	
$\Delta\rho_{min}$, $\Delta\rho_{max}$ (eÅ^{-3})	-0.51, 1.49	

**Atomic positions**				
Atom	Wyckoff position	Fractional atomic coordinates (x; y; z)		U_{iso} (Å^2)
		**Exp.**	**Calc.**	
C1	6h	0.3909(2); 0.2734(2); 1/4	0.3901; 0.2727; 1/4	0.0092(7)
C2	6h	0.2350(2); 0.4952(3); 1/4	0.2363; 0.4943; 1/4	0.0085(8)
C3	6h	0.2130(3); 0.2804(2); 1/4	0.2122; 0.2794; 1/4	0.0096(8)
C4	6h	0.0682(2); 0.2622(3); 1/4	0.0682; 0.2612; 1/4	0.0102(8)
C5	6h	0.2406(3); 0.6265(2); 1/4	0.2398; 0.6259; 1/4	0.0095(8)
C6	6h	0.4200(3); 0.1223(2); 1/4	0.4197; 0.1222; 1/4	0.0090(8)
C7	6h	0.0837(3); 0.1397(3); 1/4	0.0821; 0.1371; 1/4	0.0122(8)
C8	6h	0.0213(3); 0.4198(3); 1/4	0.0218; 0.4199; 1/4	0.0099(8)
C9	6h	0.5413(2); 0.0990(2); 1/4	0.5401; 0.0986; 1/4	0.0091(7)
N1	6h	0.4517(2); 0.0660(2); 1/4	0.4514; 0.0651; 1/4	0.0098(14)
N2	6h	0.2386(2); 0.2190(2); 1/4	0.2372; 0.2180; 1/4	0.0100(13)
N3	6h	0.0709(2); 0.5088(2); 1/4	0.0705; 0.5087; 1/4	0.0083(13)
N4	6h	0.5817(2); 0.1889(2); 1/4	0.5825; 0.1888; 1/4	0.0097(7)
N5	6h	0.1120(2); 0.0059(2); 1/4	0.1092; 0.0062; 1/4	0.0128(14)

N6	6h	0.2864(2); 0.5829(2); 1/4	0.2865; 0.5830; 1/4	0.0077(13)
N7	6h	0.1223(2); 0.2291(2); 1/4	0.1220; 0.2280; 1/4	0.0100(7)
N8	6h	0.3305(2); 0.0860(2); 1/4	0.3295; 0.0862; 1/4	0.0095(13)
N9	6h	0.3886(2); 0.3484(2); 1/4	0.3891; 0.3484; 1/4	0.0103(13)
N10	6h	0.4724(2); 0.2860(2); 1/4	0.4713; 0.2848; 1/4	0.0099(13)
N11	6h	0.1221(2); 0.3504(2); 1/4	0.1209; 0.3494; 1/4	0.0105(14)
N12	6h	0.2813(2); 0.4519(2); 1/4	0.2811; 0.4515; 1/4	0.0106(13)

Table S12: Crystallographic data for *tI*24-CN_2 at 1 bar. Some parameters have both the experimental and the calculated value. The refinement was done with the Jana2006 software.[30] The crystallographic data has been submitted under the deposition number CSD 2202359.

	***tI*24-CN_2**	
	**Exp.**	**Calc.**
Pressure (bar)	1	1
Space group, #	*I*-42*d*, 122	*I*-42*d*, 122
Z	8	8
a (Å)	6.4559(13)	6.4735
b (Å)	6.4559(13)	6.4735
c (Å)	3.426(2)	3.4353
V ($Å^3$)	142.79(9)	143.96
Refinement details		
Wavelength (λ, Å)	0.2843	
μ (mm^{-1})	0.078	
# measured/independent reflections (I ≥ 3*σ*)	82 / 49 (29)	
$(\sin \theta/\lambda)_{max}$ ($Å^{-1}$)	0.623	
R_{int} (%)	12.05	
R_1 (%)	7.62	
wR_2 (%)	8.87	
R_1 (all data, %)	11.59	
wR_2 (all data, %)	9.68	
Goodness of fit	0.89	
No. of parameters	7	
$\Delta\rho_{min}$, $\Delta\rho_{max}$ ($eÅ^{-3}$)	-0.33, 0.31	

**Atomic positions**				
Atom	Wyckoff position	Fractional atomic coordinates (x; y; z)		U_{iso} ($Å^2$)
		**Exp.**	**Calc.**	
C1	8d	0.0563(19); 1/4; 1/8	0.0568; 1/4; 1/8	0.024(4)
N1	16e	0.1867(9); 0.0687(9); 0.2165(16)	0.1885; 0.0692; 0.2143	0.018(3)

Table S13: Comparison of the bulk modulus, its pressure derivative and the ambient pressure volume between experiments and DFT calculations.

**Compounds**	***oP*8-CN**	***tI*14-C_3N_4**	***hP*126-C_3N_4**	***tI*24-CN_2**	**Diamond**
**DFT-calculations**					
K_0 (GPa)	334.20	375.86	393.10	399.36	440.09
K´	3.83	3.69	3.74	3.82	3.60
V_0 (per unit cell, Å^3)	49.79	83.80	758.22	143.96	45.40
**Experiments (V_0 fitted)**					
K_0 (GPa)	351(20)	407(26)	397(16)	429(28)	
K´ (fixed)	4	4	4	4	
V_0 (Å^3)	49.5(3)	82.3(6)	756(4)	142(1)	
**Experiments (V_0 fixed on experimental value)**					
K_0 (GPa)	365(9)	383(9)	417(6)	419(8)	
K´ (fixed)	4	4	4	4	
V_0 (Å^3)	49.30	82.95	751.2	142.79	

Table S14: Experimental and calculated bulk moduli (K_0) for the four carbon nitrides as well as hardness calculated via the model presented by Lyakhov and Oganov[31] (microscopic) and Chen *et al*.[32] (macroscopic) and band gaps calculated with HSE functional at ambient pressure-temperature conditions. Experimental and calculated values for diamond and c-BN are also provided. Fracture toughness (K_{IC}) is calculated from the elastic constants following the approach presented in Mazhnik *et al*.[33] The bulk moduli values were calculated from the anisotropic single-crystal elastic stiffness constants, and not from the equation of state. References are provided for all values obtained from the literature.

	**K_0 (GPa)**		**Microscopic hardness (GPa)**	**Macroscopic hardness (GPa)**	**Bandgap (eV) d: direct, i: indirect**	**K_{IC} (MPa $m^{1/2}$)**
	**Exp.**	**Calc.**				
**c-BN**	395(2)[34]	398[35]	63.2[36]	63.1 – 68.3[32]	6.1 (i)[37]	5.0[38]
**Diamond**	446(1)[8]	440	89.2[39]	92.9[32]	5.48 (i)[40]	5.3–6.7[38]
***oP*8-CN**	365(9)	344.6	78.0	53.4	5.3 (i)	4.3
***tI*24-CN_2**	419(8)	401.8	86.8	50.0	5.4 (i)	7.2
***tI*14-C_3N_4**	383(9)	377.2	80.4	52.9	4.3 (d)	6.1
***hP*126-C_3N_4**	417(6)	383.0	80.8	49.9	4.4 (d)	6.7

Table S15: Full piezoelectric stress (strain) moduli of *tI*24-CN_2 and *tI*14-C_3N_4 in units of C/m^2 (pm/V). The static or zero-frequency dielectric tensors (ε_0) along different crystallographic directions(ε_0, ε_2, ε_3), and the relative dielectric permittivity of materials (ε_r), are computed using the same density functional perturbation theory (DFPT) methods using VASP. The higher values of piezoelectric constants of *tI*14-C_3N_4, compared to *tI*24-CN_2, is attributed due to its lower shear elastic stiffness (G = 348.2 GPa (*tI*14-C_3N_4) < G = 352.6 GPa (*tI*24-CN_2), see Table S17). This allows a larger displacement of ions in response to a small elastic deformation, *i.e.*, enhanced electro-mechanical response. This can also be verified by looking at the effective Born charges on cation species (Z_C*). The transverse effective Born charges on the carbon atoms in *tI*24-CN_2 is Z_C* = 2.06 |e|, are lower than the effective Z_C* = 2.35 |e| in *tI*14-C_3N_4.

**Compound**	e_{14} (C/m^2), d_{14}(pm/V)	e_{25} (C/m^2), d_{25}(pm/V)	e_{36} (C/m^2), d_{36}(pm/V)	Z_C^* (|**e**|)	$\varepsilon_1, \varepsilon_2, \varepsilon_3$	ε_r
***tI*24-CN_2**	-0.35, -0.96	-0.35, -0.96	-0.22, -0.66	2.06	7.1, 7.1, 6.5	6.9
***tI*14-C_3N_4**	-0.77, -1.85	-0.77, -1.85	-0.63, -1.50	2.35	7.5, 7.5, 7.2	7.4

Table S16: The energy release of the reaction $C_xN_y \rightarrow x \cdot C + y/2 \cdot N_2$ at 300 K and P = 0 GPa, compared to that of the traditional high energy density materials TNT and RDX. The energy release was calculated with respect to a decomposition into graphite and molecular nitrogen.

**Compound**	***oP*8-CN**	***tI*24-CN_2**	***tI*14-C_3N_4**	***hP*126-C_3N_4**	**TNT**[41]	**RDX**[41]
Energy released in kJ/g	4.0	7.0	4.8	5.2	4.18	5.40

Table S17: Polycrystalline bulk (K_0), shear (G) and Young's (E) modulus (Hill average) and the calculated hardness following the macroscopic model by Chen *et al*.[32]

	K_0 (GPa)	G (GPa)	E (GPa)	H (GPa)
***oP*8-CN**	344.6	339.6	749.8	53.4
***tI*24-CN_2**	401.8	352.6	818.4	50.0
***tI*14-C_3N_4**	377.1	348.2	798.8	52.9
***hP*126-C_3N_4**	383.0	340.9	788.6	49.9

Table S18: Elastic constants (C_{ij}) calculated from energy-strain relationships with the finite difference method from (+/-) 1 and 2 % strain. The elastic constants are calculated at ambient pressure for all four compounds.

**Elastic constant C_{ij}**	***oP*8-CN**	***tI*24-CN_2**	***tI*14-C_3N_4**	***hP*126-C_3N_4**
C_{11} (GPa)	655.67	746.69	706.70	879.36
C_{22} (GPa)	521.78	746.69	706.71	879.28
C_{33} (GPa)	1190.49	1183.77	773.73	1060.99
C_{44} (GPa)	277.82	365.05	419.74	281.84
C_{55} (GPa)	441.93	365.05	419.74	281.86
C_{66} (GPa)	378.15	335.10	420.80	351.09
C_{12} (GPa)	201.38	209.29	212.01	178.55
C_{13} (GPa)	143.88	142.49	196.05	90.94
C_{14} (GPa)	-0.26	0.06	-1.24	-0.38
C_{15} (GPa)	0.37	-0.50	-0.15	-0.14
C_{16} (GPa)	-0.27	0.36	0.33	-0.84
C_{23} (GPa)	82.60	142.49	196.05	90.59
C_{24} (GPa)	0.09	-0.50	-0.16	-0.35
C_{25} (GPa)	0.03	0.06	-1.25	-0.06
C_{26} (GPa)	0.12	0.36	0.33	-0.79
C_{34} (GPa)	-0.32	-0.97	-0.12	-0.35
C_{35} (GPa)	2.61	-0.97	-0.12	-0.33
C_{36} (GPa)	0.89	0.94	-0.13	-0.02
C_{45} (GPa)	-0.22	-1.02	-1.31	0.03
C_{46} (GPa)	-0.24	-0.15	-1.12	-0.03

Table S19: Comparison of the calculated band gaps of the four carbon nitrides at ambient conditions based on either the PBE or HSE approximations.

	***oP*8-CN**	***tI*24-CN_2**	***tI*14-C_3N_4**	***hP*126-C_3N_4**
**PBE**	3.8 eV	3.6 eV	2.8 eV	3.0 eV
**HSE**	5.3 eV	5.4 eV	4.3 eV	4.4 eV

References

[1] H. Hahn, G. Frank, W. Klingler, *Zeitschrift für Anorg. und Allg. Chemie* **1955**, *279*, 271.

[2] O. V. München, J. Schneider, S. Haussühl, *Zeitschrift für Krist. - Cryst. Mater.* **1994**, *209*, 328.

[3] F. Liebau, *Structural Chemistry of Silicates*, Springer Berlin Heidelberg, Berlin, Heidelberg, **1985**.

[4] W. J. Duffin, J. H. C. Hogg, *Acta Crystallogr.* **1966**, *20*, 566.

[5] J. P. Paolini, *J. Comput. Chem.* **1990**, *11*, 1160.

[6] E. M. Layton, R. D. Kross, V. A. Fassel, *J. Chem. Phys.* **1956**, *25*, 135.

[7] F. H. Allen, O. Kennard, D. G. Watson, L. Brammer, A. G. Orpen, *J. Chem. Soc., Perkin Trans. 2* **1987**, *1–19*.

[8] F. Occelli, P. Loubeyre, R. LeToullec, *Nat. Mater.* **2003**, *2*, 151.

[9] E. Stavrou, S. Lobanov, H. Dong, A. R. Oganov, V. B. Prakapenka, Z. Konôpková, A. F. Goncharov, *Chem. Mater.* **2016**, *28*, 6925.

[10] M. I. Eremets, A. G. Gavriliuk, I. A. Trojan, D. A. Dzivenko, R. Boehler, *Nat. Mater.* **2004**, *3*, 558.

[11] D. Laniel, B. Winkler, T. Fedotenko, A. Pakhomova, S. Chariton, V. Milman, V. Prakapenka, L. Dubrovinsky, N. Dubrovinskaia, *Phys. Rev. Lett.* **2020**, *124*, 216001.

[12] H. Fujihisa, Y. Akahama, H. Kawamura, Y. Ohishi, Y. Gotoh, H. Yamawaki, M. Sakashita, S. Takeya, K. Honda, *Phys. Rev. Lett.* **2007**, *98*, 175501.

[13] D. Laniel, F. Trybel, A. Néri, Y. Yin, A. Aslandukov, T. Fedotenko, S. Khandarkhaeva, F. Tasnádi, S. Chariton, C. Giacobbe, E. L. Bright, M. Hanfland, V. Prakapenka, W. Schnick, I. A. Abrikosov, L. Dubrovinsky, N. Dubrovinskaia, *Chem. – A Eur. J.* **2022**, *28*, DOI 10.1002/chem.202201998.

[14] D. Laniel, "Cubic Gauche Nitrogen Experimental Crystal Structure Determination," DOI 10.5517/ccdc.csd.cc23bx2gcan be found under https://www.ccdc.cam.ac.uk/structures/Search?ccdc=1946895, **2019**.

[15] C. Ji, A. A. Adeleke, L. Yang, B. Wan, H. Gou, Y. Yao, B. Li, Y. Meng, J. S. Smith, V. B. Prakapenka, W. Liu, G. Shen, W. L. Mao, H. Mao, *Sci. Adv.* **2020**, *6*, eaba9206.

[16] T. Yamanaka, S. Morimoto, *Acta Crystallogr. Sect. B Struct. Sci.* **1996**, *52*, 232.

[17] P. C. Müller, C. Ertural, J. Hempelmann, R. Dronskowski, *J. Phys. Chem. C* **2021**, *125*, 7959.

[18] R. Nelson, C. Ertural, J. George, V. L. Deringer, G. Hautier, R. Dronskowski, *J. Comput. Chem.* **2020**, *41*, 1931.

[19] O. Hellman, I. A. Abrikosov, *Phys. Rev. B* **2013**, *88*, 144301.

[20] A. Togo, I. Tanaka, *Scr. Mater.* **2015**, *108*, 1.

[21] A. V. Lugovskoi, M. I. Katsnelson, A. N. Rudenko, *Phys. Rev. Lett.* **2019**, *123*, 176401.

[22] E. A. Stepanov, V. Harkov, M. Rösner, A. I. Lichtenstein, M. I. Katsnelson, A. N. Rudenko, *npj Comput. Mater.* **2022**, *8*, 118.

[23] M. I. Katsnelson, A. V. Trefilov, *Phys. Lett. A* **1985**, *109*, 109.

[24] M. Schüler, M. Rösner, T. O. Wehling, A. I. Lichtenstein, M. I. Katsnelson, *Phys. Rev. Lett.* **2013**, *111*, 1.

[25] F. Aryasetiawan, M. Imada, A. Georges, G. Kotliar, S. Biermann, A. I. Lichtenstein, *Phys. Rev. B* **2004**, *70*, 195104.

[26] D. Tomasino, J.-Y. Chen, M. Kim, C.-S. Yoo, *J. Chem. Phys.* **2013**, *138*, 094506.

[27] T. R. Ravindran, J. V. Badding, *J. Mater. Sci.* **2006**, *41*, 7145.

[28] K. H. Michaelian, K. E. Rieckhoff, E. M. Voigt, *Spectrosc. Lett.* **1977**, *10*, 99.
[29] O. V. Dolomanov, L. J. Bourhis, R. J. Gildea, J. A. K. Howard, H. Puschmann, *J. Appl. Crystallogr.* **2009**, *42*, 339.
[30] V. Petrícek, M. Dušek, L. Palatinus, *Zeitschrift fur Krist.* **2014**, *229*, 345.
[31] A. O. Lyakhov, A. R. Oganov, *Phys. Rev. B - Condens. Matter Mater. Phys.* **2011**, *84*, 2.
[32] X. Q. Chen, H. Niu, D. Li, Y. Li, *Intermetallics* **2011**, *19*, 1275.
[33] E. Mazhnik, A. R. Oganov, *J. Appl. Phys.* **2019**, *126*, 125109.
[34] F. Datchi, A. Dewaele, Y. Le Godec, P. Loubeyre, *Phys. Rev. B - Condens. Matter Mater. Phys.* **2007**, *75*, 1.
[35] A. Nagakubo, H. Ogi, H. Sumiya, K. Kusakabe, M. Hirao, *Appl. Phys. Lett.* **2013**, *102*, DOI 10.1063/1.4811789.
[36] A. Šimůnek, J. Vackář, *Phys. Rev. Lett.* **2006**, *96*, 085501.
[37] J. Chilleri, P. Siddiqua, M. S. Shur, S. K. O'Leary, *Appl. Phys. Lett.* **2022**, *120*, 122105.
[38] R. G. Munro, S. W. Freiman, T. L. Baker, *Fracture Toughness Data for Brittle Materials*, National Institute Of Standards And Technology, **1998**.
[39] H. Dong, A. R. Oganov, Q. Zhu, G.-R. Qian, *Sci. Rep.* **2015**, *5*, 9870.
[40] C. D. Clark, P. J. Dean, P V Harris, *Proc. R. Soc. London. Ser. A. Math. Phys. Sci.* **1964**, *277*, 312.
[41] Z. Liu, D. Li, S. Wei, Y. Liu, F. Tian, D. Duan, T. Cui, *Phys. Lett. A* **2019**, *1*, 125859.
