# Supplementary material for: Synthesis of Ultra‐Incompressible and Recoverable Carbon Nitrides Featuring CN4 Tetrahedra
Source: Adv Mater. 2023 Dec 10;36(3):2308030. doi: 10.1002/adma.202308030 (PMC11475261; doi:10.1002/adma.202308030)

checkCIF/PLATON report

Structure factors have been supplied for datablock(s) I

THIS REPORT IS FOR GUIDANCE ONLY. IF USED AS PART OF A REVIEW PROCEDURE FOR PUBLICATION, IT SHOULD NOT REPLACE THE EXPERTISE OF AN EXPERIENCED CRYSTALLOGRAPHIC REFEREE.

No syntax errors found. CIF dictionary Interpreting this report

Datablock: I

```
Bond precision:   N- C = 0.0020 A          Wavelength=0.28430

Cell:              a=3.4680(6)      b=3.4680(6)      c=6.8970(16)
                   alpha=90         beta=90          gamma=90
Temperature:       293 K

                Calculated                 Reported
Volume          82.95(3)                   82.95(3)
Space group     I -4 2 m                   I -4 2 m
Hall group      I -4 2                     I -4;2
Moiety formula  C3 N4                      ?
Sum formula     C3 N4                      C0.75 N1
Mr              92.07                      23.00
Dx,g cm-3       3.686                      3.686
Z               2                          8
Mu (mm-1)       0.078                      0.077
F000            92.0                       92.0
F000'           91.94
h,k,lmax        7,7,14                     6,6,12
Nref            244[ 152]                  133
Tmin,Tmax                                  0.674,1.000
Tmin'

Correction method= # Reported T Limits: Tmin=0.674 Tmax=1.000
AbsCorr = MULTI-SCAN

Data completeness= 0.88/0.55       Theta(max)= 17.600

                                                  wR2(reflections)=
R(reflections)= 0.0451( 81)                       wR= 0.0407( 133)

S = 1.370                  Npar= 11
```

The following ALERTS were generated. Each ALERT has the format
test-name_ALERT_alert-type_alert-level.
Click on the hyperlinks for more details of the test.

Alert level A

```
PLAT029_ALERT_3_A _diffrn_measured_fraction_theta_full value Low .      0.730 Why?
```

Author Response: This measurement was performed in a diamond anvil cell which, limits the theta range. Indeed, the diamond anvil cell's metallic body typically shadows more than 60% of the reflections.

Alert level C

```
PLAT041_ALERT_1_C Calc. and Reported SumFormula    Strings  Differ     Please Check
PLAT053_ALERT_1_C Minimum Crystal Dimension Missing (or Error) ...     Please Check
PLAT054_ALERT_1_C Medium  Crystal Dimension Missing (or Error) ...     Please Check
PLAT055_ALERT_1_C Maximum Crystal Dimension Missing (or Error) ...     Please Check
PLAT127_ALERT_1_C Implicit Hall Symbol  Inconsistent with Explicit     I -4;2 Check
```

Alert level G

```
ABSMU01_ALERT_1_G  Calculation of _exptl_absorpt_correction_mu
                not performed for this radiation type.
PLAT004_ALERT_5_G Polymeric Structure Found with Maximum Dimension          2 Info
PLAT005_ALERT_5_G No Embedded Refinement Details Found  in the CIF     Please Do !
PLAT040_ALERT_1_G No H-atoms in this Carbon Containing Compound ..     Please Check
PLAT045_ALERT_1_G Calculated and Reported Z Differ by a Factor ...      0.250 Check
PLAT092_ALERT_4_G Check: Wavelength Given is not Cu,Ga,Mo,Ag,In Ka    0.28430 Ang.
PLAT180_ALERT_4_G Check Cell Rounding: # of Values Ending with 0 =          3 Note
PLAT199_ALERT_1_G Reported _cell_measurement_temperature ..... (K)        293 Check
PLAT200_ALERT_1_G Reported   _diffrn_ambient_temperature ..... (K)        293 Check
PLAT432_ALERT_2_G Short Inter X...Y Contact  N1       ..C2       .       2.81 Ang.
                                                 -x,-y,z  =      2_555 Check
PLAT432_ALERT_2_G Short Inter X...Y Contact  N1       ..C2       .       2.88 Ang.
                                                 -x,y,-z  =      5_555 Check
PLAT432_ALERT_2_G Short Inter X...Y Contact  N1       ..C1       .       2.88 Ang.
                                       1/2-y,1/2+x,1/2-z  =     12_555 Check
PLAT432_ALERT_2_G Short Inter X...Y Contact  N1       ..C1       .       2.91 Ang.
                                                -x,1-y,z  =      2_565 Check
PLAT432_ALERT_2_G Short Inter X...Y Contact  N1       ..C1       .       2.91 Ang.
                                                1-y,-x,z  =      7_655 Check
PLAT432_ALERT_2_G Short Inter X...Y Contact  N1       ..C2       .       2.93 Ang.
                                                 1+x,y,z  =      1_655 Check
PLAT432_ALERT_2_G Short Inter X...Y Contact  N1       ..C2       .       2.93 Ang.
                                       1/2-x,1/2+y,1/2-z  =     13_555 Check
PLAT432_ALERT_2_G Short Inter X...Y Contact  C1       ..C2       .       2.45 Ang.
                                                 -x,y,-z  =      5_555 Check
PLAT432_ALERT_2_G Short Inter X...Y Contact  C1       ..C2       .       2.45 Ang.
                                                 -x,-y,z  =      2_555 Check
PLAT432_ALERT_2_G Short Inter X...Y Contact  C1       ..C2       .       2.45 Ang.
                                                 -y,x,-z  =      4_555 Check
PLAT432_ALERT_2_G Short Inter X...Y Contact  C2       ..C2       .       2.45 Ang.
```

```
                                          1/2-x,1/2+y,1/2-z  =     13_555 Check
PLAT808_ALERT_5_G No Parseable SHELXL Style Weighting Scheme Found     Please Check
PLAT883_ALERT_1_G No Info/Value for _atom_sites_solution_primary .     Please Do !
PLAT910_ALERT_3_G Missing # of FCF Reflection(s) Below Theta(Min).          3 Note
PLAT911_ALERT_3_G Missing FCF Refl Between Thmin & STh/L=    0.600          9 Report
PLAT912_ALERT_4_G Missing # of FCF Reflections Above STh/L=  0.600         38 Note
PLAT929_ALERT_5_G No Weight Pars,Obs and Calc R1,wR2,S not Checked          ! Info
PLAT952_ALERT_5_G Calculated (ThMax) and CIF-Reported Lmax Differ.          2 Units
PLAT958_ALERT_1_G Calculated (ThMax) and Actual (FCF) Lmax Differ.          2 Units
PLAT966_ALERT_5_G Note: Non-Standard (i.e. 2.0) OMIT Threshold  of        3.0 Sig(I)
```

```
   1 ALERT level A = Most likely a serious problem - resolve or explain
   0 ALERT level B = A potentially serious problem, consider carefully
   5 ALERT level C = Check. Ensure it is not caused by an omission or oversight
  29 ALERT level G = General information/check it is not something unexpected

  12 ALERT type 1 CIF construction/syntax error, inconsistent or missing data
  11 ALERT type 2 Indicator that the structure model may be wrong or deficient
   3 ALERT type 3 Indicator that the structure quality may be low
   3 ALERT type 4 Improvement, methodology, query or suggestion
   6 ALERT type 5 Informative message, check
```

It is advisable to attempt to resolve as many as possible of the alerts in all categories. Often the minor alerts point to easily fixed oversights, errors and omissions in your CIF or refinement strategy, so attention to these fine details can be worthwhile. In order to resolve some of the more serious problems it may be necessary to carry out additional measurements or structure refinements. However, the purpose of your study may justify the reported deviations and the more serious of these should normally be commented upon in the discussion or experimental section of a paper or in the "special_details" fields of the CIF. checkCIF was carefully designed to identify outliers and unusual parameters, but every test has its limitations and alerts that are not important in a particular case may appear. Conversely, the absence of alerts does not guarantee there are no aspects of the results needing attention. It is up to the individual to critically assess their own results and, if necessary, seek expert advice.

Publication of your CIF in IUCr journals

A basic structural check has been run on your CIF. These basic checks will be run on all CIFs submitted for publication in IUCr journals (*Acta Crystallographica*, *Journal of Applied Crystallography*, *Journal of Synchrotron Radiation*); however, if you intend to submit to *Acta Crystallographica Section C* or *E* or *IUCrData*, you should make sure that full publication checks are run on the final version of your CIF prior to submission.

Publication of your CIF in other journals

Please refer to the *Notes for Authors* of the relevant journal for any special instructions relating to CIF submission.

PLATON version of 06/07/2023; check.def file version of 30/06/2023

Datablock I - ellipsoid plot

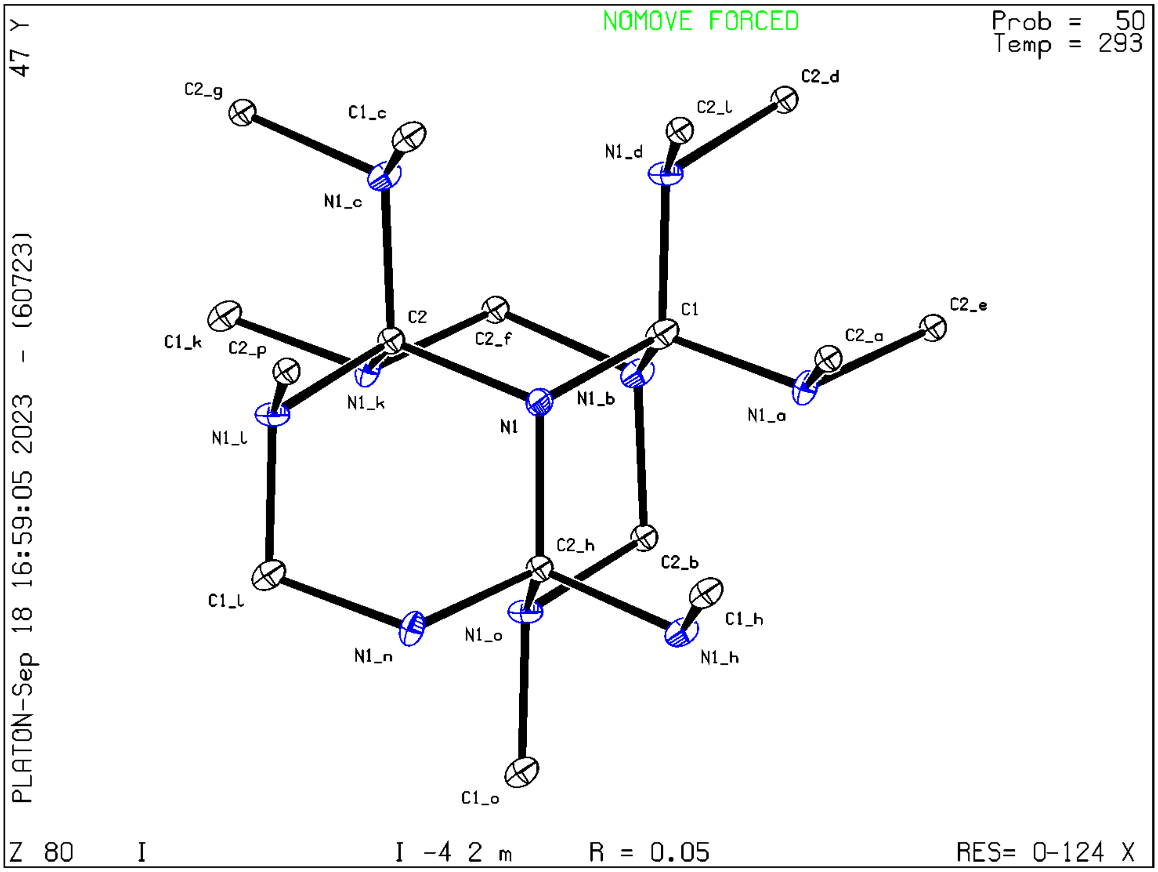

Supplement: Supplementary file 2 — Supporting Information [file C3N4_1bar_ID11-April2022_DL068_p06_s3_dom2_v1.pdf › C3N4_1bar_ID11-April2022_DL068_p06_s3_dom2_v1.pdf]
