# Supplementary material for: Synthesis of Ultra‐Incompressible and Recoverable Carbon Nitrides Featuring CN4 Tetrahedra
Source: Adv Mater. 2023 Dec 10;36(3):2308030. doi: 10.1002/adma.202308030 (PMC11475261; doi:10.1002/adma.202308030)

checkCIF/PLATON report

Structure factors have been supplied for datablock(s) I

THIS REPORT IS FOR GUIDANCE ONLY. IF USED AS PART OF A REVIEW PROCEDURE FOR PUBLICATION, IT SHOULD NOT REPLACE THE EXPERTISE OF AN EXPERIENCED CRYSTALLOGRAPHIC REFEREE.

No syntax errors found. CIF dictionary Interpreting this report

Datablock: I

```
Bond precision:   C-C = 0.0050 A          Wavelength=0.28430

Cell:               a=5.282(4)      b=3.9353(12)       c=2.3719(9)
                    alpha=90        beta=90            gamma=90
Temperature:        293 K

                 Calculated                      Reported
Volume           49.30(4)                        49.30(4)
Space group      P n n m                         P n n m
Hall group       -P 2 2n                         -P -2xabc;-
Moiety formula   C N                             ?
Sum formula      C N                             C1 N1
Mr               26.02                           26.00
Dx,g cm-3        3.506                           3.505
Z                4                               4
Mu (mm-1)        0.074                           0.073
F000             52.0                            52.0
F000'            51.97
h,k,lmax         11,8,5                          6,7,4
Nref             273                             115
Tmin,Tmax
Tmin'

Correction method= Not given

Data completeness= 0.421          Theta(max)= 17.450

                                                      wR2(reflections)=
R(reflections)= 0.0484( 50)                           wR= 0.0475( 115)

S = 0.910                 Npar= 10
```

The following ALERTS were generated. Each ALERT has the format
test-name_ALERT_alert-type_alert-level.
Click on the hyperlinks for more details of the test.

Alert level A

```
PLAT029_ALERT_3_A _diffrn_measured_fraction_theta_full value Low .      0.590 Why?
```

Author Response: This measurement was performed in a diamond anvil cell which, limits the theta range. Indeed, the diamond anvil cell's metallic body typically shadows more than 60% of the reflections.

Alert level C

```
PLAT041_ALERT_1_C Calc. and Reported SumFormula    Strings  Differ     Please Check
PLAT053_ALERT_1_C Minimum Crystal Dimension Missing (or Error) ...     Please Check
PLAT054_ALERT_1_C Medium  Crystal Dimension Missing (or Error) ...     Please Check
PLAT055_ALERT_1_C Maximum Crystal Dimension Missing (or Error) ...     Please Check
PLAT127_ALERT_1_C Implicit Hall Symbol  Inconsistent with Explicit -P -2xabc; Check
PLAT340_ALERT_3_C Low Bond Precision on  C-C Bonds ...............      0.005 Ang.
PLAT913_ALERT_3_C Missing # of Very Strong Reflections in FCF ....          4 Note
PLAT920_ALERT_1_C Theta(Max)  in CIF and FCF Differ by ..........        0.26 Degree
```

Alert level G

```
ABSMU01_ALERT_1_G  Calculation of _exptl_absorpt_correction_mu
                not performed for this radiation type.
PLAT004_ALERT_5_G Polymeric Structure Found with Maximum Dimension          3 Info
PLAT005_ALERT_5_G No Embedded Refinement Details Found  in the CIF     Please Do !
PLAT040_ALERT_1_G No H-atoms in this Carbon Containing Compound ..     Please Check
PLAT092_ALERT_4_G Check: Wavelength Given is not Cu,Ga,Mo,Ag,In Ka    0.28430 Ang.
PLAT199_ALERT_1_G Reported _cell_measurement_temperature ..... (K)        293 Check
PLAT200_ALERT_1_G Reported   _diffrn_ambient_temperature ..... (K)        293 Check
PLAT432_ALERT_2_G Short Inter X...Y Contact  N1       ..C1        .       2.42 Ang.
                                              1+x,1+y,1+z  =      1_666 Check
PLAT432_ALERT_2_G Short Inter X...Y Contact  N1       ..C1        .       2.42 Ang.
                                                1+x,1+y,z  =      1_665 Check
PLAT432_ALERT_2_G Short Inter X...Y Contact  N1       ..C1        .       2.53 Ang.
                                        1/2-x,1/2+y,1/2-z  =      3_555 Check
PLAT432_ALERT_2_G Short Inter X...Y Contact  N1       ..C1        .       2.80 Ang.
                                       1/2+x,1/2-y,-1/2-z  =      4_554 Check
PLAT432_ALERT_2_G Short Inter X...Y Contact  N1       ..C1        .       2.80 Ang.
                                        1/2+x,1/2-y,3/2-z  =      4_556 Check
PLAT432_ALERT_2_G Short Inter X...Y Contact  N1       ..C1        .       2.88 Ang.
                                        1/2+x,3/2-y,1/2-z  =      4_565 Check
PLAT432_ALERT_2_G Short Inter X...Y Contact  N1       ..C1        .       2.99 Ang.
                                        3/2-x,1/2+y,1/2-z  =      3_655 Check
PLAT432_ALERT_2_G Short Inter X...Y Contact  C1       ..C1        .       2.37 Ang.
                                                  x,y,1+z  =      1_556 Check
PLAT432_ALERT_2_G Short Inter X...Y Contact  C1       ..C1        .       2.37 Ang.
                                                 x,y,-1+z  =      1_554 Check
PLAT432_ALERT_2_G Short Inter X...Y Contact  C1       ..C1        .       2.56 Ang.
                                      1/2-x,-1/2+y,-1/2-z  =      3_544 Check
```

```
PLAT432_ALERT_2_G Short Inter X...Y Contact  C1       ..C1       .       2.56 Ang.
                                         1/2-x,1/2+y,1/2-z  =      3_555 Check
PLAT432_ALERT_2_G Short Inter X...Y Contact  C1       ..C1       .       2.56 Ang.
                                        1/2-x,1/2+y,-1/2-z  =      3_554 Check
PLAT432_ALERT_2_G Short Inter X...Y Contact  C1       ..C1       .       2.56 Ang.
                                        1/2-x,-1/2+y,1/2-z  =      3_545 Check
PLAT432_ALERT_2_G Short Inter X...Y Contact  C1       ..C1       .       2.84 Ang.
                                                -x,-y,-1+z  =      2_554 Check
PLAT432_ALERT_2_G Short Inter X...Y Contact  C1       ..C1       .       2.84 Ang.
                                                 -x,-y,1+z  =      2_556 Check
PLAT808_ALERT_5_G No Parseable SHELXL Style Weighting Scheme Found     Please Check
PLAT883_ALERT_1_G No Info/Value for _atom_sites_solution_primary .     Please Do !
PLAT910_ALERT_3_G Missing # of FCF Reflection(s) Below Theta(Min).          3 Note
PLAT911_ALERT_3_G Missing FCF Refl Between Thmin & STh/L=    0.600         27 Report
PLAT912_ALERT_4_G Missing # of FCF Reflections Above STh/L=  0.600         58 Note
PLAT929_ALERT_5_G No Weight Pars,Obs and Calc R1,wR2,S not Checked          ! Info
PLAT950_ALERT_5_G Calculated (ThMax) and CIF-Reported Hmax Differ           5 Units
PLAT956_ALERT_1_G Calculated (ThMax) and Actual (FCF) Hmax Differ           5 Units
PLAT966_ALERT_5_G Note: Non-Standard (i.e. 2.0) OMIT Threshold  of        3.0 Sig(I)
```

```
   1 ALERT level A = Most likely a serious problem - resolve or explain
   0 ALERT level B = A potentially serious problem, consider carefully
   8 ALERT level C = Check. Ensure it is not caused by an omission or oversight
  31 ALERT level G = General information/check it is not something unexpected

  12 ALERT type 1 CIF construction/syntax error, inconsistent or missing data
  15 ALERT type 2 Indicator that the structure model may be wrong or deficient
   5 ALERT type 3 Indicator that the structure quality may be low
   2 ALERT type 4 Improvement, methodology, query or suggestion
   6 ALERT type 5 Informative message, check
```

It is advisable to attempt to resolve as many as possible of the alerts in all categories. Often the minor alerts point to easily fixed oversights, errors and omissions in your CIF or refinement strategy, so attention to these fine details can be worthwhile. In order to resolve some of the more serious problems it may be necessary to carry out additional measurements or structure refinements. However, the purpose of your study may justify the reported deviations and the more serious of these should normally be commented upon in the discussion or experimental section of a paper or in the "special_details" fields of the CIF. checkCIF was carefully designed to identify outliers and unusual parameters, but every test has its limitations and alerts that are not important in a particular case may appear. Conversely, the absence of alerts does not guarantee there are no aspects of the results needing attention. It is up to the individual to critically assess their own results and, if necessary, seek expert advice.

Publication of your CIF in IUCr journals

A basic structural check has been run on your CIF. These basic checks will be run on all CIFs submitted for publication in IUCr journals (*Acta Crystallographica*, *Journal of Applied Crystallography*, *Journal of Synchrotron Radiation*); however, if you intend to submit to *Acta Crystallographica Section C* or *E* or *IUCrData*, you should make sure that full publication checks are run on the final version of your CIF prior to submission.

Publication of your CIF in other journals

Please refer to the *Notes for Authors* of the relevant journal for any special instructions relating to CIF submission.

PLATON version of 06/07/2023; check.def file version of 30/06/2023

Datablock I - ellipsoid plot

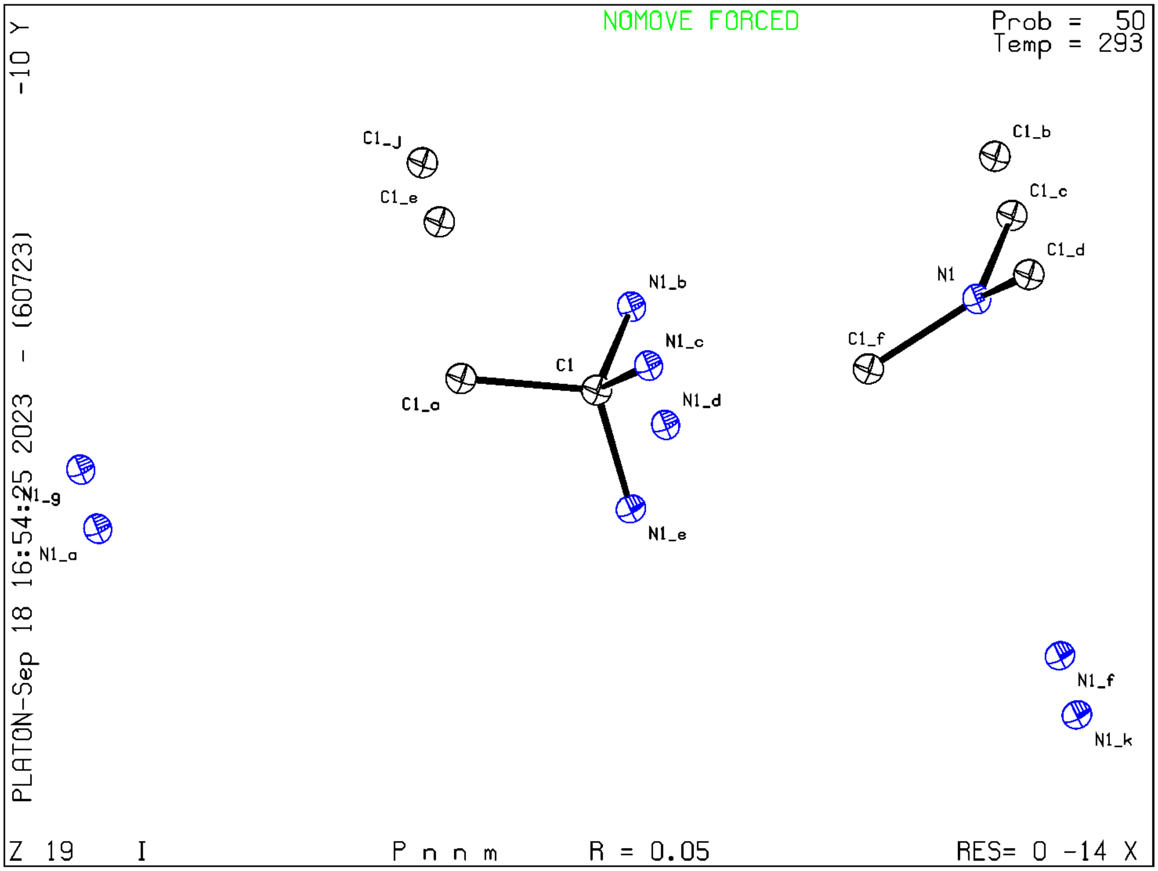

Supplement: Supplementary file 2 — Supporting Information [file CN_1bar_ID11-April2022_DL067_p04_s1_dom1_v1.pdf › CN_1bar_ID11-April2022_DL067_p04_s1_dom1_v1.pdf]
